# Coordination chemistry and photoswitching of dinuclear macrocyclic cadmium-, nickel-, and zinc complexes containing azobenzene carboxylato co-ligands

**DOI:** 10.3762/bjoc.15.81

**Published:** 2019-04-03

**Authors:** Jennifer Klose, Tobias Severin, Peter Hahn, Alexander Jeremies, Jens Bergmann, Daniel Fuhrmann, Jan Griebel, Bernd Abel, Berthold Kersting

**Affiliations:** 1 Institut für Anorganische Chemie, Universität Leipzig, Johannisallee 29, 04103 Leipzig, Germany, Fax: +49(0)341-97-36199; 2 Leibniz-Institut für Oberflächenmodifizierung e. V., Abteilung Funktionale Oberflächen, D-04318 Leipzig, Germany; 3 Wilhelm-Ostwald-Institut für Physikalische und Theoretische Chemie, Universität Leipzig, Linnéstraße 2, D-04103 Leipzig, Germany

**Keywords:** azobenzene carboxylates, cadmium, complexes, macrocyclic ligands, nickel, zinc

## Abstract

The synthesis of mixed-ligand complexes of the type [M_2_L(μ-L')]^+^, where L represents a 24-membered macrocyclic hexaaza-dithiophenolate ligand, L' is an azobenzene carboxylate co-ligand, and M = Cd(II), Ni(II) or Zn(II), is reported. A series of new complexes were synthesized, namely [M_2_L(μ-L')]^+^ (L' = azo-H, M = Cd (**1**), Ni (**2**); L' = azo-OH, M = Zn (**3**), Ni (**4**); L' = azo-NMe_2_, M = Zn (**5**), Cd (**6**), Ni (**7**); L' = azo-CO_2_Me, M = Cd (**8**), Ni (**9**)), and characterized by elemental analysis, electrospray ionization mass spectrometry (ESIMS), IR, UV–vis and NMR spectroscopy (for diamagnetic Zn and Cd complexes) and X-ray single crystal structure analysis. The crystal structures of **3'** and **5–8** display an isostructural series of compounds with bridging azobenzene carboxylates in the *trans* form. The paramagnetic Ni complexes **2**, **4** and **7** reveal a weak ferromagnetic exchange interaction with magnetic exchange coupling constant values between 21 and 23 cm^−1^ (H = −2*J*S_1_S_2_). Irradiation of **1** with λ = 365 nm reveals a photoisomerization of the co-ligand from the *trans* to the *cis* form.

## Introduction

**Figure 1:**
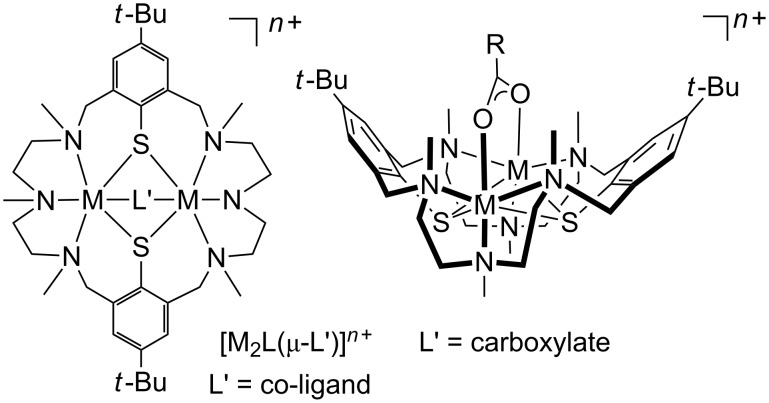
Left: Mixed ligand complexes of the type [M_2_L(μ-L')]*^n^*^+^ supported by the macrocyclic ligand H_2_L (M = divalent or trivalent transition metal). Right: Perspective view of the bowl-shaped structure of the corresponding carboxylato complexes.

The macrocyclic N_6_S_2_ donor ligand H_2_L is an effective dinucleating ligand that supports a large number of mixed ligand complexes of the type [M_2_L(μ-O_2_CR)]^+^ [1], where M is a divalent or trivalent metal ion (M = Mn^2+^, Fe^2+^, Co^2+^, Co^3+^, Ni^2+^, Zn^2+^ and Cd^2+^) and μ-O_2_CR is a μ_1,3_-bridging carboxylato ligand (Figure 1). The encapsulation of a functional carboxylate ion by the bowl-shaped [M_2_L] fragment can drastically alter its structural properties and reactivities. Prominent examples are the *cis*-bromination of α,β-unsaturated carboxylato ligands [2], regioselective Diels–Alder reactions of encapsulated dienoate ligands [3], and the stabilization of unusual co-ligand conformations, respectively [4]. The physicochemical properties of the bound guest molecules are also greatly affected. Thus, complexation of naphthalene diimide carboxylato ligands leads to a substantial (>95%) quenching of the diimide fluorescence [5], and incorporation of a Fe(CpCO_2_H)_2_ unit leads to a significant anodic shift of the metallocene’s redox potential [6].

**Figure 2:**
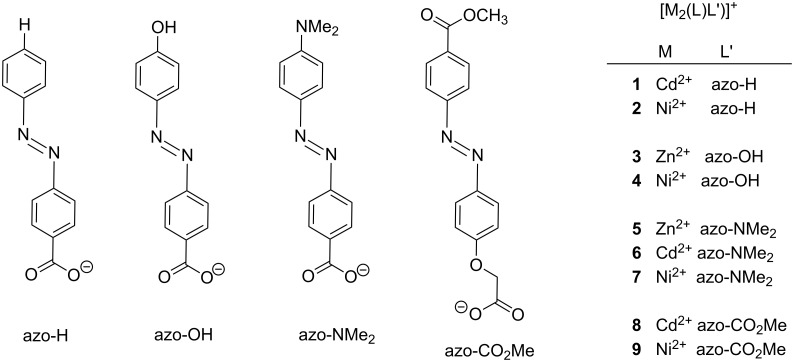
Synthesized compounds and their labels.

The facile formation of such compounds and their unusual properties led us to synthesize derivatives bearing azobenzene carboxylates as bridging co-ligands. A series of mixed-ligand complexes of the type [M_2_L(μ-L')]^+^, where M = Zn(II), Ni(II), or Cd(II), L' = *p*-azobenzene carboxylate (azo-H), *p*'-hydroxy-*p*-azobenzene carboxylate (azo-OH), *p*'-dimethylamine-*p*-azobenzene carboxylate (azo-NMe_2_), and *p*'-(methoxycarbonyl)azobenzene-*p*-oxymethylcarboxylic acid (azo-CO_2_Me) were synthesized (Figure 2). Their preparation and structural characterization by NMR, IR, UV–vis spectroscopy, SQUID magnetometry and X-ray crystallography is reported herein. Preliminary results concerning the photoisomerization of compound **1** are reported as well.

## Results and Discussion

### Synthesis of ligands and metal complexes

**Scheme 1:**
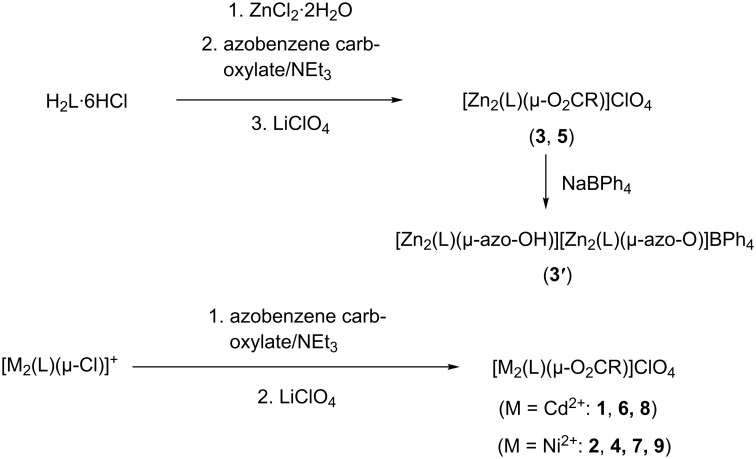
Synthesis of complexes **1–9**.

Scheme 1 shows the synthetic procedures. The yellow-orange colored zinc complexes (**3**, **5**) were obtained directly from stoichiometric complexation reactions between H_2_L·6HCl, Zn(OAc)_2_·2H_2_O, and the corresponding azobenzene carboxylate ion (prepared in situ from the free acid by deprotonation with NEt_3_ as a base) in methanol. The green-brown nickel (**2**, **4**, **7** and **9**) and red-orange colored cadmium complexes **1**, **6** and **8**, on the other hand, were prepared by substitution reactions involving the known chlorido-bridged [Ni_2_L(μ-Cl)]^+^ or [Cd_2_L(μ-Cl)]^+^ precursors and the respective azobenzene carboxylates. All complexes were isolated as ClO_4_^−^ salts with yields between 76% and 91%. The perchlorate salts are stable in air both in solution and in the solid state, and are very soluble in a range of common polar organic solvents (CH_3_CN, EtOH, MeOH). The new compounds gave satisfactory elemental analyses and were characterized by mass spectrometry, spectroscopic methods (IR, UV–vis, ^1^H and ^13^C NMR spectroscopy) and in case of **3'** and **5–8** also by X-ray crystal structure analysis.

### Characterization of complexes

#### IR and NMR spectroscopy

Table 1 lists selected analytical data for the synthesized compounds. The infrared spectra of **1–9** were recorded in the 4000**–**400 cm^−1^ spectral range. The IR spectra display in each case two strong bands, one between 1620**–**1590 cm^−1^ and the other in the 1419**–**1395 cm^−1^ range. These bands can be assigned to the asymmetric (*ν*_as_(RCO_2_^−^)) and symmetric stretching vibrations (*ν*_s_(RCO_2_^−^)) of the carboxylato co-ligands [1]. Coordination is implied by the fact that the *ν*_as_(RCO_2_^−^) frequencies are significantly red-shifted by 78**–**103 cm^−1^ to lower wavenumbers. A bathochromic shift of 14**–**32 cm^−1^ is also clearly observed for the *ν*_s_(RCO_2_^–^) frequencies. Notice that Δ = *ν*_as_(RCO_2_^−^) − *ν*_s_(RCO_2_^−^) is invariably larger than 180 cm^−1^, a clear indication that the co-ligand is in the bridging mode [7–9]. Another prominent feature is the intense band around 1100 cm^–1^, which can be readily assigned to a vibration of the ClO_4_^−^ ion (ν_3_F_4_). An unambiguous assignment of the N=N vibration of the azo group was not possible due to the large number of overlapping bands in the fingerprint region.

**Table 1:** Selected infrared data for compounds **1–9**.^a^

complex	*ν*_as_ RCO_2_^−^ [cm^−1^]	*ν*_s_ RCO_2_^−^ [cm^−1^]	Δ*ν* = (*ν*_as_ – *ν*_s_) [cm^−1^]

**1** [Cd_2_L(µ-azo-H)]^+^	1599	1395	204
**2** [Ni_2_L(µ-azo-H)]^+^	1602	1401	201
**3** [Zn_2_L(µ-azo-OH)]^+^	1600	1404	196
**4** [Ni_2_L(µ-azo-OH)]^+^	1590	1407	183
**5** [Zn_2_L(µ-azo-NMe_2_)]^+^	1599	1398	201
**6** [Cd_2_L(µ-azo-NMe_2_)]^+^	1598	1401	197
**7** [Ni_2_L(µ-azo-NMe_2_)]^+^	1599	1400	199
**8** [Cd_2_L(µ-azo-CO_2_Me)]^+^	1614	1412	202
**9** [Ni_2_L(µ-azo-CO_2_Me)]^+^	1620	1419	201

^a^The IR data refer to the solid ClO_4_^−^ salts.

**Figure 3:**
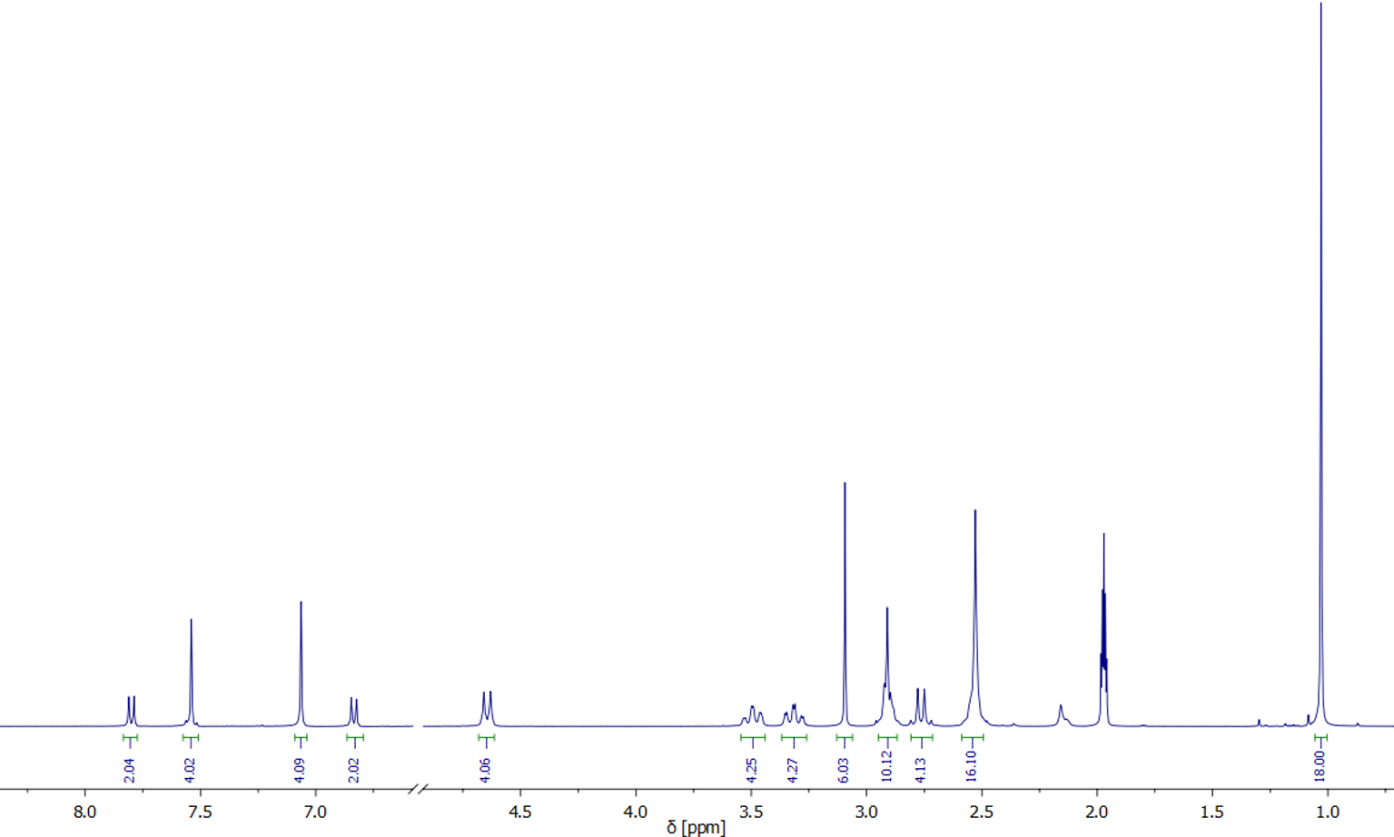
^1^H NMR spectrum of **6** in CD_3_CN at 295 K (1.0–8.0 ppm). The resonances and assignments are listed in Table 2 and Table S4 in Supporting Information File 1.

To determine their structures in solution the diamagnetic zinc and cadmium complexes were subjected to ^1^H and ^13^C NMR spectroscopic studies. Table 2, and Tables S4 and S5 (Supporting Information File 1) list the ^1^H and ^13^C NMR spectroscopic data for complexes **1**, **3**, **5**, **6** and **8**. The data for [Zn_2_L(μ-OAc)]^+^ and [Cd_2_L(μ-OAc)]^+^ have been reported previously and are included for comparative purposes [10–11]. Figure 3 displays the ^1^H NMR spectrum of [Cd_2_L(μ-azo-NMe_2_)]^+^ (**6**) in CD_3_CN which is representative for all complexes. Only one set of signals is observed, showing that all compounds exist as single isomers in solution. Thus, the six *N*-methyl groups give rise to two singlets (one for the methyl protons on the benzylic nitrogen atoms (NBzCH_3_) and one for the methyl protons on the central amine nitrogen of the linking diethylenetriamine units (NCH_3_)). Note that the four aromatic protons (ArH) and the *tert*-butyl protons [C(CH_3_)_3_] appear as singlets. The remaining six signals can be assigned to the methylene protons of the linking diethylenetriamine chains (two doublets for the benzylic CH_2_ and four multiplets for linking CH_2_ groups). The ^1^H NMR data are indicative of a *C*_2_*_v_* symmetric structure of the [Cd_2_L]^2+^ fragment in solution, as in [Cd_2_L(μ-Cl)]^+^ and other carboxylato-bridged Cd_2_ complexes supported by this macrocycle [10,12]. The remaining four signals (two doublets and one singlet for aromatic and one singlet for aliphatic CH_3_ protons) can be attributed to the azobenzene co-ligand. These were readily assigned with the aid of APT, HSQC, HMBC and COSY spectra. The ^13^C NMR spectrum of complex **6** is also in agreement with *C*_2_*_v_* symmetry revealing only 11 signals (seven for the aliphatic and four for the aromatic carbon atoms) for the 38 carbon atoms of the [Cd_2_L]^2+^ fragment.

**Table 2:** Comparison of ^1^H NMR resonances of the azobenzene co-ligands of the zinc (**3**, **5**) and cadmium complexes (**1**, **6**, **8**).^a^

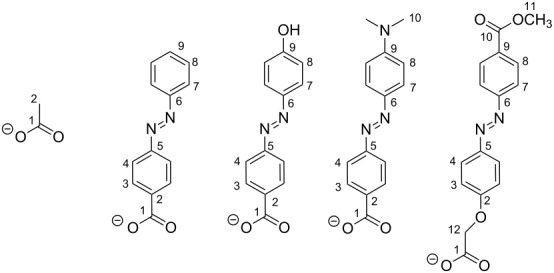				

[ZnL(OAc)]^b^ [4,10] [CdL(OAc)]^b^ [4]	**1**^c^	**3**^d^	**5**^c^, **6**^b^	**8**^c^

0.85 (C^2^H_3_) 0.98 (C^2^H_3_)	7.48–7.52 (C^9^H)	6.86 C^8^H	3.08 (C^10^H_3_) 3.06 (C^10^H_3_)	3.60 (OC^12^H_2_)
	7.48–7.52 (C^8^H)	7.31 C^3^H	6.74 (C^8^H) 6.81 (C^8^H)	3.93 (OC^11^H_3_)
	7.48–7.52 (C^3^H)	7.46 C^4^H	7.34 (C^3^H) 7.51 (C^3^H)	6.44 (C^3^H)
	7.63 (C^4^H)	7.67 C^7^H	7.49 (C^4^H) 7.51 (C^4^H)	7.72 (C^4^H)
	7.86 (C^7^H)		7.79 (C^7^H) 7.77 (C^7^H)	7.91 (C^8^H)
				8.17 (C^7^H)

^a^NMR data correspond to the ClO_4_^−^ salts. ^b^Solvent: CD_3_CN. ^c^Solvent: CD_2_Cl_2_. ^d^Solvent: (CD_3_)_2_SO.

### X-ray crystallography

**Figure 4:**
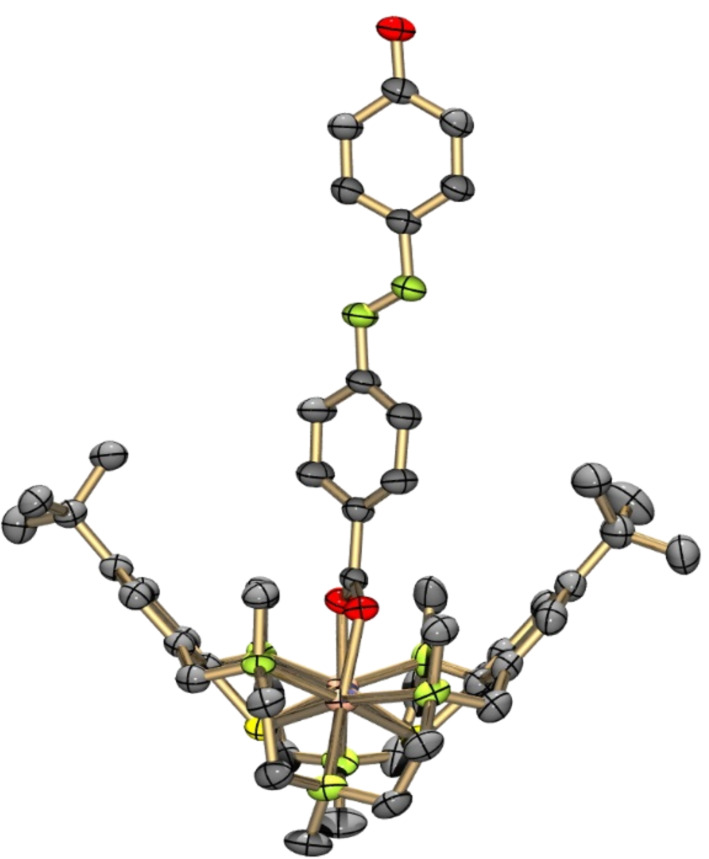
Structure of the [Zn_2_L(μ-azo-OH)]^+^ cation in crystals of [Zn_2_L(μ-azo-OH)][Zn_2_L(μ-azo-O)]·4MeCN·3H_2_O (**3'**·4MeCN·3H_2_O)*.* Hydrogen atoms omitted for clarity. Thermal ellipsoids are drawn at the 50% probability level (color codes: C dark grey, H pale gray, N green/or blue, O red, S yellow, metals flesh (Zn, Cd) or green (Ni)).

**Figure 5:**
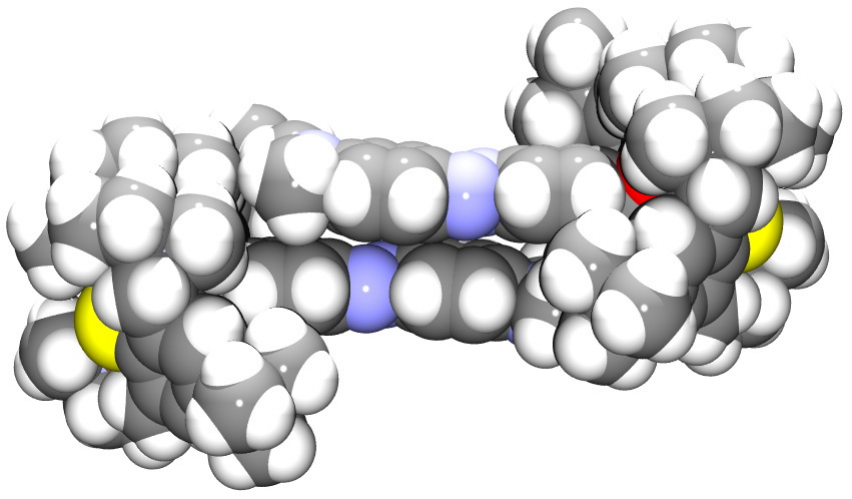
Space filling representation of the packing of two symmetry-related [Zn_2_L(μ-azo-NMe_2_)]^+^ cations in crystals of **5**·1.5MeCN*.* The ClO_4_^−^ anions and solvate molecules are omitted for clarity.

**Figure 6:**
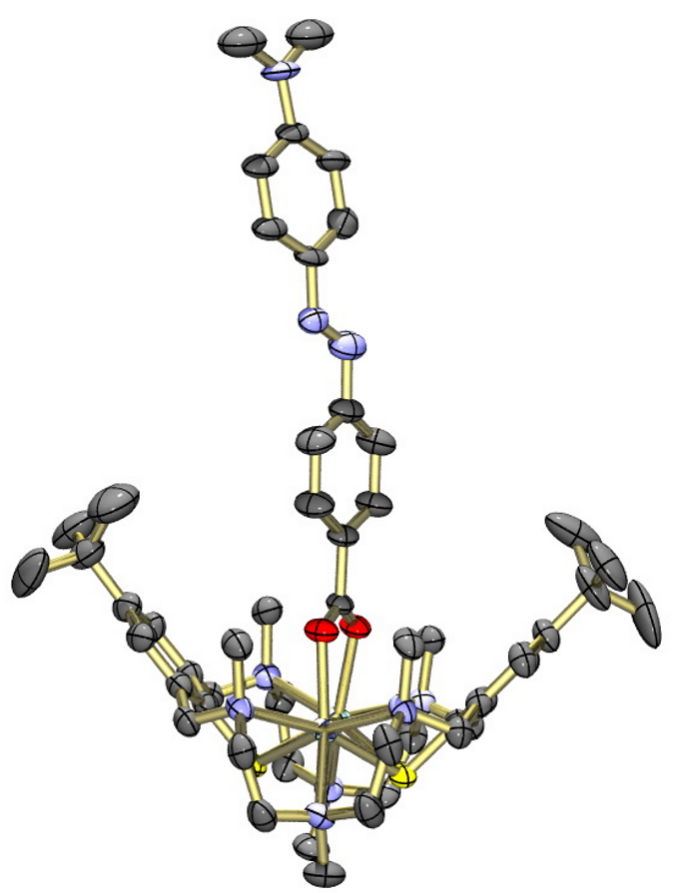
Structure of the [Cd_2_L(μ-azo-NMe_2_)]^+^ cation in crystals of [Cd_2_L(μ-azo-NMe_2_)]ClO_4_·0.5MeOH (**6**·0.5MeOH)*.* Only one orientation of the disordered azobenzene carboxylato co-ligand is displayed. Hydrogen atoms omitted for clarity. Thermal ellipsoids are drawn at the 50% probability level.

**Figure 7:**
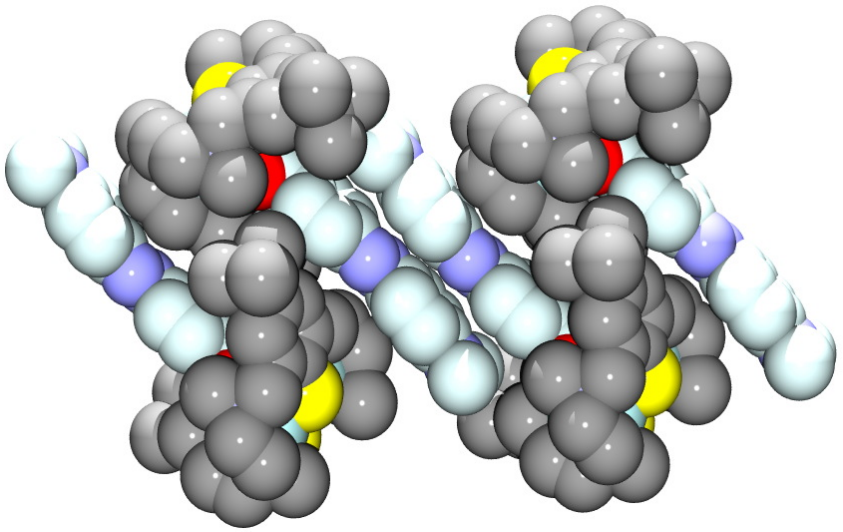
Space filling representation of the packing of four [Cd_2_L(μ-azo-NMe_2_)]^+^ cations in crystals of **6**·0.5MeOH*.* H atoms, ClO_4_^−^ anions and solvate molecules are omitted for clarity. The C atoms of the azobenzene moieties are in pale blue.

**Figure 8:**
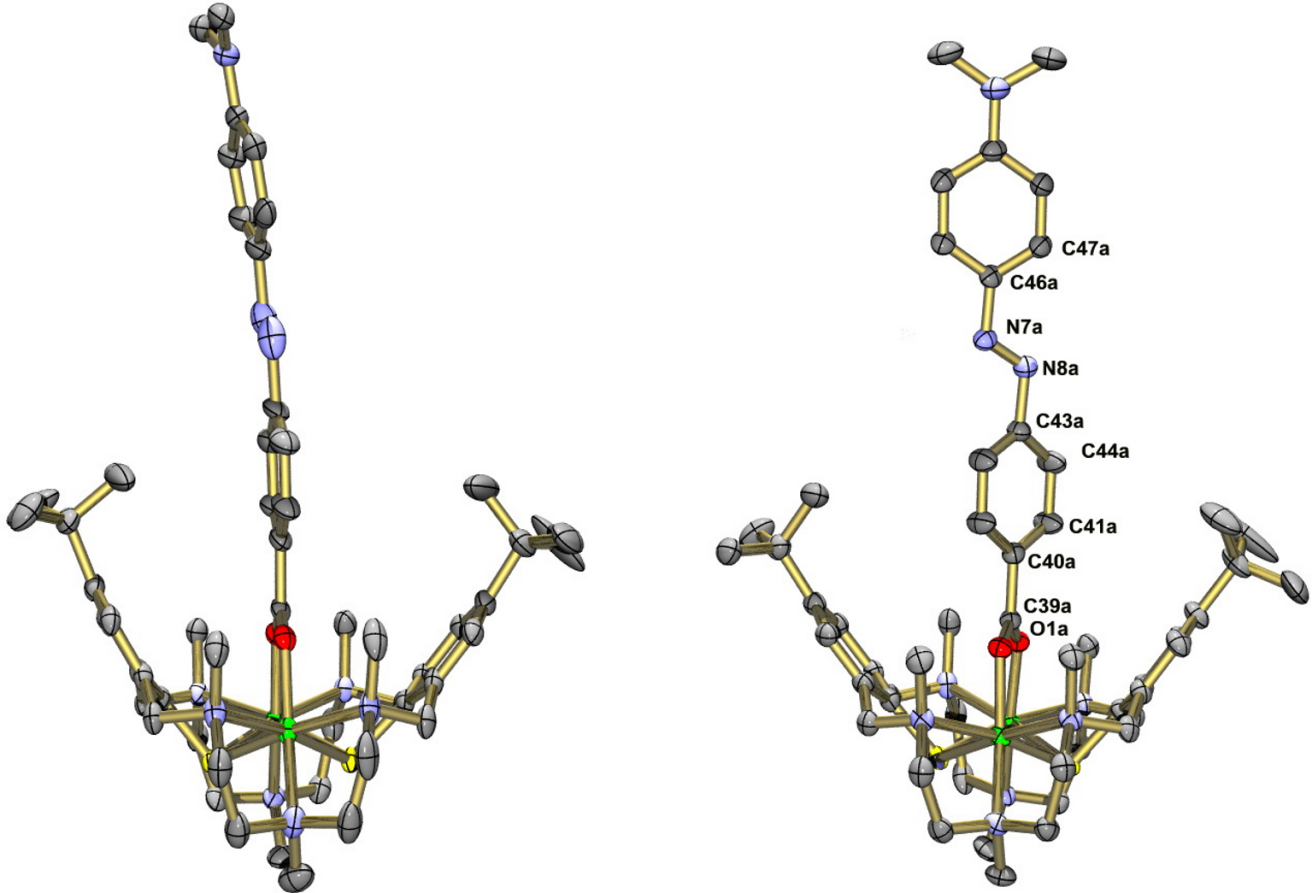
Structures of the two crystallographically independent [Ni_2_L(μ-azo-NMe_2_)]^+^ cations A (left) and B (right) in crystals of [Ni_2_L(μ-azo-NMe_2_)]ClO_4_·8EtOH (**7**·8EtOH). Only one orientation of the disordered azo-carboxylato co-ligand is displayed. Hydrogen atoms omitted for clarity. Thermal ellipsoids are drawn at the 50% probability level.

Further confirmation regarding the composition and structures of the complexes were obtained by single crystal X-ray diffraction studies. Single crystals could be obtained for the tetraphenylborates **3'** and **8** and perchlorate salts **5–7**. The molecular structures of the complexes are displayed in Figures 4**–**8. Selected bond lengths are given in Table S6, Supporting Information File 1.

#### Description of the crystal structures

**[Zn_2_L(μ-azo-OH)][Zn_2_L(μ-azo-O)]·BPh_4_·4MeCN·3H_2_O (3'·4MeCN·3H_2_O):** Suitable crystals of [Zn_2_L(μ-azo-OH)]ClO_4_ (**3**) could not be obtained. However, the addition of NaBPh_4_ to a solution of **3** in MeCN led to crystallization of [Zn_2_L(μ-azo-OH)][Zn_2_L(μ-azo-O)]·BPh_4_·4MeCN·3H_2_O (**3'***·*4MeCN·3H_2_O). The asymmetric unit contains one [Zn_2_L(μ-azo-OH)]^+^ cation (Figure 4), one [Zn_2_L(μ-azo-O)] neutral complex (comprising a doubly deprotonated co-ligand), one BPh_4_^−^ anion, and MeCN and H_2_O solvate molecules. Both complexes are structurally very similar and the corresponding bond lengths lie within narrow ranges (Table S6 in Supporting Information File 1). The difference between the two molecules concerns the twisting angle between the carboxylate group and the azobenzene ring. Thus, in the [Zn_2_L(μ-azo-OH)]^+^ cation the carboxylato group is twisted out of the azobenzene plane by an angle of 38.0°. In the neutral [Zn_2_L(μ-azo-O)] complex this angle is only 5.8°. Note that the azobenzene co-ligands are not planar. They are twisted by 8–10° about the C–N=N–C linkages, as in other structures. The Zn–carboxylato bond lengths reveal no anomalies and are similar to those in [Zn_2_L(μ-OAc)]^+^. There are no π–π stacking interactions between the azobenzene moieties. However, the [Zn_2_L(μ-azo-OH)]^+^ and [Zn_2_L(μ-azo-O)] complexes are connected by a OH···O hydrogen bond of length 2.46 Å (O3a···O3b, not shown in Figure 4).

**[Zn_2_L(μ-azo-NMe_2_)]ClO_4_·1.5MeCN (5·1.5MeCN):** Crystals of [Zn_2_L(μ-azo-NMe_2_)]ClO_4_·1.5MeCN (**5**·1.5MeCN) grown by recrystallization from MeCN are triclinic, space group *P*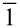. The asymmetric unit comprises a [Zn_2_L(μ-azo-NMe_2_)]^+^ cation, a ClO_4_^–^ anion, and MeCN solvate molecules. The [Zn_2_L]^2+^ unit is isostructural with **3'**. The co-ligand binds again via its carboxylato function in a μ_1,3_-bridging mode to give a Zn···Zn distance of 3.440 Å, which is typical for carboxylato-bridged Zn complexes supported by the macrocyclic N_6_S_2_ donor ligand. The average Zn–S, Zn–N and Zn–O distances are at 2.536 Å, 2.305 Å, and 2.049 Å, respectively. These values compare well with those in **3'** and other [Zn_2_L(μ-carboxylato)]^+^ complexes. The [Zn_2_L(μ-azo-NMe_2_)]^+^ complexes in **5** assemble in pairs (Figure 5) most likely via π···π stacking interactions, as manifested by the distance of 3.34 Å between the planes through the azobenzene moieties.

**[Cd_2_L(μ-Azo-NMe_2_)]ClO_4_·0.5MeOH (6·0.5MeOH):** Crystals of the title compound were grown from MeOH. The asymmetric unit comprises a [Cd_2_L(μ-azo-NMe_2_)]^+^ cation (with the carboxylate co-ligand disordered over two positions), a ClO_4_^−^ anion, and MeOH solvate molecules. The [Cd_2_L]^2+^ unit is isostructural with that in [Cd_2_L(μ-OAc)]^+^ and the compounds above, with the typical bowl-shaped structure of the [Cd_2_L]^2+^ fragment and a bridging carboxylate function (Figure 6). The average Cd–S, Cd–N and Cd–O distances at 2.663 Å, 2.428 Å, and 2.258 Å are longer than in **3'**, as one might expect from the larger ionic radius of Cd^2+^. The Cd···Cd distance is at 3.399 Å. Virtually the same values are observed in [Cd_2_L(μ-OAc)]^+^ [10]. As in **5** π–π stacking of the azo-carboxylato co-ligands occurs (Figure 7). The shortest distance between two carbon atoms of adjacent benzene rings is at 3.41 Å.

**[Ni_2_L(μ-Azo-NMe_2_)]ClO_4_·xEtOH (7·ClO_4_·*x*EtOH; *x* ≈ 4):** Crystals of [Ni_2_L(μ-azo-NMe_2_)]ClO_4_^.^*x*EtOH obtained from a mixed ethanol/acetonitrile solvent system are triclinic, space group *P*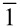. The asymmetric unit comprises two crystallographically independent [Ni_2_L(μ-azo-NMe_2_)]^+^ cations, two ClO_4_^–^ ions and 8 EtOH solvate molecules. The latter were found to be highly disordered and were therefore removed by the SQUEEZE procedure implemented in PLATON. Removing the EtOH molecules led to a total solvent accessible void of 1500 Å^3^, in good agreement with the space needed by ca. eight ethanol solvate molecules. The two [Ni_2_L(μ-azo-NMe_2_)]^+^ cations are structurally very similar as illustrated in Figure 8. In cation A, the co-ligand is nearly flat and coplanar with the Ni_2_ carboxylato plane. In cation B, the benzene rings are tilted about the azo group and the Ni_2_carboxlyato plane by angles of ≈32.3° and 37.5°, respectively. Interestingly, no π–π stacking occurs in this structure.

**Figure 9:**
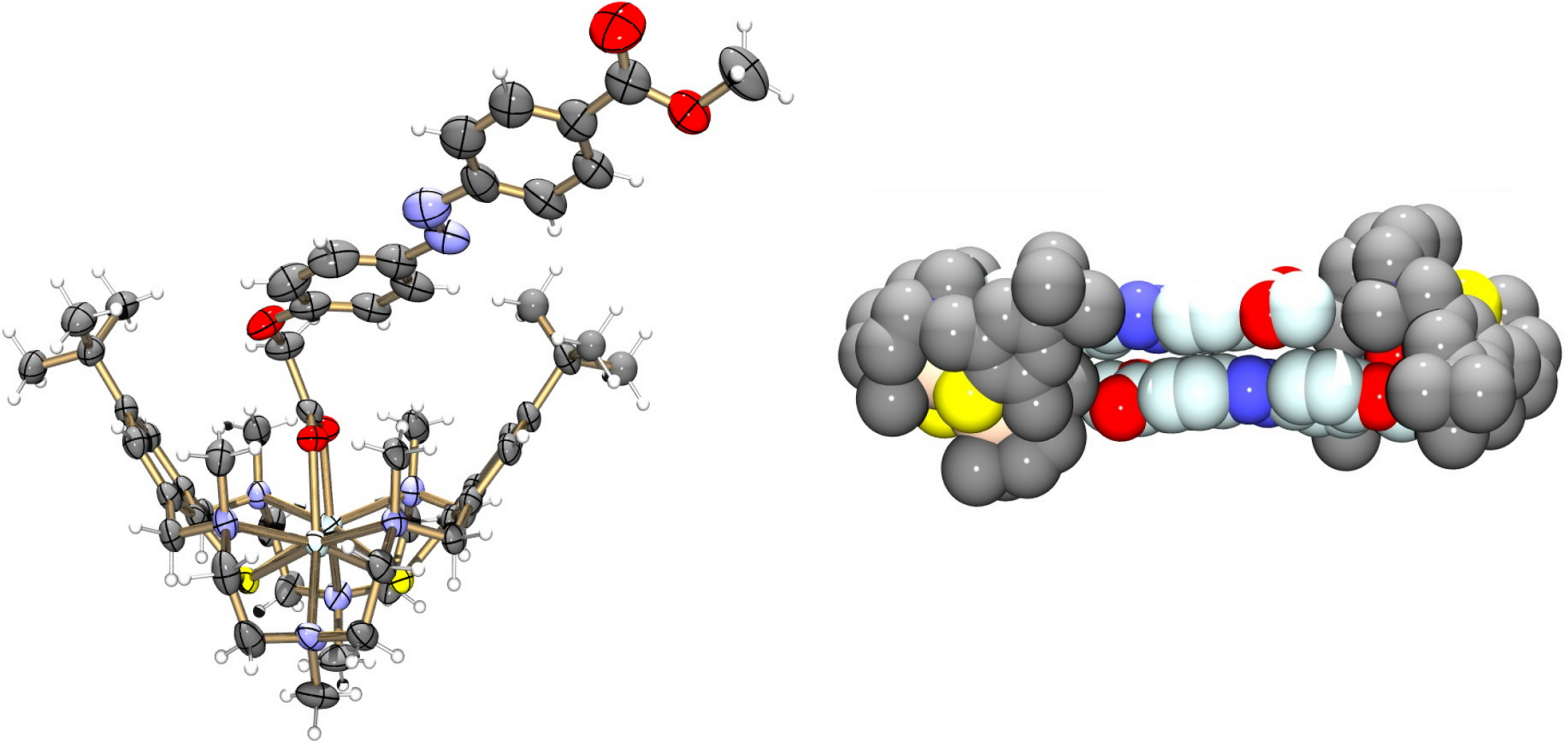
Left: ORTEP representation of the molecular structure of the [Cd_2_L(μ*-*azo-CO_2_Me)]^+^ cation in crystals of **8**·MeCN. Right: Space filling representation of the packing of two [Cd_2_L(μ-azo-CO_2_Me)]^+^ cations in crystals of **8**·MeCN*.* H atoms, BPh_4_^−^ anions and solvate molecules are omitted for clarity.

**[Cd_2_L(μ-azo-CO_2_Me)]BPh_4_^.^MeCN (8·MeCN):** The crystal structure determination of **8**·MeCN confirms that azo-CO_2_Me is also attached in the bridging mode. Figure 9 shows an ORTEP representation of the structure of the [Cd_2_L(μ-azo-CO_2_Me)]^+^ cation in **8**. The [Cd_2_L]^2+^ fragment is isostructural with that in **6**, but the co-ligand protrudes laterally out of the binding pocket of the [Cd_2_L]^2+^ fragment, most likely due to steric constraints exerted by the surrounding NMe_2_ groups. We have observed similar effects in a stearato bridged complex, where the surrounding alkyl groups dictate the coordination mode of the carboxylato ligand [13]. The average Cd–S, Cd–N and Cd–O distances at 2.670, 2.429, and 2.258 Å, respectively, are all very similar to those in **6**. The Cd···Cd distance is at 3.405 Å. Again, π–π stacking of the azo-carboxylato co-ligand occurs, the shortest distance between the two planes through the co-ligands amounts to 3.45 Å.

### Magnetic properties of nickel complexes

**Figure 10:**
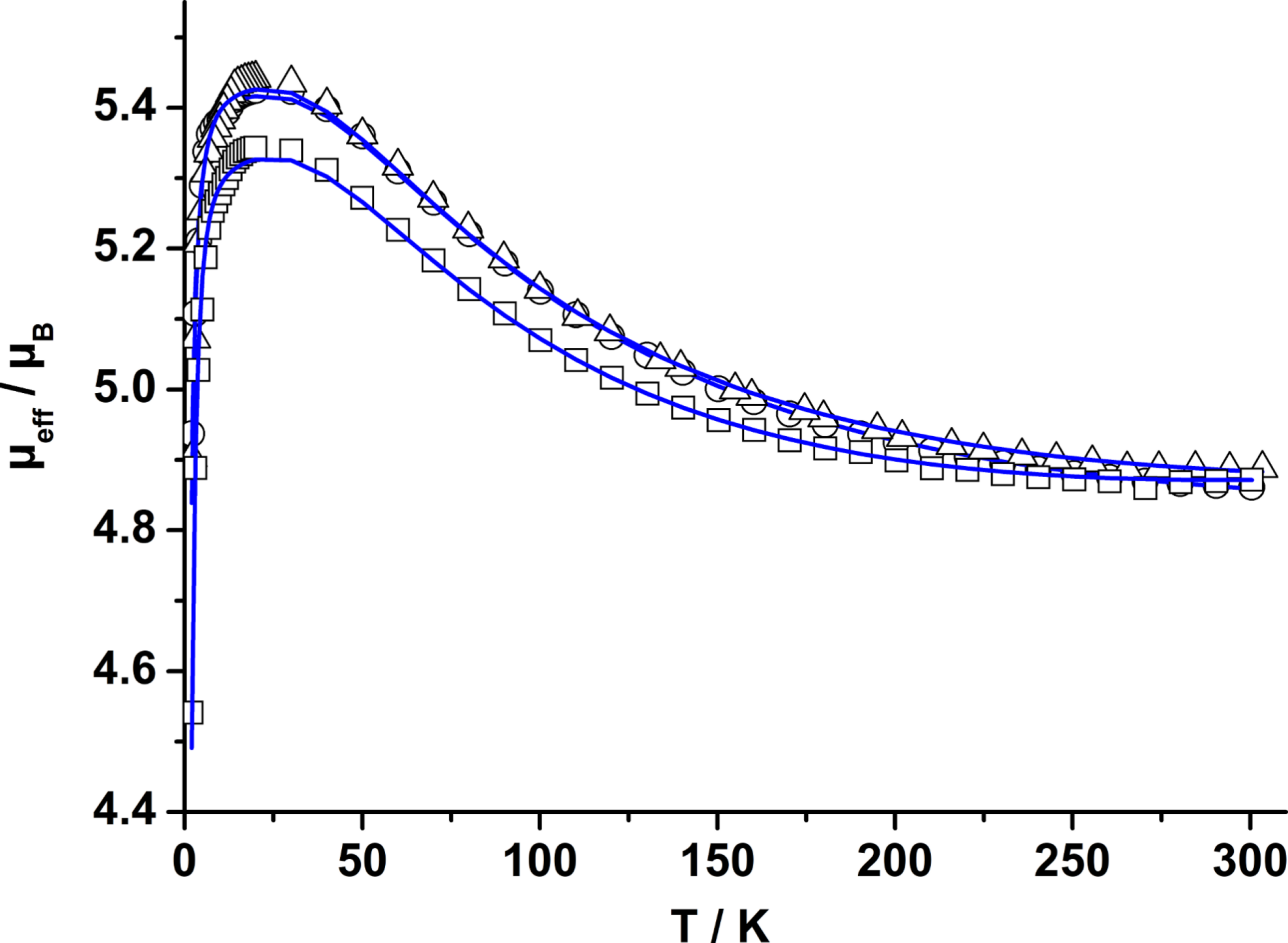
Plots of the effective magnetic moment μ_eff_ for **2** (open circles), **4** (open squares), and **7** (open triangles) at H = 0.5 T (**2**, **7**) and 1.0 T (**4**). The solid lines represent the best theoretical fit to Equation 1 (see text). Experimental and calculated values are provided as Supporting Information File 1.

The paramagnetic nickel complexes **2**, **4**, and **7** were further investigated by temperature dependent magnetic susceptibility measurements between 2 and 300 K in an applied external field of 0.5 T (or 1 T) by using a MPMS 7XL SQUID magnetometer (Quantum Design). Figure 10 shows the susceptibility data in the form of μ_eff_ versus *T* plots for the three complexes.

For complex **2**, the effective magnetic moment increases from 4.86 μ_B_ (per dinuclear complex) at 300 K to a maximum value of 5.42 μ_B_ at 20 K, and then drops to 4.93 μ_B_ at 2 K. The complexes **4** and **7** behave in a similar fashion. At 300 K, μ_eff_ is at ≈4.87 per dinuclear complex which increases to maximum values of 5.34 μ_B_ (**4**) and 5.44 μ_B_ (**7**) at 20 K. On further lowering of the temperature, these values decrease again to 4.54 μ_B_ and 4.90 μ_B_ at 2 K. This behavior implies that the electron spins on the two Ni^II^ (*S* = 1) ions are coupled by an intramolecular ferromagnetic exchange interaction. This leads to an *S*_total_ = 2 ground state, in agreement with other carboxylato-bridged compounds supported by L^2−^ [14–16]. The ferromagnetic exchange interaction can be rationalized in terms of the Goodenough–Kanamori rules for superexchange [17], and recent DFT calculations, which revealed that a dominant ferromagnetic exchange interaction is propagated via the thiolato bridges [13].

The magnitude of the exchange interactions was determined by least-squares fitting of the experimental magnetic susceptibility data to the appropriate spin Hamiltonian (Equation 1) [18–20] including the isotropic HDvV (Heisenberg Dirac van Vleck) exchange interaction, the single-ion zero-field splitting of Ni(II) and the single-ion Zeeman interactions using a full-matrix diagonalization approach [21]. The experimental susceptibility data were fitted to Equation 1 over the temperature range 2–300 K, assuming identical *D* and *g* values for the two Ni(II) ions in each one of the complexes, but it should be mentioned that temperature-dependent magnetic susceptibility measurements do not allow a concise determination of the magnitude and sign of *D* [22].

[1] $$\hat{H}=\;-2\;J{\hat{S}}_{1}{\hat{S}}_{2}+\overset{2}{\underset{i=1}{\sum }}\left(D_{i}\;\left({\hat{S}}_{zi}^{2}-\frac{1}{3}{\hat{S}}_{i}\left({\hat{S}}_{i}+1\right)\right)+\;g_{i}\mu_{\text{B}}S_{i\tau }B_{\tau }\right)\;(\tau \;=\;x,\;y,\;z)$$

By taking into account zero-field splitting and temperature-independent paramagnetism (TIP), reasonable fits of the experimental data were possible, yielding *J* = +22.6 cm^−1^, *g* = 2.21 and *D* = |4.18| cm^−1^ for **2**. Fitting the experimental data of **4** and **7** led to very similar values, namely *J* = +21.79 cm^–1^, *g* = 2.17, *D* = |3.64| cm^–1^ and *J* = +21.70 cm^–1^, *g* = 2.22 and *D* = |4.67| cm^–1^, respectively. In each case the low-temperature fit was significantly improved by the inclusion of the *D* parameter, but as stated above, the *D* values should be taken rather indicative than definite [23]. The magnetic parameters for the azo-carboxylate complexes are in good agreement with those of nickel(II)-carboxylate complexes supported by L^2−^. For [Ni_2_L(μ-O_2_C(CH_2_)_10_SH)](ClO_4_), for example, values of *J* = +23.0 cm^−1^, *g* = 2.20 and *D* = |2.59| cm^−1^ were determined [13].

### UV–vis spectroscopy

**Figure 11:**
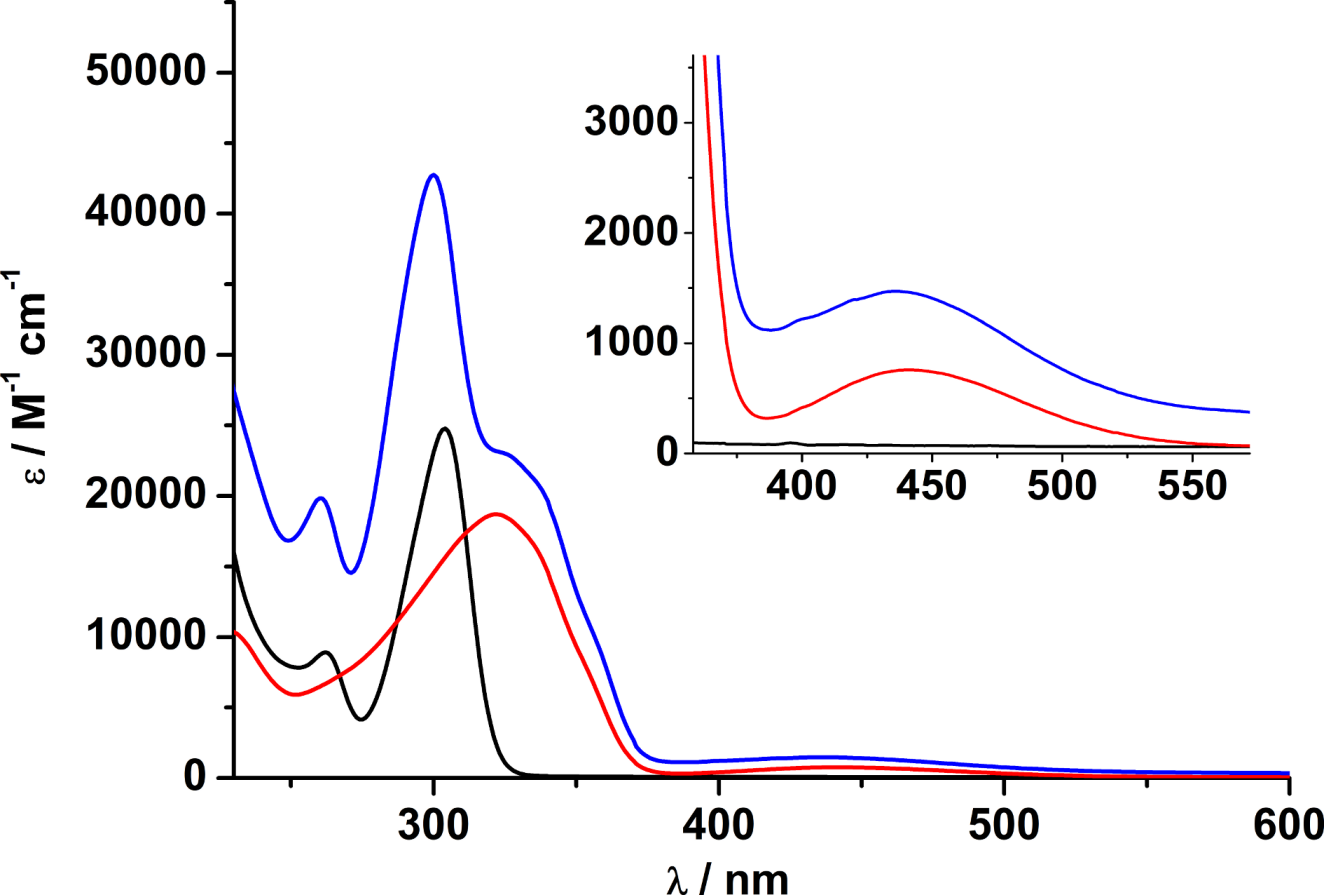
UV–vis spectra of Hazo-H (red line), [Cd_2_L(μ-Cl)](ClO_4_) (black line) and [Cd_2_L(μ-azo-H)]ClO_4_ (**1**, blue line) in acetonitrile. Concentration of solutions: 10^−4^ M.

All new compounds were further investigated by electronic absorption spectroscopy. The electronic absorption spectra were recorded in acetonitrile (**1**, **2** and **5**–**9**) or dimethyl sulfoxide (**3**, **4**) solution in the 190 to 1200 nm spectral range. Figure 11 shows a representative set of spectra recorded for Hazo-H and the corresponding Cd(II) complex [Cd_2_(L)(μ-azo-H)]^+^ (**1**). The spectrum of the chlorido-bridged complex [Cd_2_L(μ-Cl)]^+^ is also shown for comparison. Table 3 lists the corresponding data. A comparison reveals that the electronic absorption spectrum of [Cd_2_L(μ-azo-H)]^+^ can (at least in the 260–450 nm range) in first order be traced to a superposition of the absorptions of the Cd-bound amino-thiophenolato ligand (absorbing at 260 and 300 nm) and the azobenzene co-ligand (absorbing at 325 and 441 nm). Note, that the band at 322 nm for free Hazo-H appears as a shoulder around 325 nm in **1**. The slight red-shift may be a consequence of deprotonation and coordination to the Cd^2+^ ions. The spectrum of the deprotonated azoH^–^ ion, for comparison, absorbs at 329 nm. The absorption bands above 310 nm can thus be attributed to the π–π* and n–π* transitions of the *trans*-configured azobenzene chromophore, consistent with literature reports [24–25]. The absorptions below 310 nm (λ = 260 and 300 nm) are attributable to the π–π* transitions within the thiophenolate units of the [Cd_2_L]^2+^ fragment, which may be further differentiated as *p*- and α-bands of the parent benzene chromophore utilizing Clar’s notation [26], assuming that the electron-donating alkyl and thiol substituents exert a bathochromic effect.

**Table 3:** Selected UV–vis data for compounds **1–9**.^a^

Complex/co-ligand	λ_max_ [nm] (ε [M^–1^ cm^–1^])^a^

[Cd_2_L(µ-Cl)]^+^,^b^	194 (63477), 262 (8926), 304 (24784)
[Ni_2_L(µ-Cl)]^+^,^b^	194 (51524), 265 (13994), 304 (12283), 645 (48), 920 (65), 999 (85)
[Zn_2_L(µ-OAc)]^+^,^b^	192 (55860), 208 sh (41434), 261 (13740), 288 (24464)
Hazo-H^b^	190 (45246), 228 (10477), 322 (18707), 441 (757)
Hazo-OH^c^	194 (432), 243 (209), 352 (371)
Hazo-NMe_2_^b^	193 (44362), 253 sh (13356), 280 (12545), 311 (11315), 436 (15104)
Hazo-CO_2_Me^b^	190 (40583), 232 (9820), 255 (9915), 352 (16597), 439 (1380)
**1** [Cd_2_L(µ-azo-H)]^+^,^b^	192 (104317), 260 (19844), 300 (42752), 325 sh (23011), 441 (882)
**2** [Ni_2_L(µ-azo-H)]^+^,^b^	195 (88412), 276 sh (24155), 312 (33230), 328 (34994), 439 (1180), 647 (39), 1117 (66)
**3** [Zn_2_L(µ-azo-OH)]^+^,^c^	259 (24979), 292 (24378), 366 (28535), 450 (1970)
**4** [Ni_2_L(µ-azo-OH)]^+^,^c^	258 (27612), 311 (20738), 341 (29144), 364 (31733), 454 (2099), 645 (115), 1113 (76)
**5** [Zn_2_L(µ-azo-NMe_2_)]^+^,^b^	195 (83650), 261 (27080), 285 (31050), 382 (11790), 434 (16000)
**6** [Cd_2_L(µ-azo-NMe_2_)]^+^,^b^	193 (129340), 261 (30525), 298 (38705), 381 (12432), 434 (16292)
**7** [Ni_2_L(µ-azo-NMe_2_)]^+^,^b^	194 (114417), 267 (37868), 301 sh (24544), 328 sh (20921), 380 (16567), 431 (19801), 650 (221), 1112 (186)
**8** [Cd_2_L(µ-azo-CO_2_Me)]^+^,^b^	193 (113128), 261 (29102), 300 (33143), 370 (6018), 447 sh (2949)
**9** [Ni_2_L(µ-azo-CO_2_Me)]^+^,^b^	194 (113002), 261 (35911), 308 (28168), 334 (32443), 360 sh (29137), 448 (3186), 645 sh (470), 1113 (153)

^a^The complexes were isolated as ClO_4_^−^ salts. ^b^Solvent: CH_3_CN or ^c^DMSO. Concentration of solutions: 10^−4^ M.

**Figure 12:**
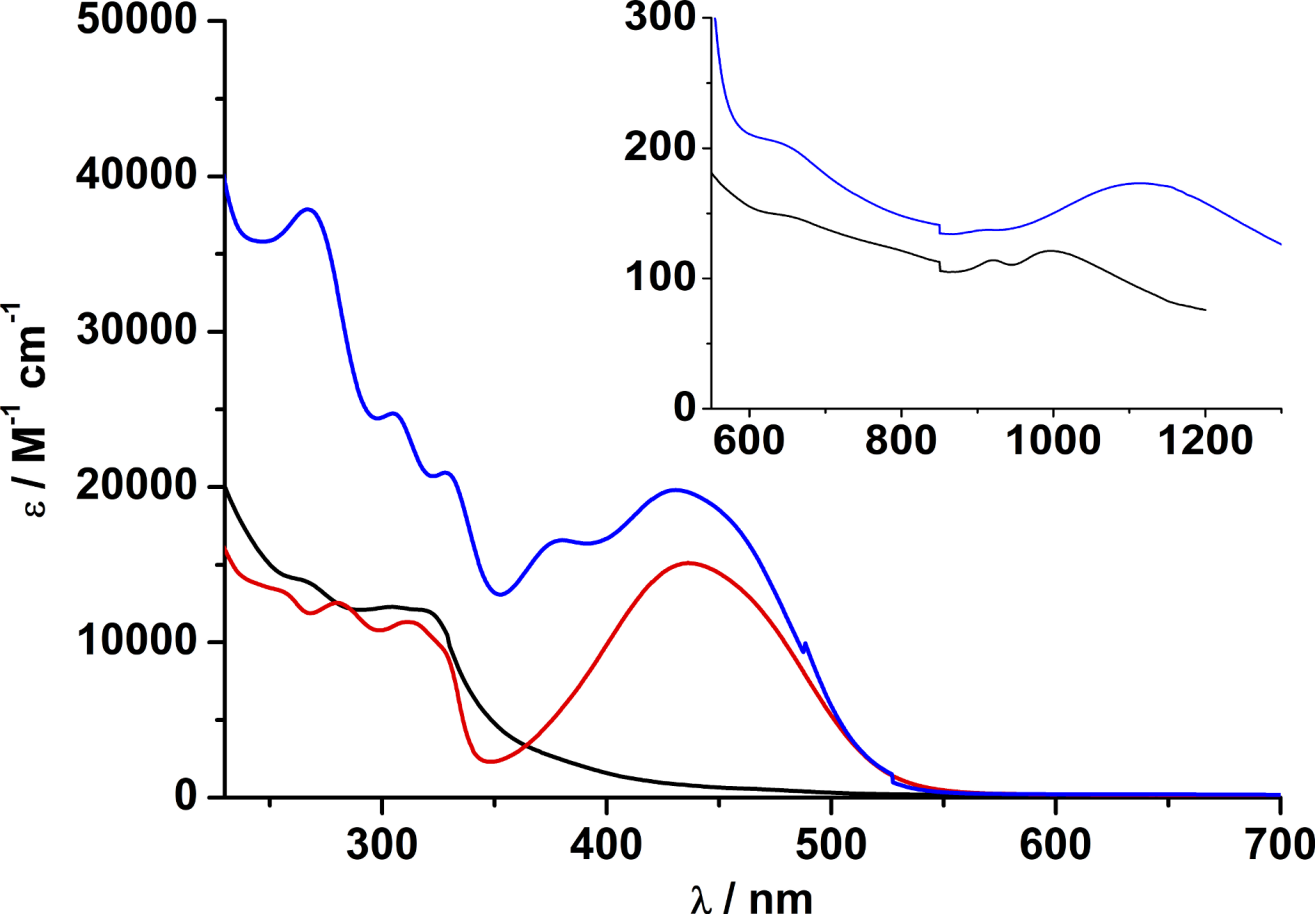
UV–vis spectra of Hazo-NMe_2_ (red line), [Ni_2_L(μ-Cl)](ClO_4_) (black line) and [Ni_2_L(μ-azo-NMe_2_)]ClO_4_ (**7**, blue line) in acetonitrile at 230–700 nm and 550–1300 nm (inset). Concentration of solutions: 10^−4^ M.

The spectral properties of the nickel complexes differ from those of the zinc and cadmium counterparts in that they exhibit two additional Laporte-forbidden but spin-allowed d–d transitions around 645 and 1120 nm, typical for carboxylato-bridged Ni_2_ complexes supported by H_2_L (Figure 12) [5]. They are assigned as ^3^A_2g_(F)→ T_1g_(F) (ν_2_) and ^3^A_2g_(F) → ^3^T_1g_(P) (ν_1_) of an octahedral nickel(II) (S = 1) ion (in pure *O_h_* symmetry) [5]. The third spin-allowed ^3^A_2g_(F) → ^3^T_2g_(F) (ν_3_) transition is expected around 440 nm, but is obscured by the stronger RS → Ni^2+^ charge transfer and π→π* transitions in this region.

**Figure 13:**
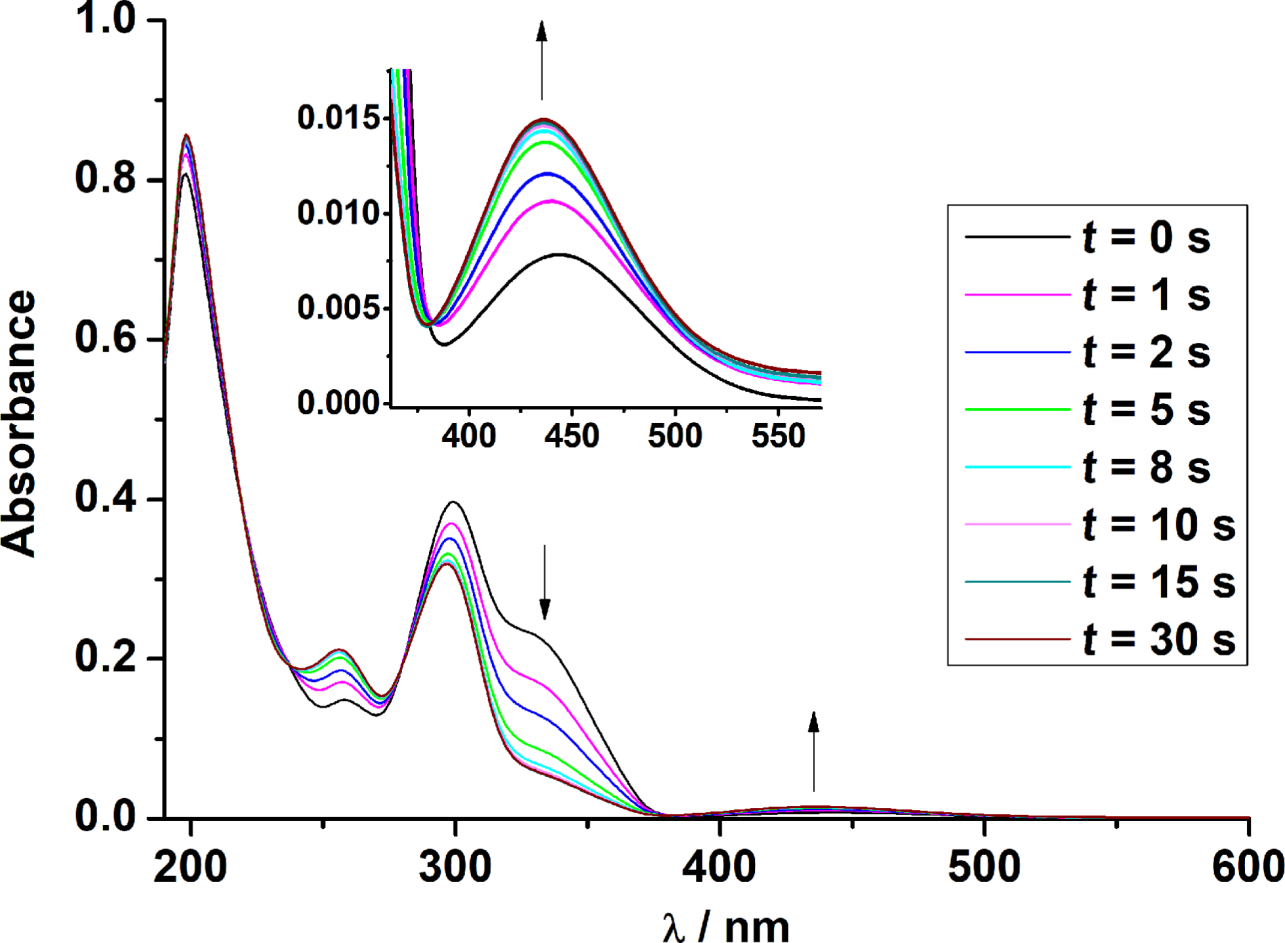
UV–vis spectra of solutions of [Cd_2_L(μ-azo-H)]ClO_4_ (**1**) in acetonitrile irradiated with a UV LED lamp (365 nm) for 1 to 30 s after irradiation. Concentration of solutions: 10^−5^ M.

We carried out orienting irradiation experiments with regard to a possible *trans* to *cis* photoisomerization of the bound azobenzene-carboxylate co-ligands. The diamagnetic Cd_2_ complex [Cd_2_L(μ-azoH)]ClO_4_ (**1**) was selected for these studies. Note that this complex (with the azobenzene moiety in the *trans* form) is distinguished by a strong absorption band (shoulder) around 325 nm (ε = 23000 M^–1^cm^–1^) that originates from the π→π* transition in *trans* azo-H. The much weaker n→π* band appears in the visible at 450 nm. Dilute anhydrous acetonitrile solutions of complex **1** were placed in a standard quartz cuvette and irradiated at 365 nm for varying amounts of time (1 to 30 s) with a 14 W/cm^2^ UV LED lamp and thereafter immediately placed in the chamber of a UV–vis spectrometer. Figure 13 shows the corresponding UV–vis spectra, which were recorded directly after sample preparation.

**Scheme 2:**
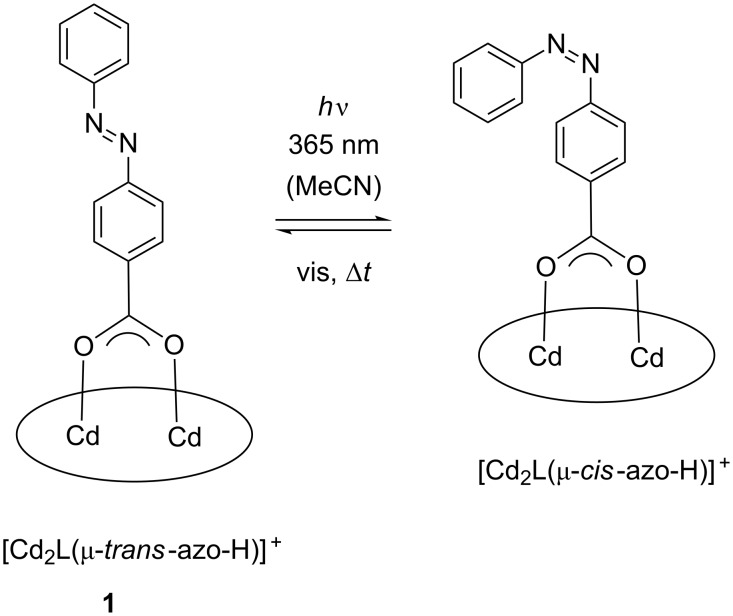
*Cis/trans* isomerization process of the bound azo-carboxylato co-ligand in [Cd_2_L(μ-azo-H)]ClO_4_ (**1**) in acetonitrile mediated by irradiation with 365 nm UV light. The amino-thiophenolato ligand is shown as an ellipse for clarity.

As can be seen, the intensity of the π→π* band at 325 nm for the *trans*-azobenzene carboxylate decreases with increasing irradiation time, while the intensity of the *n*→π* band increases with increasing irradiation time. A blue shift of the *n*–π* band by ≈10 nm is evident. The π→π* transitions for the thiophenolate group at 260 and 300 nm are also affected, being blue-shifted to 256 and 296 nm, respectively, and significantly reduced (≈260 nm) or increased (≈300 nm) in intensity. Isosbestic points at 220, 240, 280 and 380 nm are also clearly discernible indicative of the presence of a single equilibrium. On the basis of these data, we conclude that the *trans*-form of the bound co-ligand converts to its *cis*-form as indicated in Scheme 2. Further studies show, that the meta-stable *cis*-form relaxes back thermally to the more stable *trans* from (Figures S50 and S51 in Supporting Information File 1). Thus, the *cis–trans* isomerization appears to be not constrained by the [Cd_2_L]^2+^ fragment, as revealed by the similar half-lives (τ_1/2_ *cis–trans* free deprotonated azobenzene carboxylate: ≈80 h; τ_1/2_ *cis–trans* Cd complex ≈73 h. In view of the fact that the azobenzene carboxylate is only partially buried in the binding pocket of the [Cd_2_L]^2+^ complex this is not surprising.

## Conclusion

A series of dinuclear macrocyclic [M_2_L(μ-L')]^+^ complexes co-ligated by various azobenzene-carboxylates has been synthesized and characterized in solution and solid state. The diamagnetic Cd(II) and Zn(II) and the paramagnetic Ni(II) complexes were found to be isostructural, with the co-ligands bound via bridging carboxylate functions, a bowl-shaped structure of the supporting [M_2_L]^2+^ entity, and *trans*-configured azo groups. NMR and UV–vis studies clearly show that the complexes retain their mixed-ligand nature in the solution state. Irradiation of the Cd complex **1** with a LED UV lamp leads to a *trans*-to-*cis* isomerization which appears to proceed within the coordination sphere of the [Cd_2_L]^2+^ fragment. The switch from the *trans* to the *cis* form induces significant change of the π–π transitions of the supporting N_6_S_2_ macrocycle, which may be indicative of some increased π–π- (or charge transfer) interactions between the aromatic rings of the electron rich amino thiophenolato macrocycle and the electron poor azobenzene-carboxylato ligands. There are little, if any, differences in the time-scales for thermal relaxation of the free and Cd-bound azobenzene-carboxylate systems, suggesting that the cavity of [Cd_2_L]^2+^ does not sterically constrain the photoisomerization process.

## Experimental

Unless otherwise noted all preparations were carried out under a protective atmosphere of nitrogen by using standard Schlenk techniques [27]. Compounds H_2_L [28], [Ni_2_L(μ-Cl)]ClO_4_ [29], [Cd_2_L(μ-Cl)]ClO_4_, and *p*-hydroxyazobenzene-4-carboxylic acid [30] and *p*'-carbomethoxy-azobenzene-*p*-oxymethylcarboxylic acid [31–33] were prepared as described in the literature. All other reagents were purchased from commercial vendors and used without further purification. Melting points were determined in open glass capillaries and are uncorrected. The IR spectra were recorded as KBr disks using a Bruker Tensor 27 FTIR spectrometer. UV–vis spectra were recorded on a Jasco V-670 UV–vis/near-IR spectrophotometer. Elemental analyses were carried out with a Vario EL – elemental analyzer. NMR spectra were recorded on a Bruker Fourier 300 spectrometer or Avance DRX 400 at 298 K. ^1^H and ^13^C chemical shifts refer to solvent signals. ESIMS spectra were recorded on a Bruker Daltronics Esquire 3000 Plus spectrometer. Temperature-dependent magnetic susceptibility measurements on powdered solid samples were carried out using a MPMS 7XL Squid magnetometer (Quantum Design) over a temperature range of 2**–**300 K at an applied magnetic field of 0.5 and 1.0 Tesla. The observed susceptibility data were corrected for underlying diamagnetism. The synthesis of the complexes are shown in Supporting Information File 1.

**X-ray crystallography.** Single crystals of **3'**, **5**–**8** suitable for X-ray crystallography were selected and mounted on the tip of a glass fiber. The data sets were collected at 213(2) K or 200 K (**7**) using a STOE IPDS-2T diffractometer equipped with graphite monochromated Mo Kα radiation (0.71073 Å). Table S7 in Supporting Information File 1 lists selected crystallographic data. The intensity data were processed with the program STOE X-AREA [34] Structures were solved by direct methods [35] and refined by full-matrix least-squares on the basis of all data against F^2^ using SHELXL-97 [36]. PLATON was used to search for higher symmetry [37]. H atoms were placed in calculated positions and treated isotropically using the 1.2-fold *U*_iso_ value of the parent atom except methyl protons, which were assigned the 1.5-fold *U*_iso_ value of the parent C atoms. Unless otherwise noted, all non-hydrogen atoms were refined anisotropically. ORTEP-3 was used for the artwork of the structures [38]. All non-hydrogen atoms were refined anisotropically.

In the crystal structure of **3'**·4MeCN·3H_2_O one MeCN molecule was found to be disordered over two positions (at half occupancy). It was not possible to locate H atoms for the H_2_O solvate molecules, and so no H_2_O hydrogen atoms were included in this structure. In the crystal structures of **5** and **6** the co-ligands were found to be disordered over two positions. This disorder could be successfully modelled by using appropriate DFIX, DANG and AFIX instructions implemented in SHELXL. The site occupancy factors of the two orientations were initially refined, but were fixed at 0.65 and 0.35 (or 0.50 and 0.50 for **3'**) in the final refinement cycle. The occupancy factor of a MeCN (for **5**) and a MeOH solvate molecule (for **6**) was set at 0.5 to keep the *U*_eq_ values reasonable. For **7**, additional solvent molecules occupy interstitial spaces that are generated by packing, and were found to be highly disordered. The routine SQUEEZE was applied to remove diffuse electron density [39].

CCDC-1892644 (**3'**), -1892646 (**5**) -1892647 (**6**), -1892648 (**8**) and -1892649 (**7**) contain the supplementary crystallographic data for this paper. These data can be obtained free of charge from The Cambridge Crystallographic Data Centre via http://www.ccdc.cam.ac.uk/data_request/cif.

**Irradiation experiments.** Irradiation experiments were performed on dry acetonitrile solutions of complex **1** and azo-H (concentration = 10^−5^ M) in a quartz cuvette at room temperature. An OmniCure LX500 system and a 14 W/cm^2^ UV LED lamp (365 nm) from Excelitas Technologies was used. After irradiation, the solutions were immediately placed in the chamber of the JASCO V-670 UV–vis/near-IR spectrophotometer for subsequent spectroscopic investigations.

## Supporting Information

File 1 Experimental and analytical data.
