# Supplementary material for: Coordination chemistry and photoswitching of dinuclear macrocyclic cadmium-, nickel-, and zinc complexes containing azobenzene carboxylato co-ligands
Source: Beilstein J Org Chem. 2019 Apr 3;15:840–51. doi: 10.3762/bjoc.15.81 (PMC6466696; doi:10.3762/bjoc.15.81)
Supplement: File 1 — Experimental and analytical data. [file Beilstein_J_Org_Chem-15-840-s001.pdf]

BEILSTEIN JOURNAL OF ORGANIC CHEMISTRY

Supporting Information

for

Coordination chemistry and photoswitching of dinuclear macrocyclic cadmium-, nickel-, and zinc complexes containing azobenzene carboxylato co-ligands

Jennifer Klose, Tobias Severin, Peter Hahn, Alexander Jeremies, Jens Bergmann, Daniel Fuhrmann, Jan Griebel, Bernd Abel and Berthold Kersting

Beilstein J. Org. Chem. **2019,** *15,* 840–851. doi:10.3762/bjoc.15.81

Experimental and analytical data

License and Terms: This is a supporting information file under the terms of the Creative Commons Attribution License (http://creativecommons.org/licenses/by/4.0). Please note that the reuse, redistribution and reproduction in particular requires that the authors and source are credited.
The license is subject to the *Beilstein Journal of Organic Chemistry* terms and conditions: (https://www.beilstein-journals.org/bjoc)

Table of content

1. Analytical data for compound **1**. S3

2. Analytical data for compound **2**. S7

3. Analytical data for compound **3**. S10

4. Analytical data for compound **4**. S14

5. Analytical data for compound **5**. S17

6. Analytical data for compound **6**. S20

7. Analytical data for compound **7**. S24

8. Analytical data for compound **8**. S27

9. Analytical data for compound **9**. S29

10. Selected NMR data S31

11. Selected metrical data for structurally characterized compounds S33

12. Crystallographic data for the complexes S35

13. Irradiation experiments S36

14. Experimental section S38

1. Analytical data for compound 1.

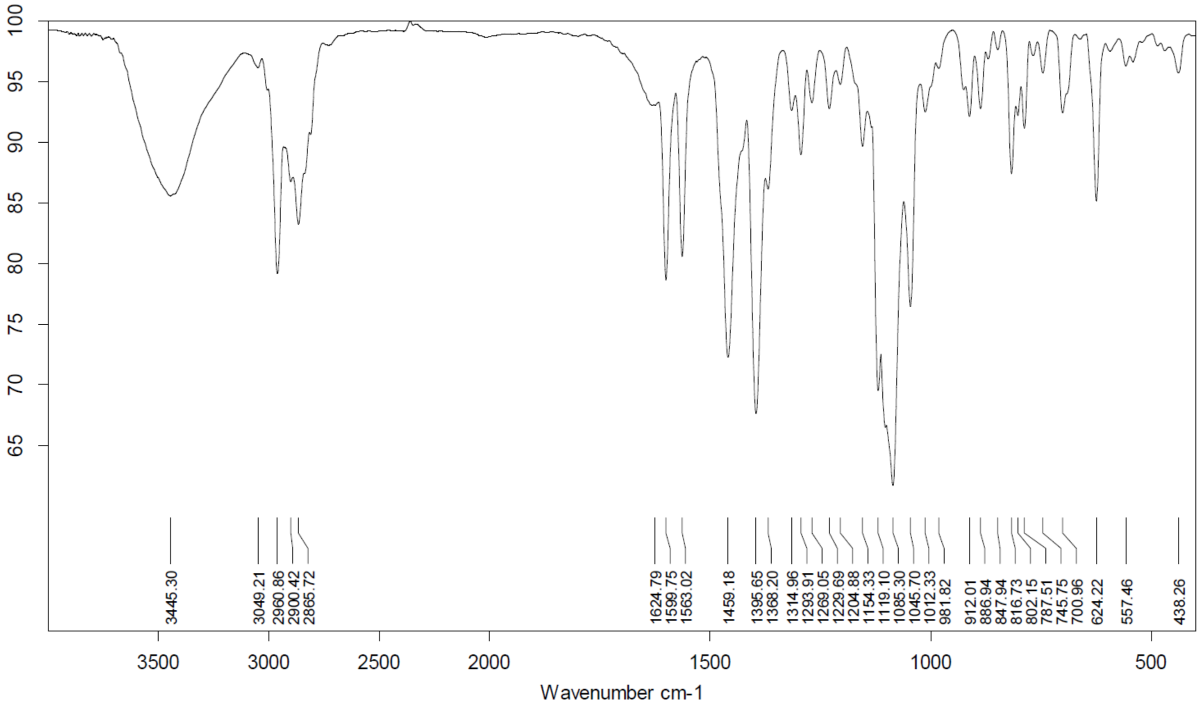

Figure S1. Infrared spectrum of **1**.

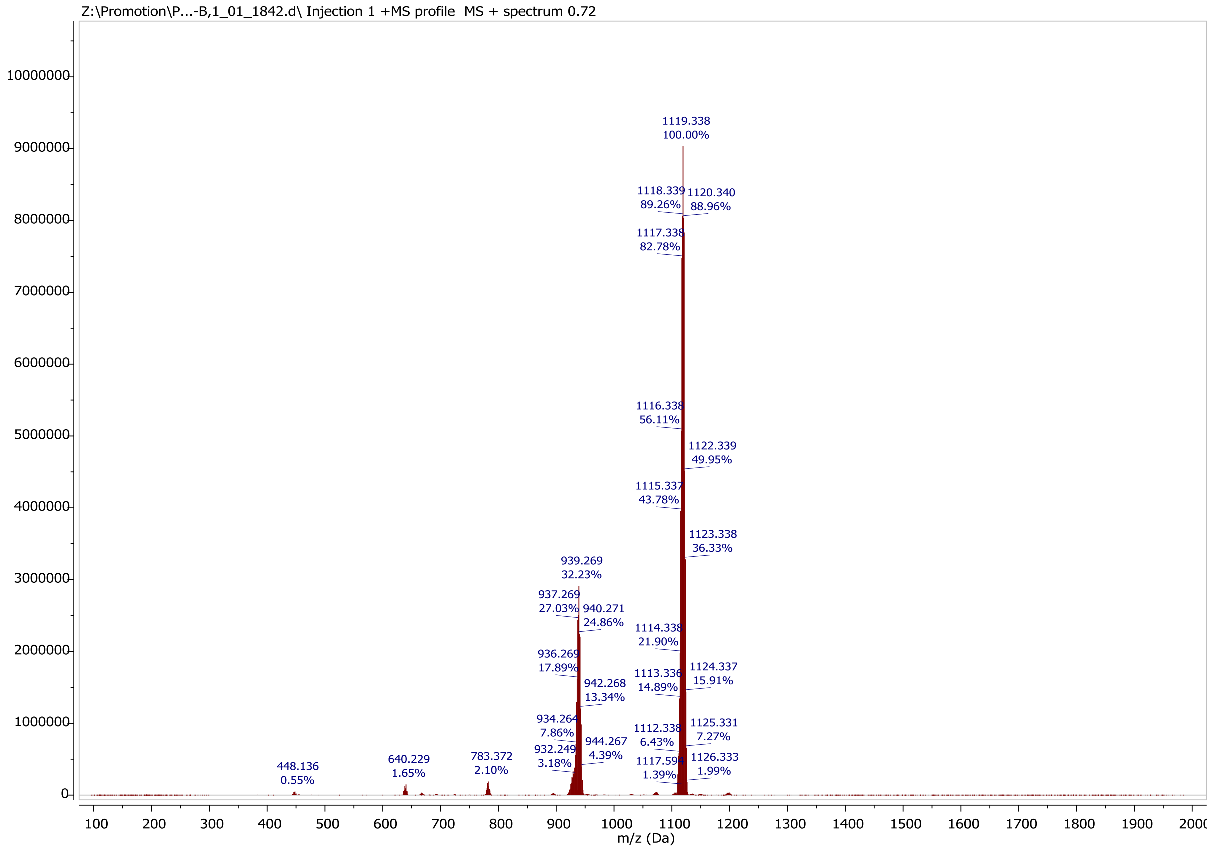

Figure S2. ESI mass spectrum of **1**.

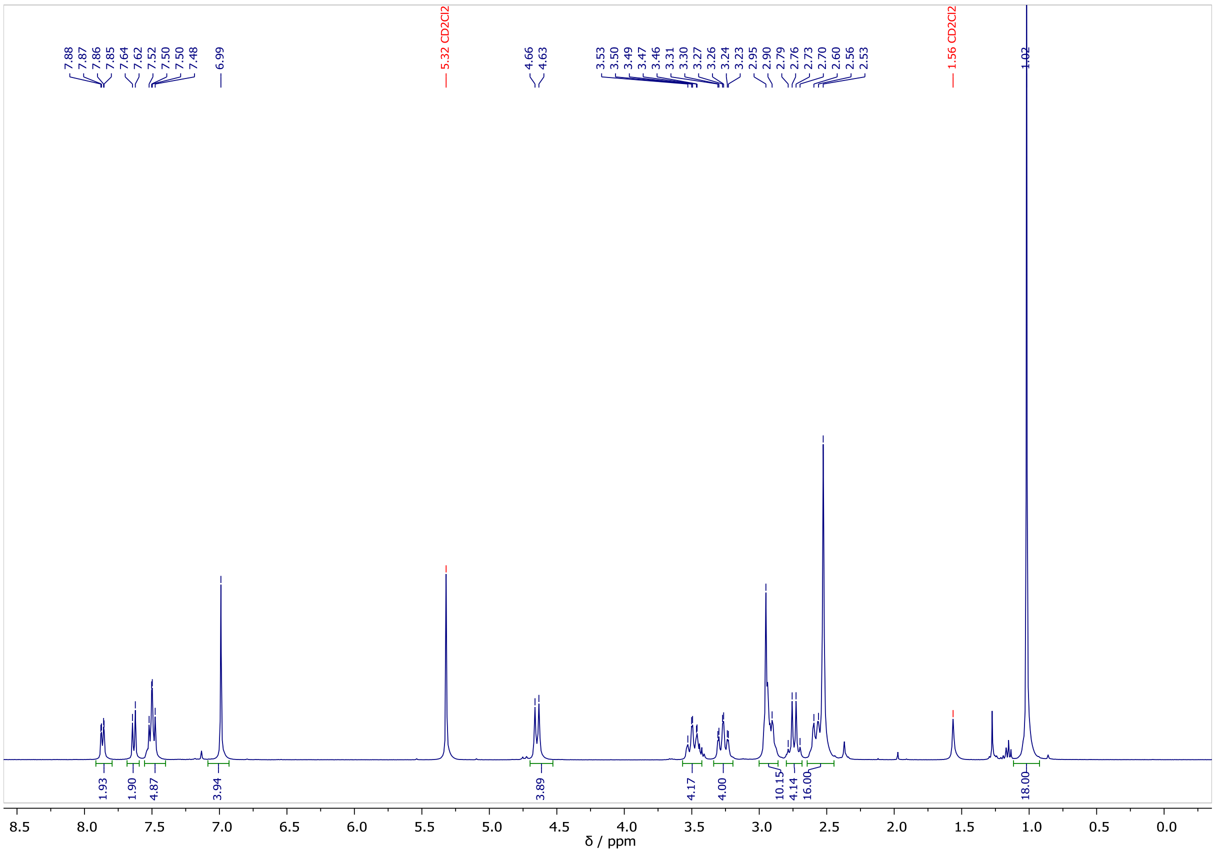

Figure S3. ^{1}H NMR spectrum of **1** in CD_2Cl_2 at ambient temperature.

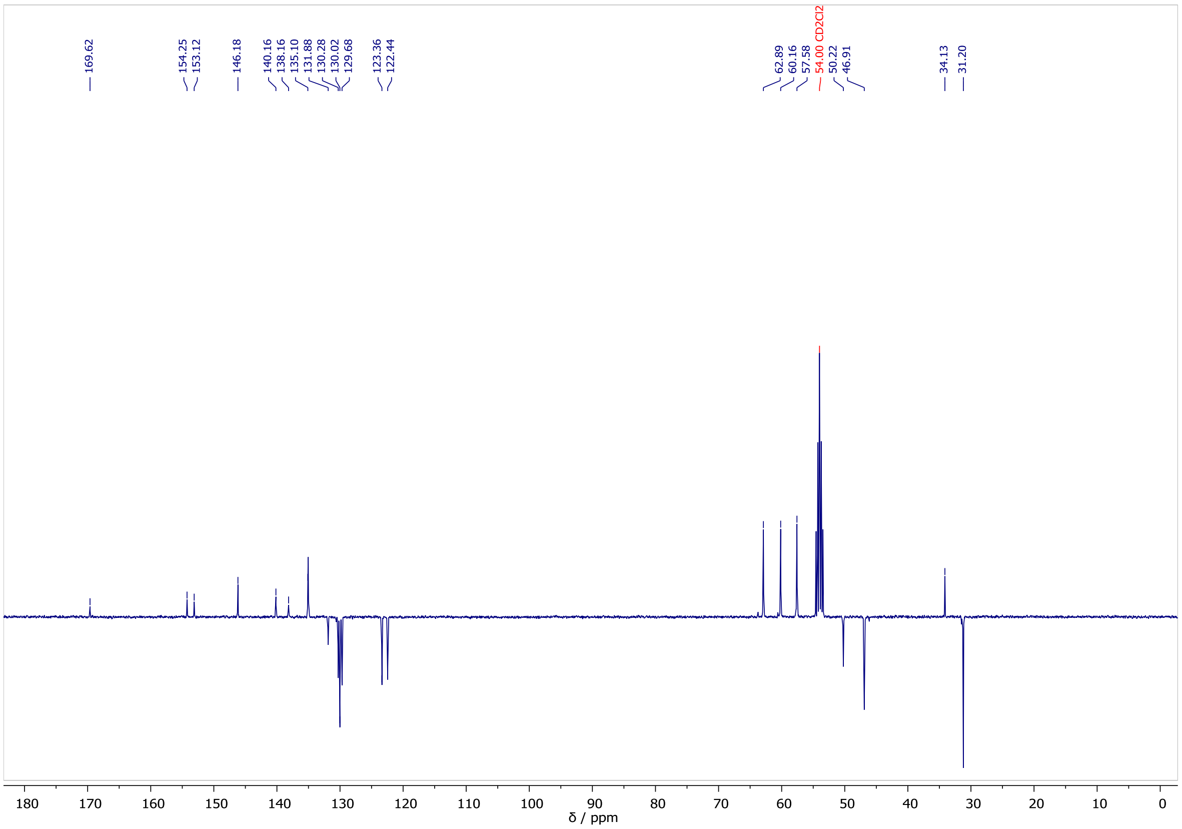

Figure S4. APT spectrum of **1** in CD_2Cl_2 at ambient temperature.

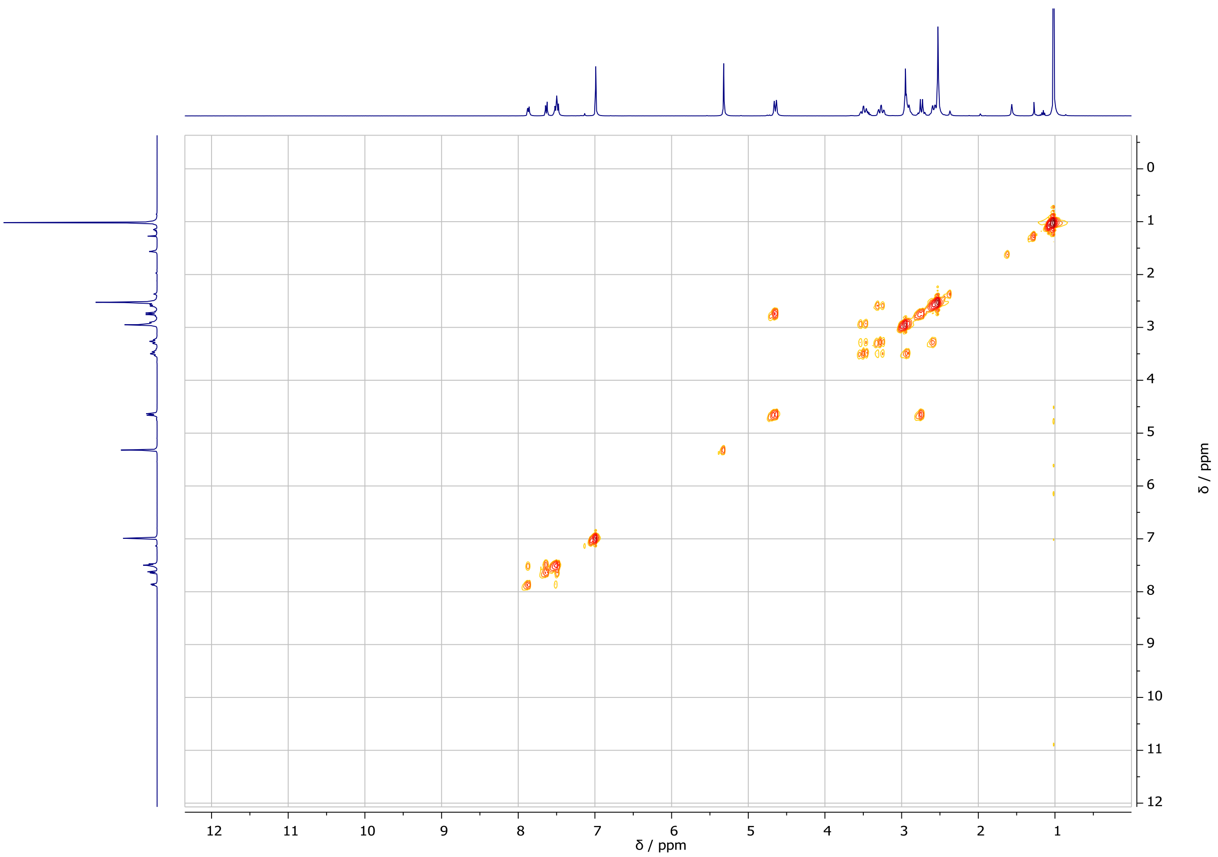

Figure S5. $^{1}H,^{1}H$ COSY spectrum of **1** in CD_2Cl_2 at ambient temperature.

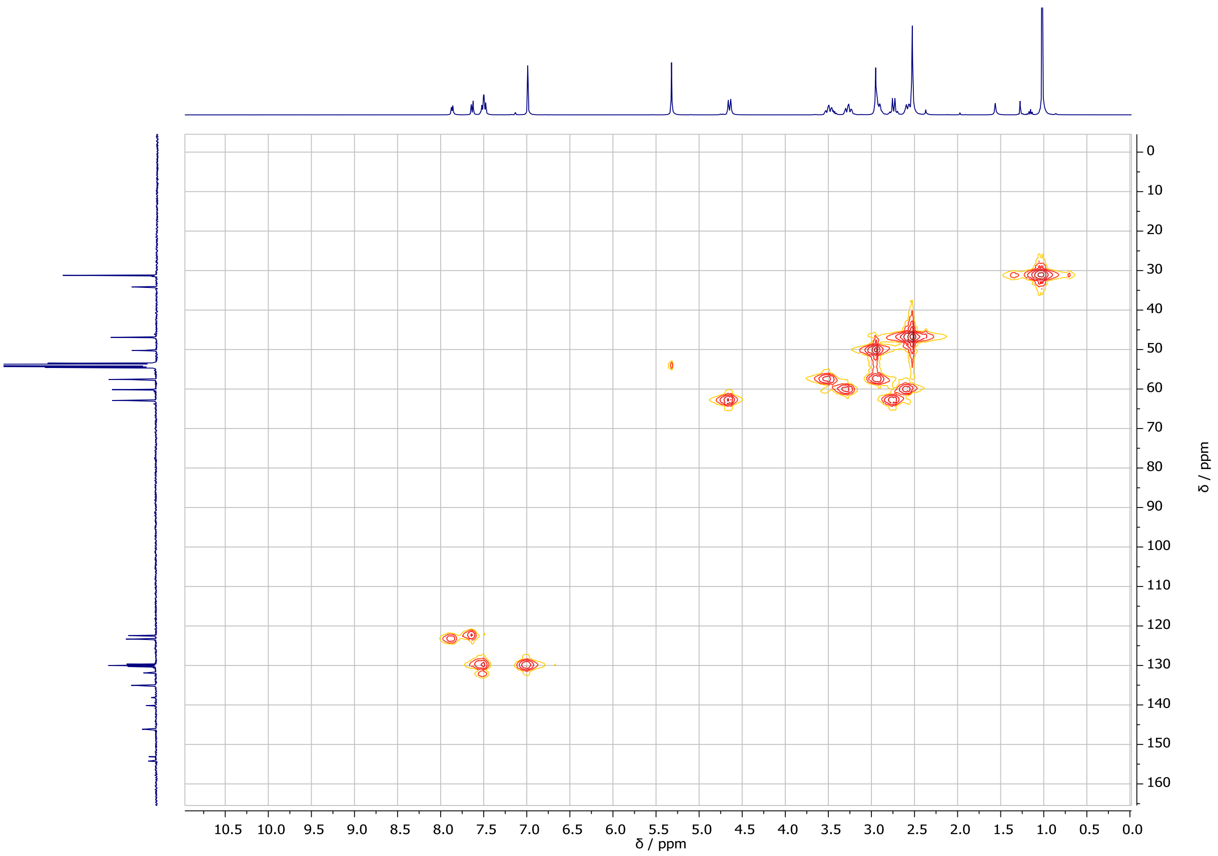

Figure S6: $^{1}H,^{13}C$ HSQC spectrum of **1** in CD_2Cl_2 at ambient temperature.

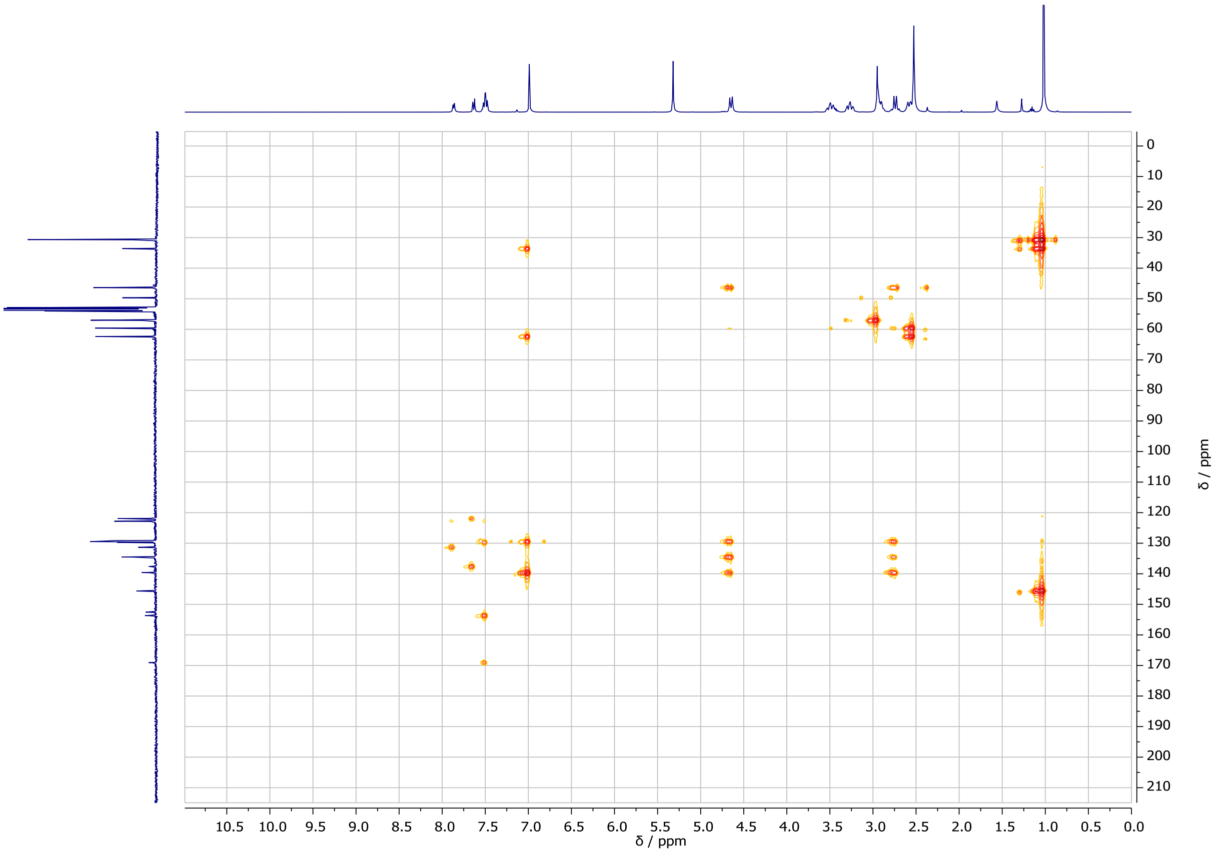

Figure S7. $^{1}H,^{13}C$ HMBC spectrum of **1** in CD_2Cl_2 at ambient temperature.

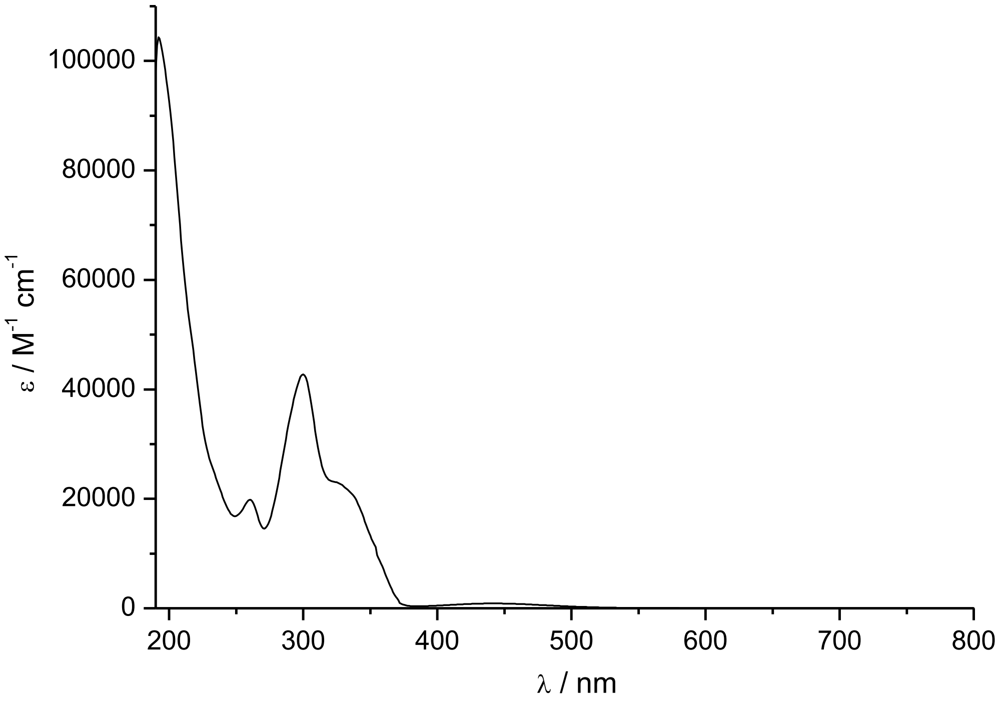

Figure S8. UV–vis spectrum of **1** (CH_3CN, [**1**] = 10^{-4} M).

2. Analytical data for compound 2.

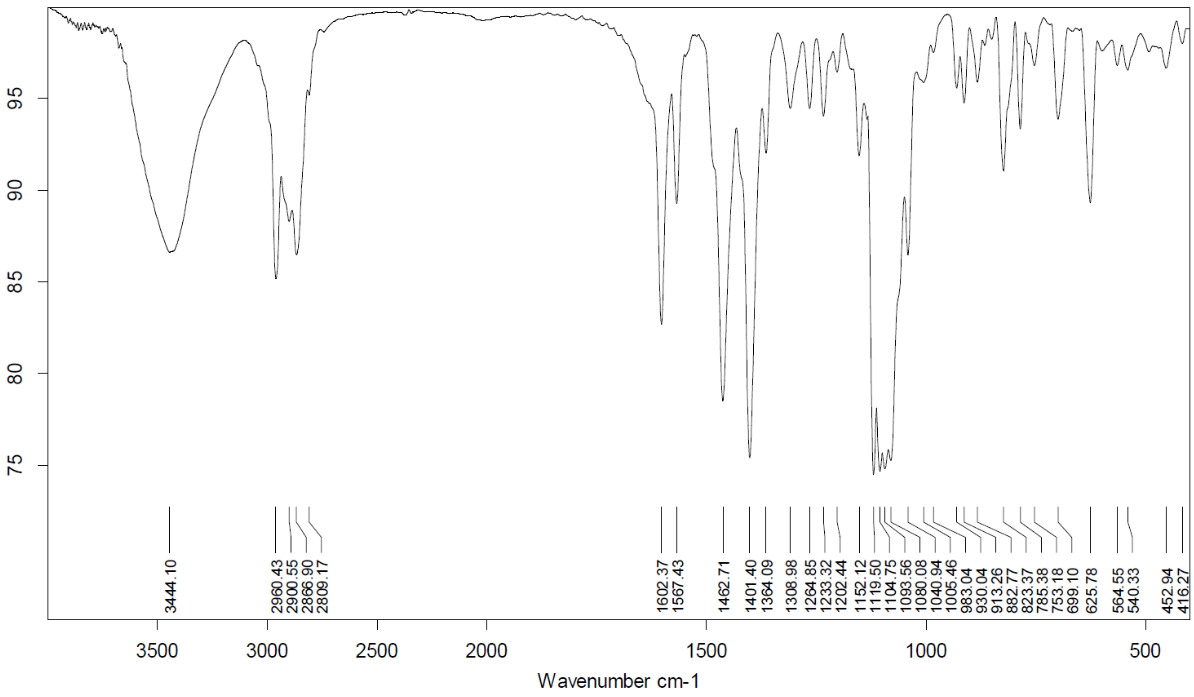

Figure S9. Infrared spectrum of **2**.

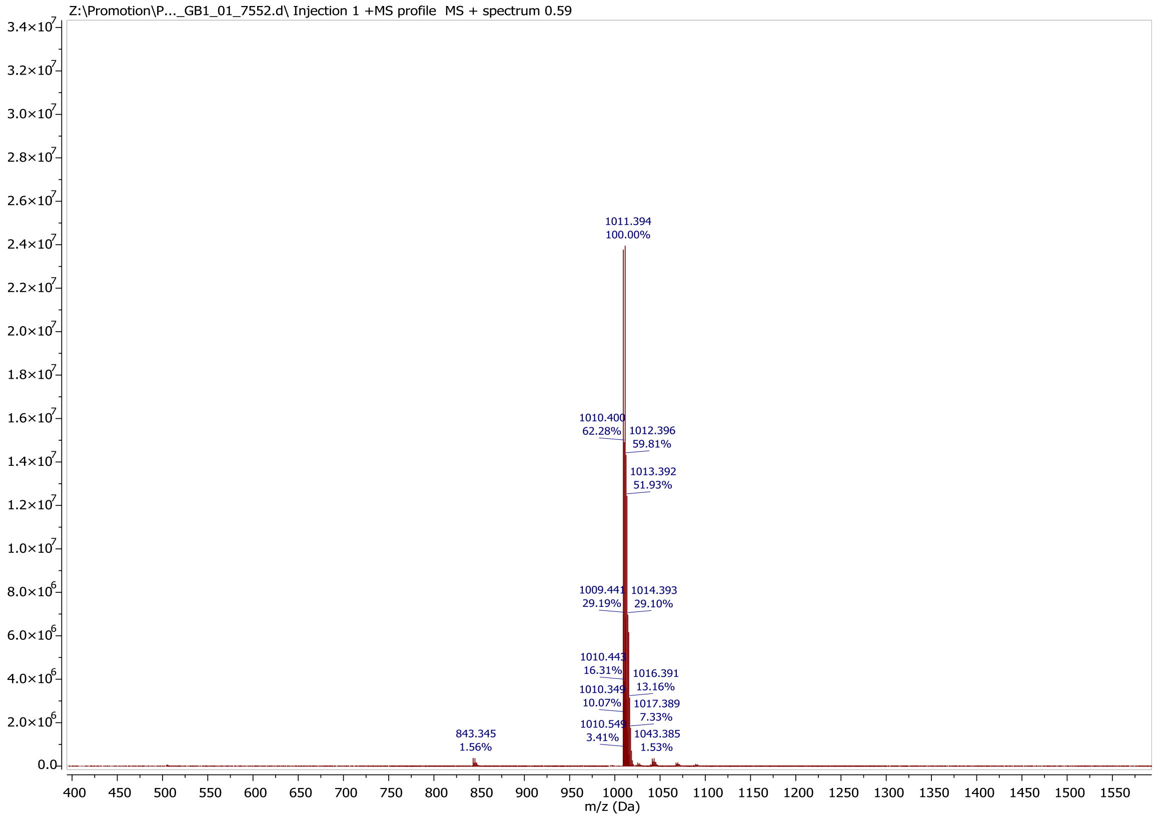

Figure S10. ESI mass spectrum of **2**.

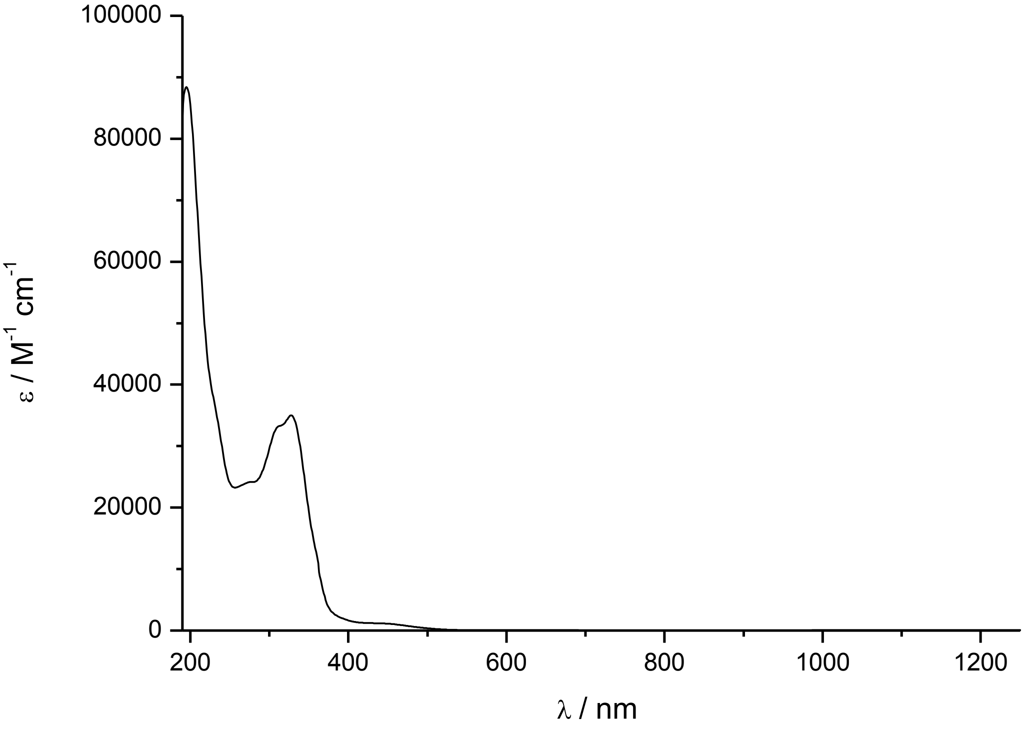

Figure S11. UV–vis spectrum of **2** (CH_3CN, [**2**] = 10^{-4} M).

Table S1. Experimental and calculated $\chi_M T$ [cm^3 K mol^{-1}] and μ_{eff} [μ_B] for **2**.

***T* [K]**	**$\chi_M T$ exp**	**$\chi_M T$ calc**	**μ_{eff} exp**	**μ_{eff} calc**
2.003	3.048	2.991	4.937	4.894
3.001	3.262	3.299	5.108	5.139
4.010	3.396	3.442	5.211	5.248
5.004	3.499	3.517	5.289	5.304
6.004	3.563	3.562	5.338	5.338
7.004	3.596	3.591	5.363	5.359
7.996	3.608	3.610	5.371	5.373
9.005	3.617	3.624	5.379	5.383
10.002	3.623	3.634	5.383	5.391
11.011	3.633	3.642	5.390	5.396
12.000	3.638	3.648	5.394	5.401
13.007	3.649	3.653	5.402	5.404
14.010	3.661	3.657	5.411	5.407
15.000	3.666	3.660	5.415	5.410
16.001	3.670	3.663	5.418	5.412
17.000	3.673	3.665	5.420	5.413
18.000	3.676	3.667	5.422	5.415

18.993	3.678	3.668	5.423	5.416
20.001	3.680	3.669	5.425	5.416
30.000	3.678	3.663	5.423	5.412
39.998	3.645	3.630	5.399	5.388
50.014	3.592	3.580	5.360	5.350
60.055	3.526	3.522	5.310	5.306
70.084	3.467	3.462	5.266	5.261
80.113	3.409	3.405	5.221	5.218
90.144	3.354	3.352	5.179	5.177
100.173	3.303	3.304	5.139	5.140
110.200	3.260	3.262	5.106	5.107
120.211	3.221	3.223	5.075	5.077
130.176	3.187	3.189	5.049	5.050
140.259	3.157	3.159	5.024	5.026
150.278	3.127	3.132	5.001	5.005
160.298	3.104	3.108	4.983	4.985
170.312	3.083	3.087	4.965	4.968
180.313	3.064	3.068	4.950	4.953
190.341	3.047	3.051	4.937	4.939
200.350	3.032	3.036	4.925	4.927
210.367	3.018	3.022	4.913	4.916
220.372	3.007	3.010	4.904	4.906
230.363	2.996	2.999	4.895	4.898
240.391	2.986	2.990	4.887	4.890
250.387	2.978	2.981	4.880	4.883
260.388	2.972	2.974	4.875	4.877
270.385	2.965	2.967	4.870	4.872
280.271	2.961	2.961	4.866	4.867
290.393	2.958	2.956	4.864	4.863
300.382	2.955	2.952	4.861	4.859

3. Analytical data for compound 3.

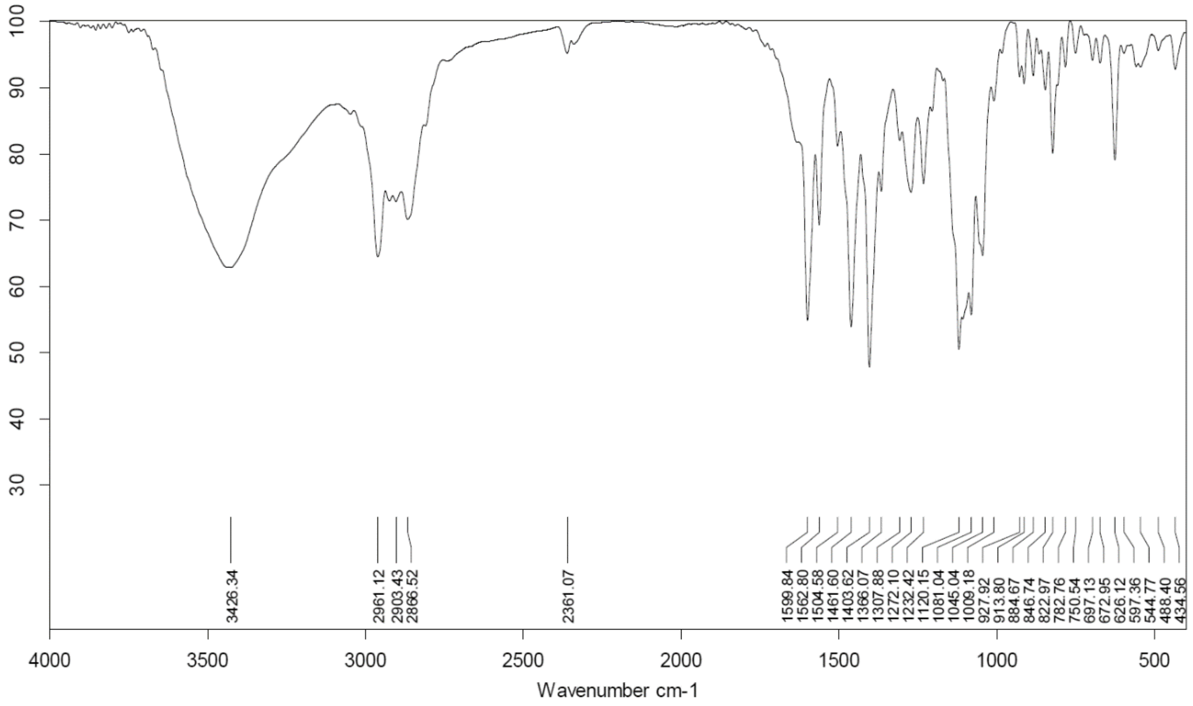

Figure S12. Infrared spectrum of **3**.

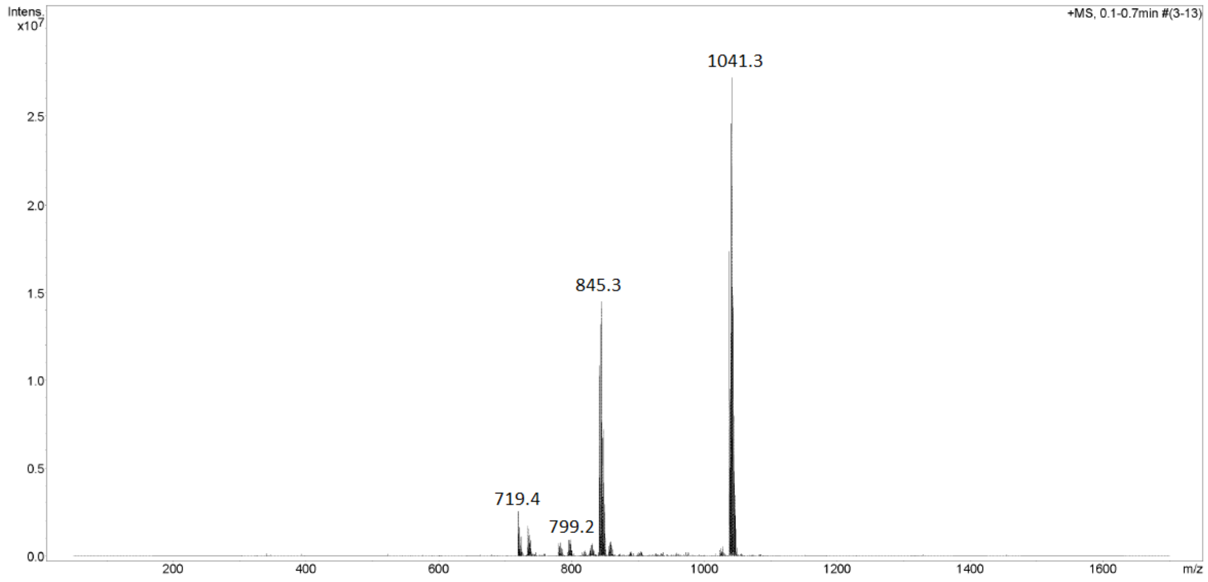

Figure S13. ESI mass spectrum of **3**.

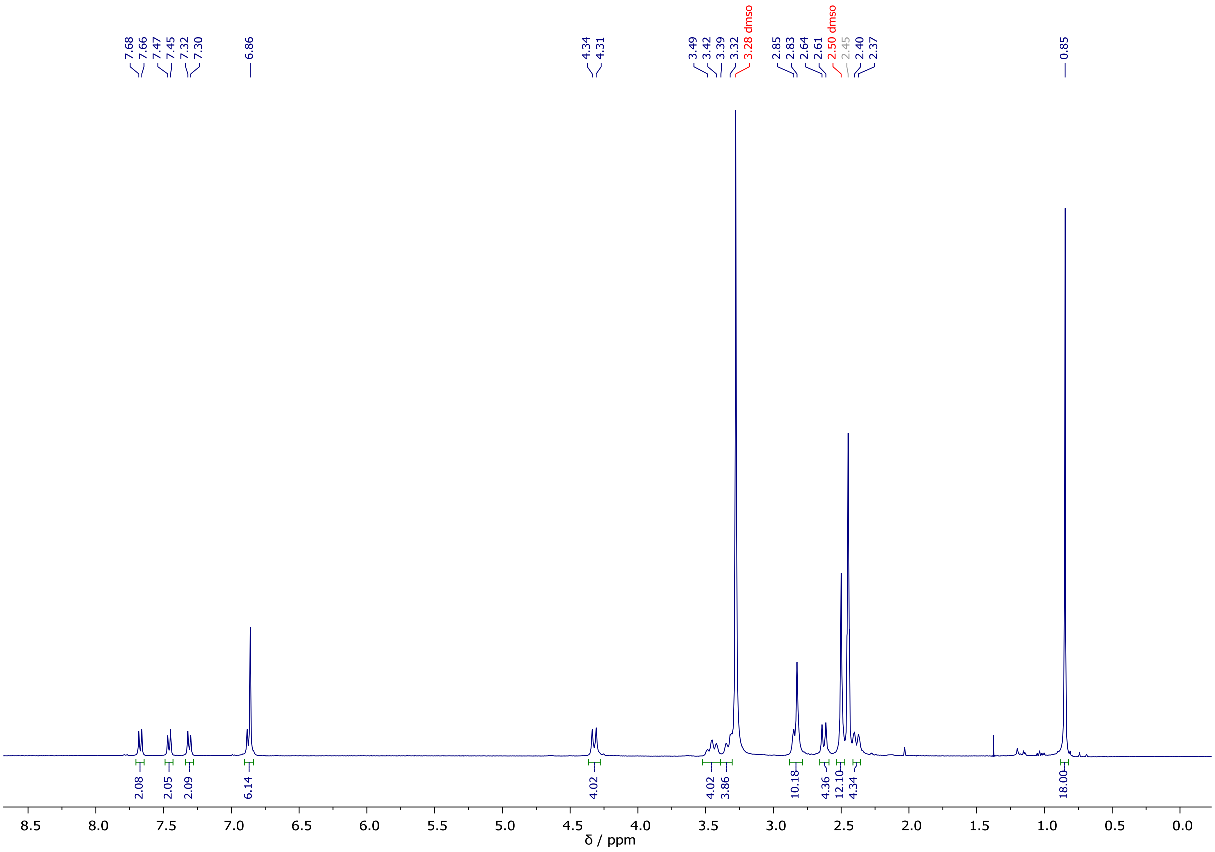

Figure S14. ^{1}H NMR spectrum of **3** in $(CD_3)_2SO$ at ambient temperature.

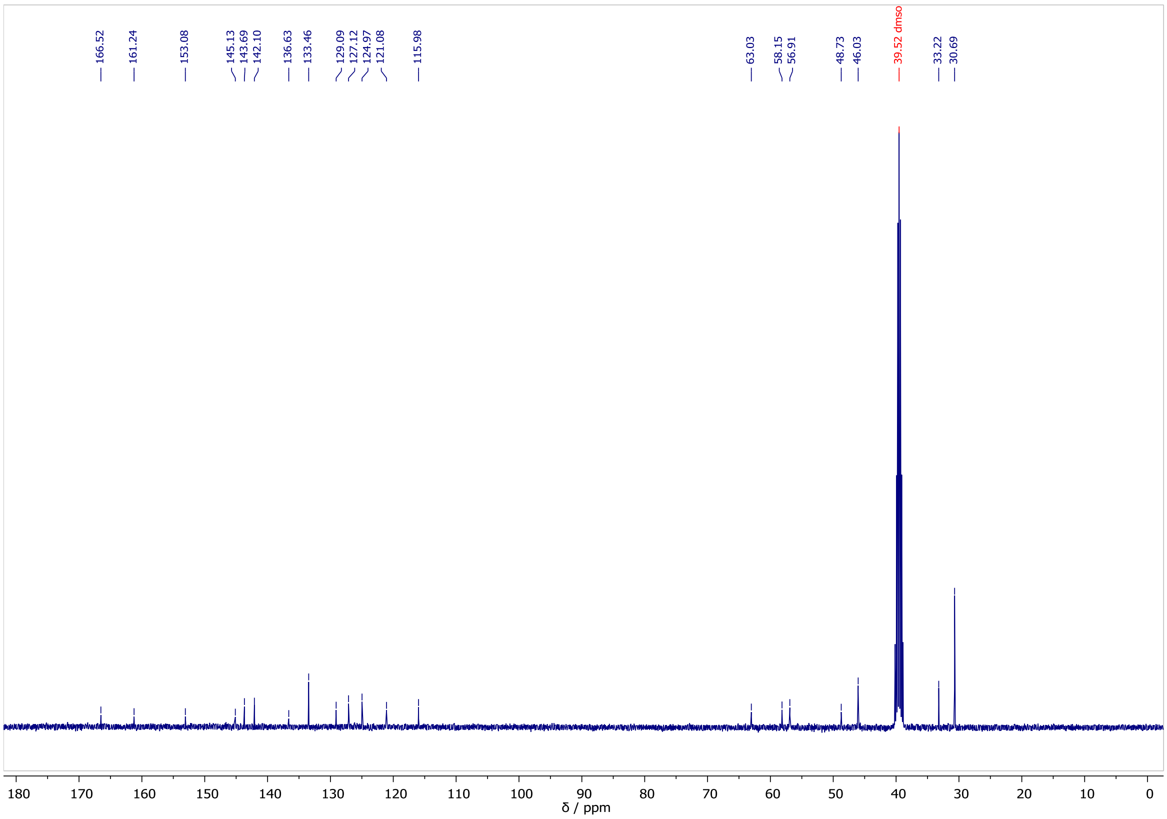

Figure S15. ^{13}C NMR spectrum of **3** in $(CD_3)_2SO$ at ambient temperature.

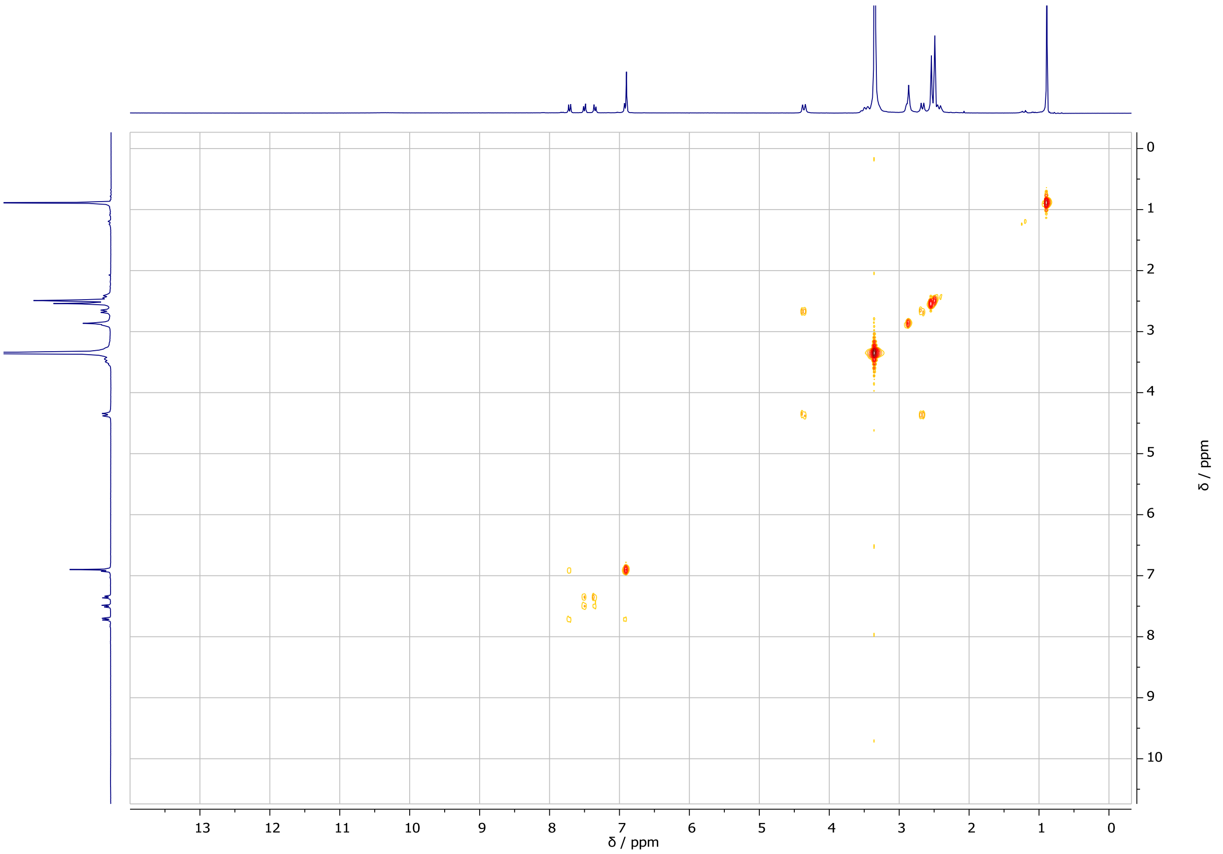

Figure S16. $^{1}H,^{1}H$ COSY spectrum of **3** in $(CD_3)_2SO$ at ambient temperature.

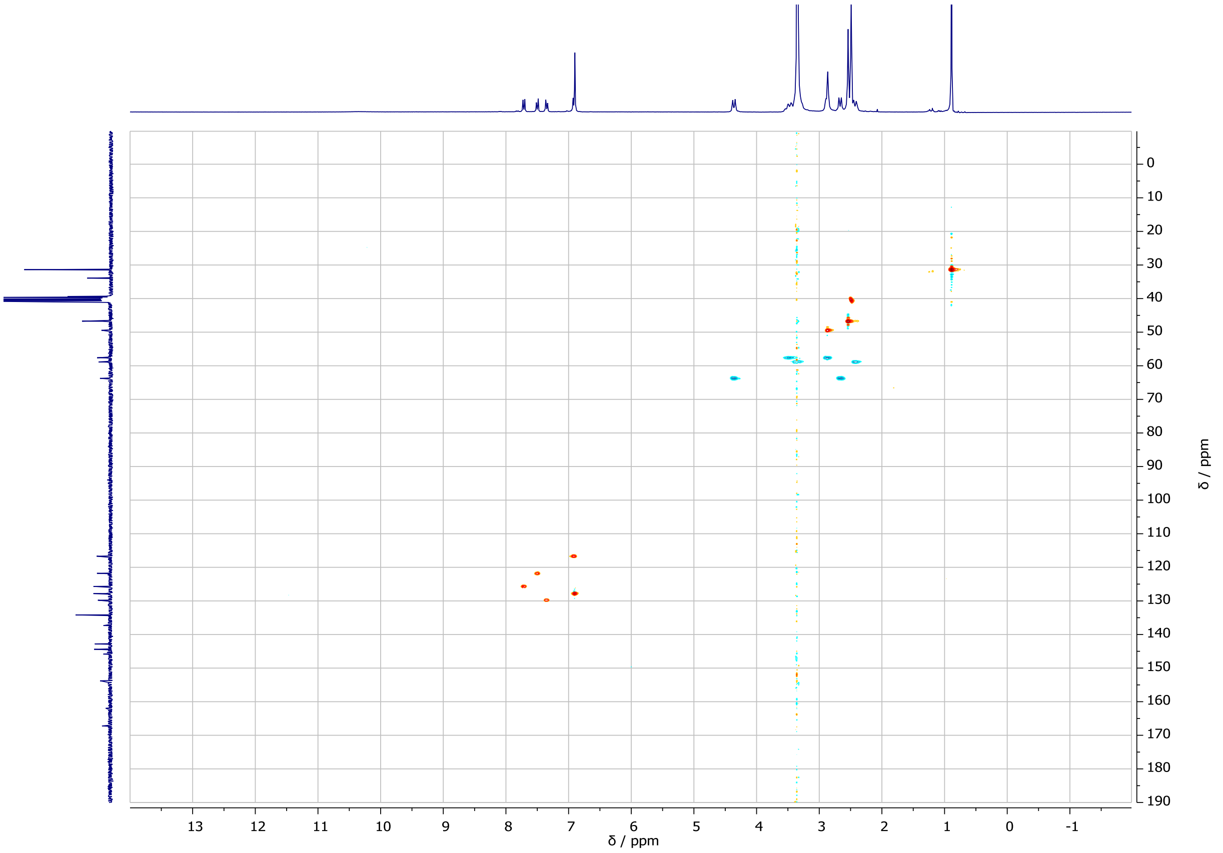

Figure S17. $^{1}H,^{13}C$ HSQC spectrum of **3** in $(CD_3)_2SO$ at ambient temperature.

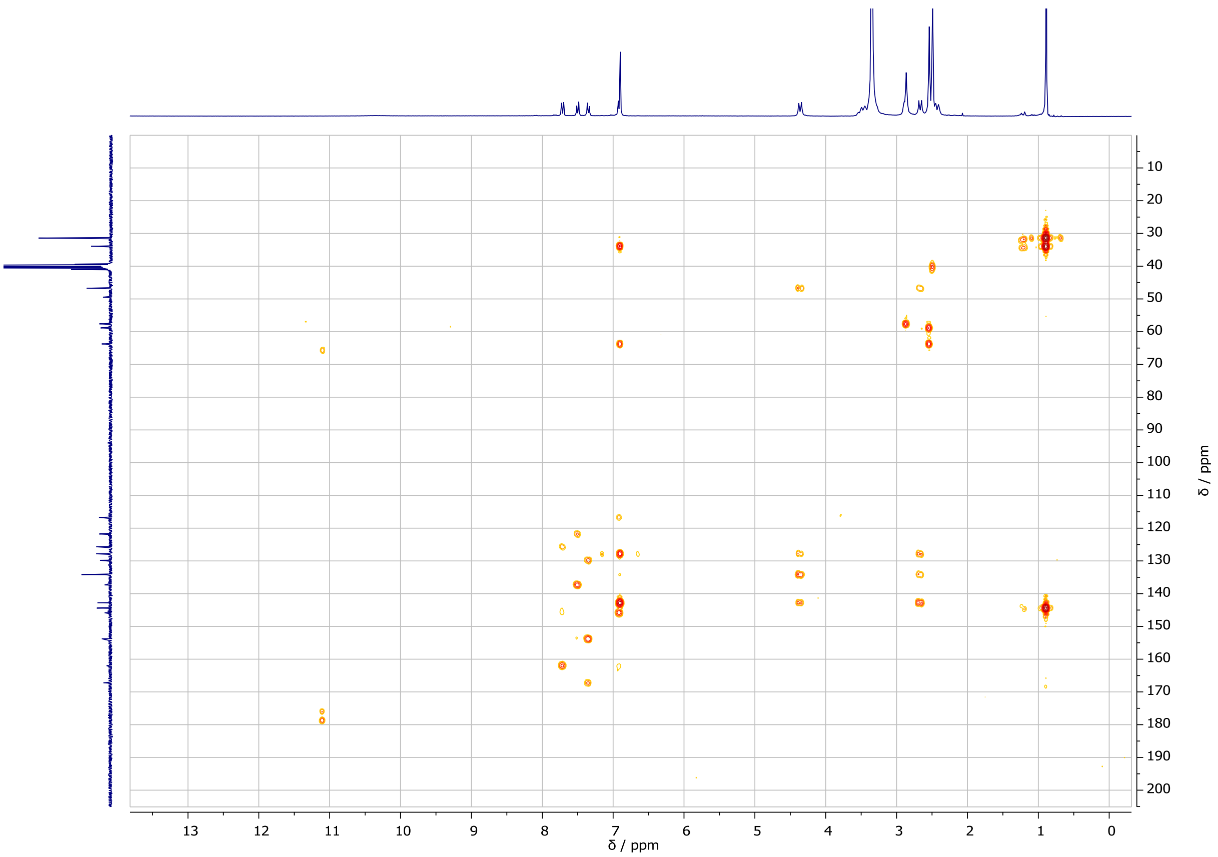

Figure S18. ^{1}H,^{13}C HMBC spectrum of **3** in $(CD_3)_2SO$ at ambient temperature.

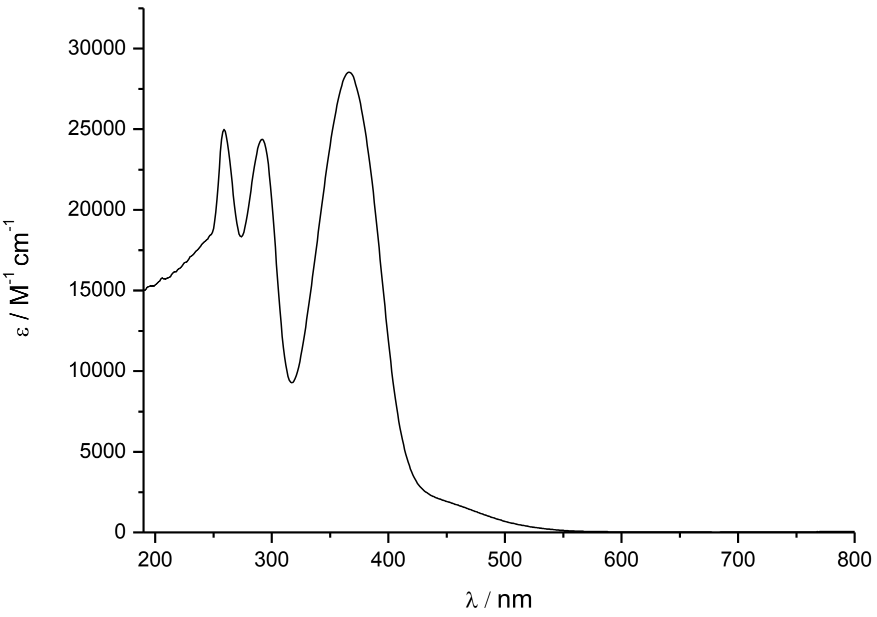

Figure S19. UV–vis spectrum of **3** (DMSO, [**3**] = 10^{-4} M).

4. Analytical data for compound 4.

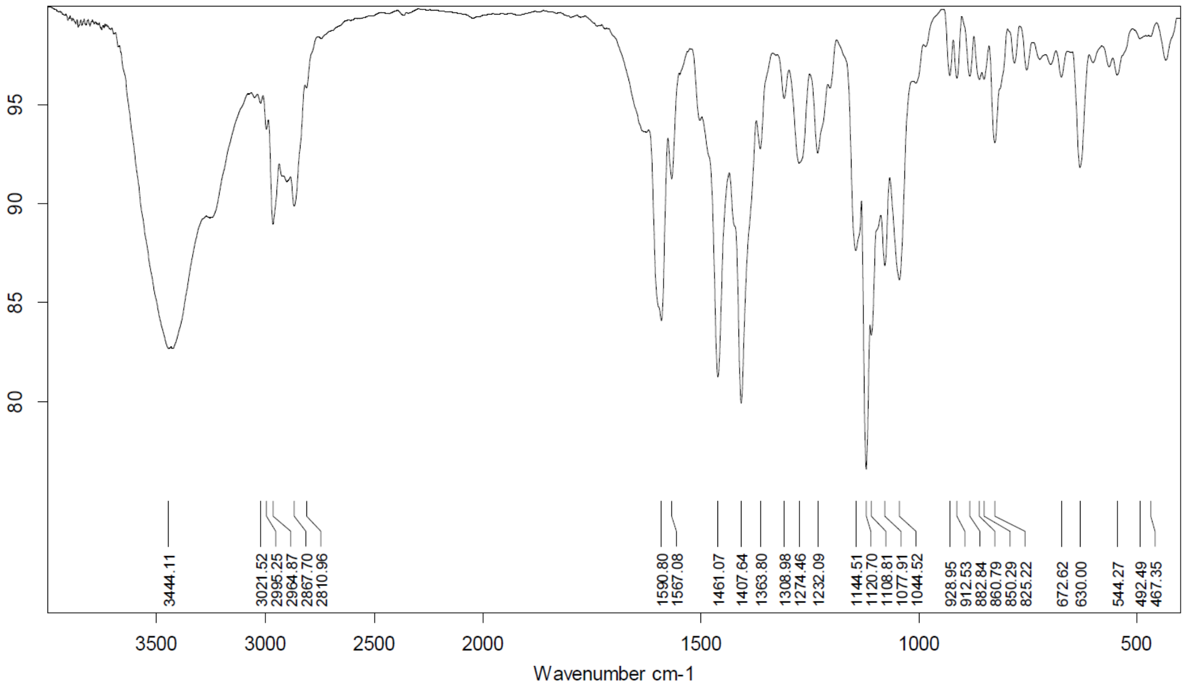

Figure 20. Infrared spectrum of **4**.

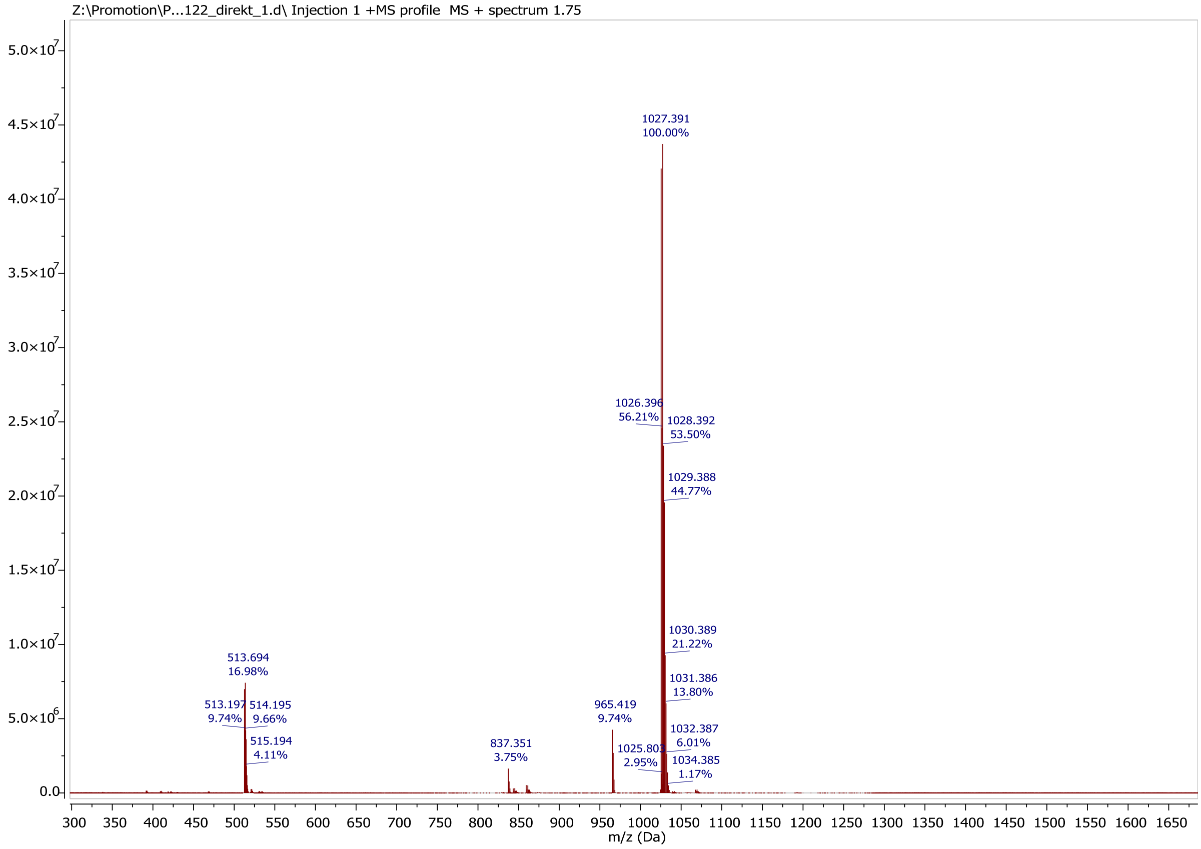

Figure S21. ESI mass spectrum of **4**.

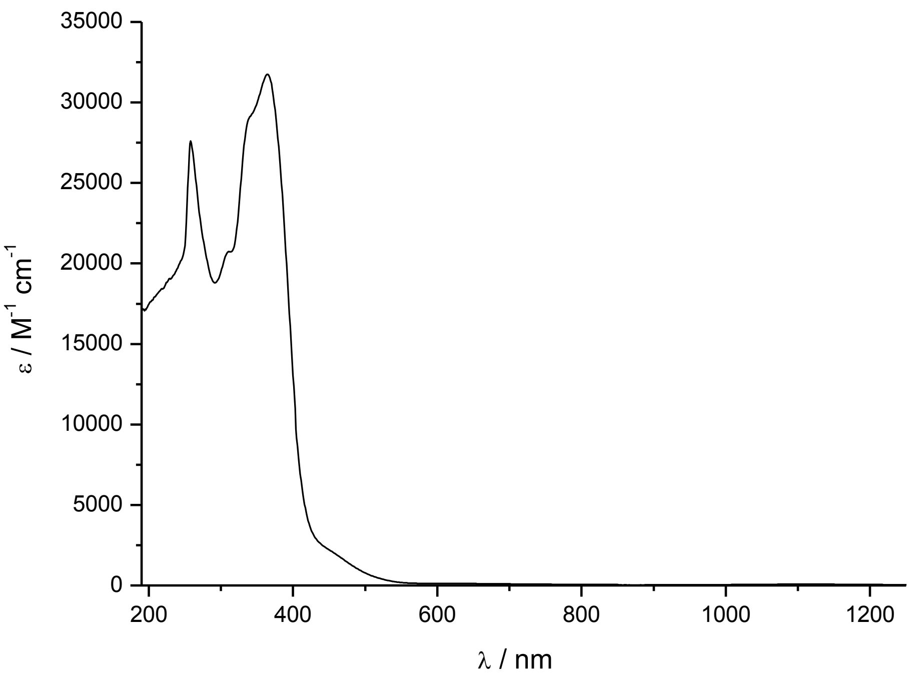

Figure S22. UV–vis spectrum of **4** (DMSO, [**4**] = 10^{-4} M).

Table S2. Experimental and calculated $\chi_M T$ [cm^3 K mol^{-1}] and μ_{eff} [μ_B] for **4**.

***T* [K]**	**$\chi_M T$ exp**	**$\chi_M T$ calc**	**μ_{eff} exp**	**μ_{eff} calc**
2.001	2.578	2.513	4.541	4.491
3.047	2.988	2.997	4.888	4.900
3.941	3.161	3.197	5.028	5.060
5.058	3.270	3.329	5.113	5.162
6.005	3.365	3.393	5.187	5.210
7.004	3.419	3.435	5.229	5.242
8.002	3.451	3.464	5.253	5.263
9.003	3.469	3.484	5.267	5.278
10.003	3.484	3.498	5.278	5.289
11.011	3.501	3.510	5.291	5.297
12.000	3.512	3.518	5.300	5.303
13.006	3.529	3.525	5.312	5.309
14.014	3.543	3.531	5.323	5.313
14.999	3.551	3.536	5.329	5.316
15.999	3.558	3.540	5.334	5.319
16.987	3.563	3.543	5.338	5.322
18.003	3.566	3.546	5.340	5.324

19.002	3.569	3.548	5.342	5.325
19.999	3.570	3.550	5.343	5.327
29.999	3.565	3.548	5.339	5.325
39.998	3.528	3.517	5.311	5.302
50.029	3.475	3.468	5.272	5.266
60.083	3.416	3.413	5.226	5.224
70.114	3.359	3.358	5.183	5.182
80.150	3.307	3.306	5.142	5.142
90.187	3.263	3.259	5.108	5.105
100.203	3.214	3.217	5.070	5.072
110.273	3.178	3.179	5.042	5.042
120.305	3.147	3.147	5.016	5.017
130.322	3.119	3.119	4.994	4.994
140.385	3.094	3.094	4.974	4.974
150.395	3.072	3.073	4.956	4.957
160.389	3.055	3.054	4.943	4.942
170.420	3.036	3.038	4.928	4.929
180.393	3.022	3.025	4.916	4.918
190.431	3.015	3.013	4.911	4.908
200.481	3.001	3.003	4.899	4.900
210.452	2.988	2.995	4.888	4.894
220.445	2.985	2.988	4.885	4.888
230.465	2.977	2.982	4.880	4.883
240.450	2.972	2.977	4.875	4.879
250.500	2.968	2.974	4.872	4.876
260.458	2.965	2.971	4.869	4.874
270.490	2.953	2.969	4.860	4.873
280.443	2.964	2.968	4.868	4.872
290.447	2.965	2.967	4.870	4.871
300.454	2.968	2.967	4.872	4.871

5. Analytical data for compound 5.

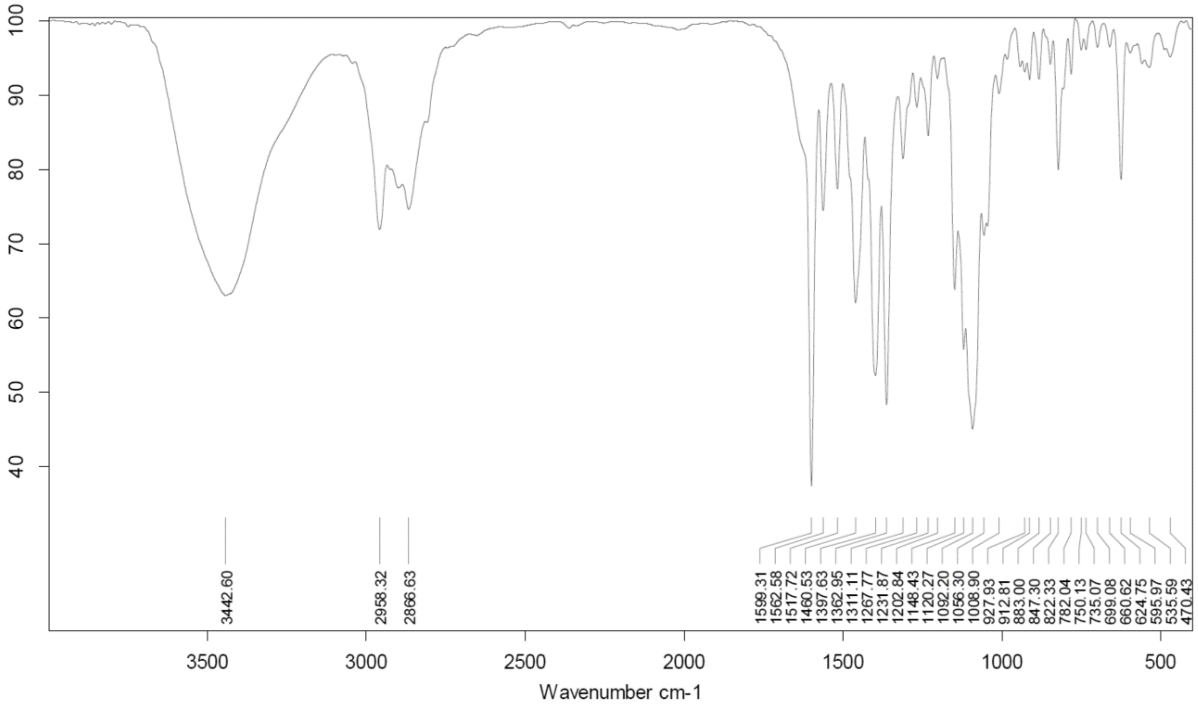

Figure S23. Infrared spectrum of **5**.

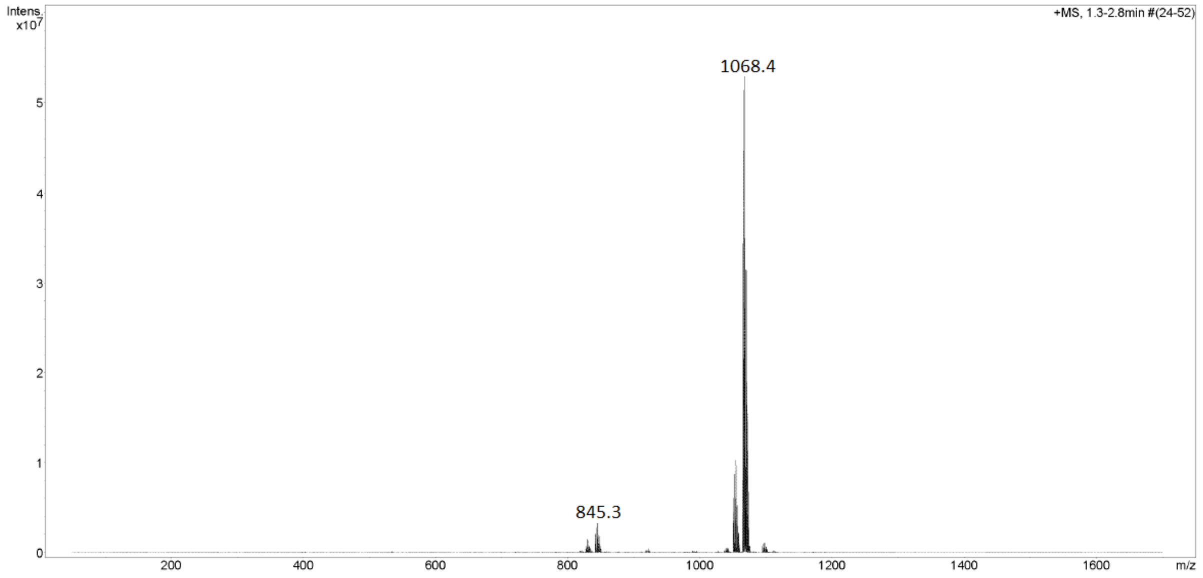

Figure S24. ESI mass spectrum of **5**.

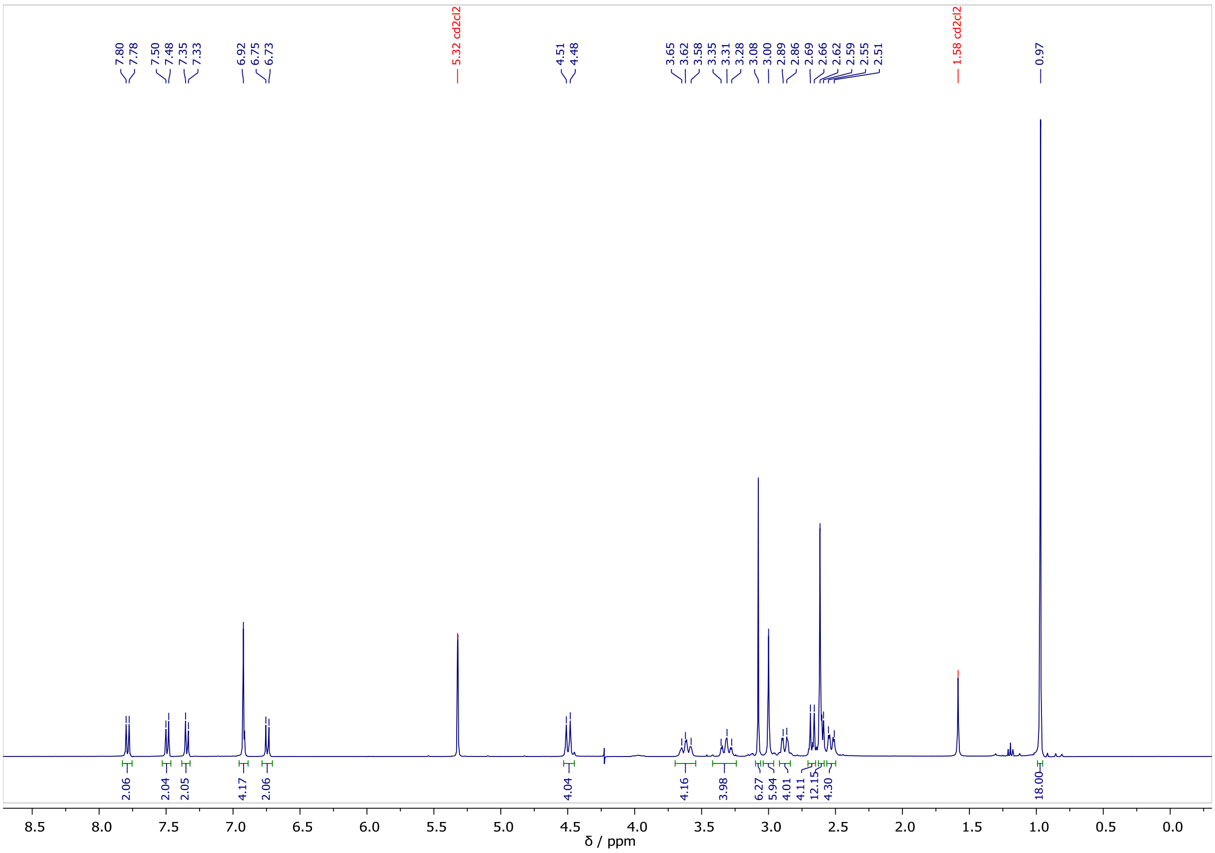

Figure S25. ^{1}H NMR spectrum of **5** in CD_2Cl_2 at ambient temperature.

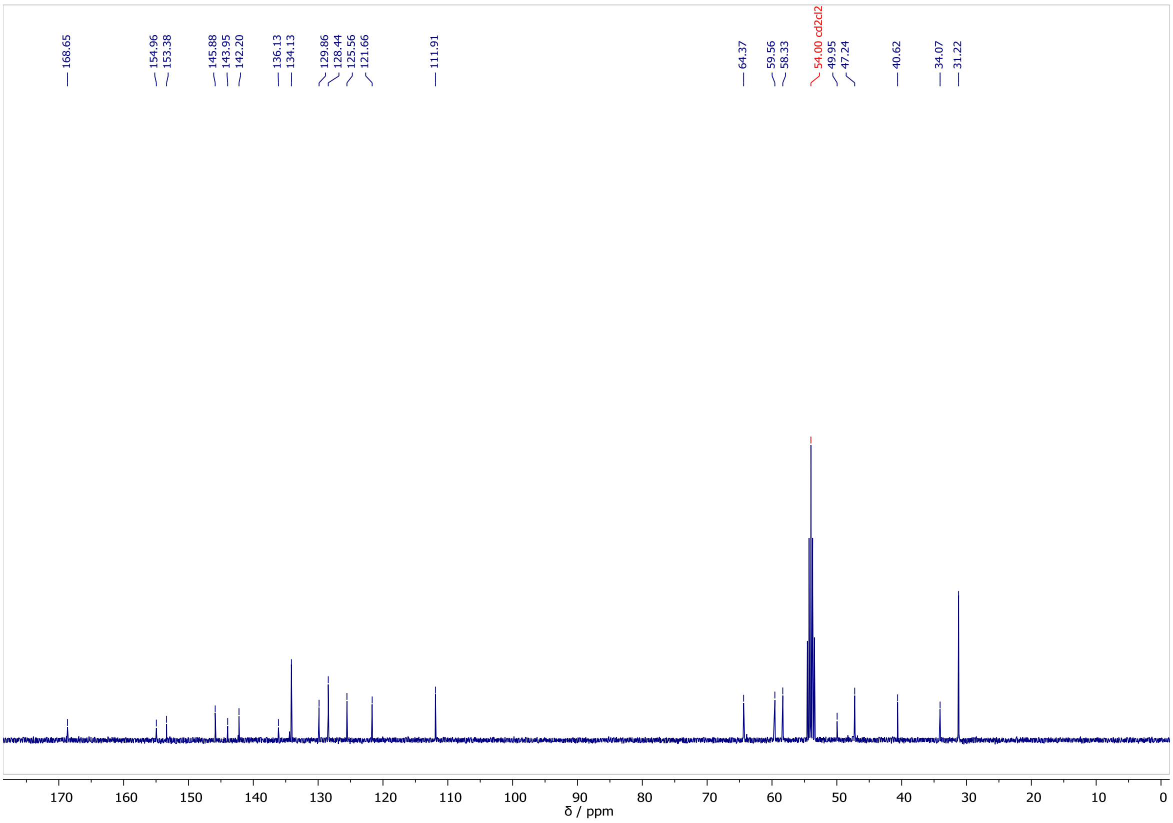

Figure S26. ^{13}C NMR spectrum of **5** in CD_2Cl_2 at ambient temperature.

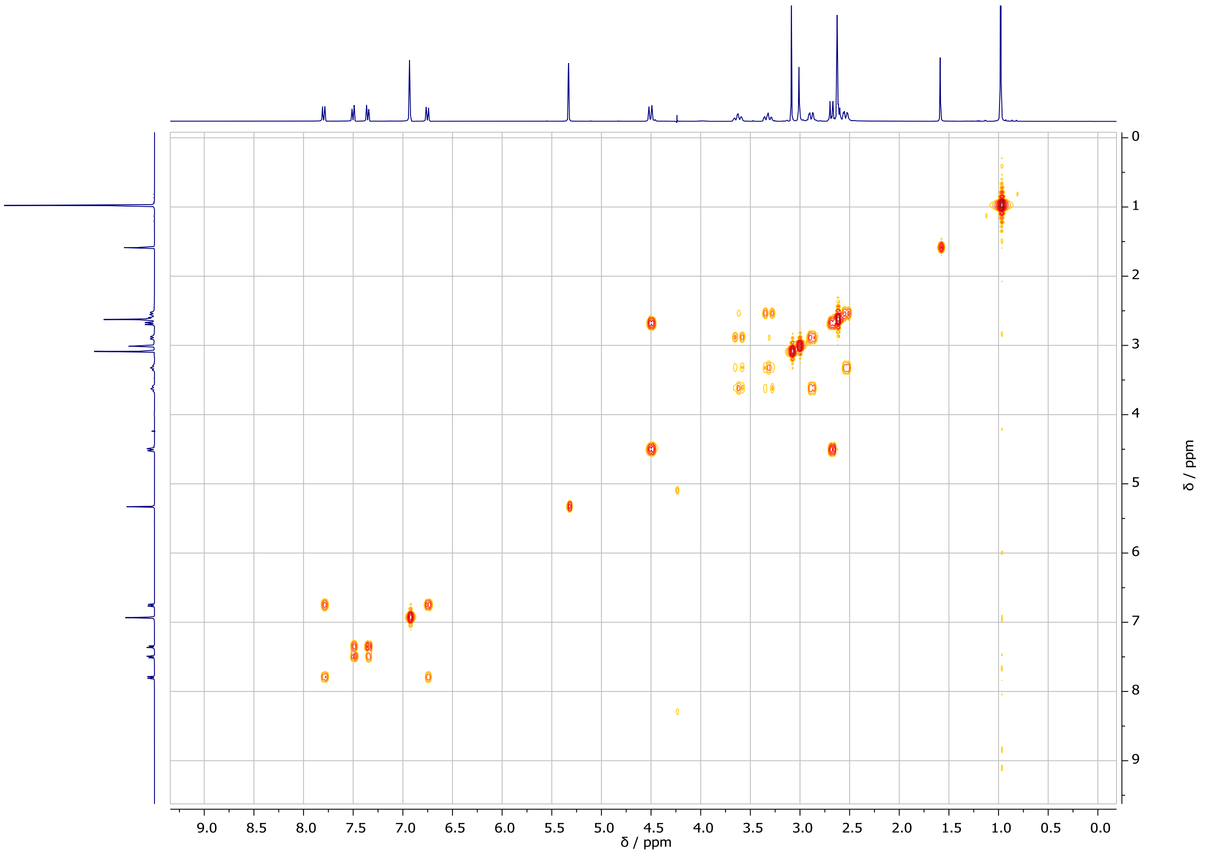

Figure S27. ^{1}H, ^{1}H COSY spectrum of **5** in CD_2Cl_2 at ambient temperature.

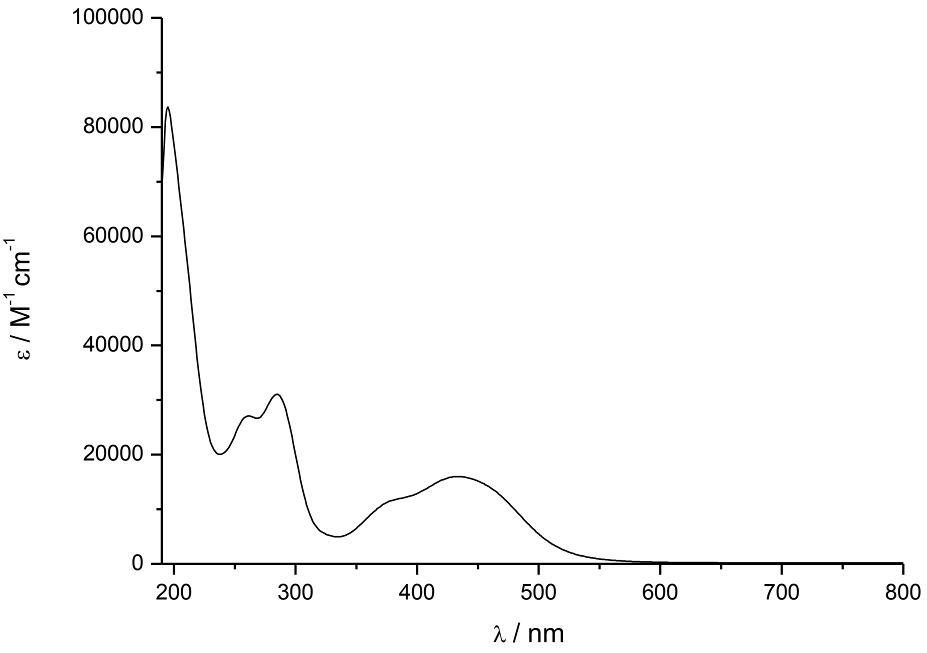

Figure S28. UV–vis spectrum of **5** (CH_3CN, [**5**] = 10^{-4} M).

6. Analytical data for compound 6.

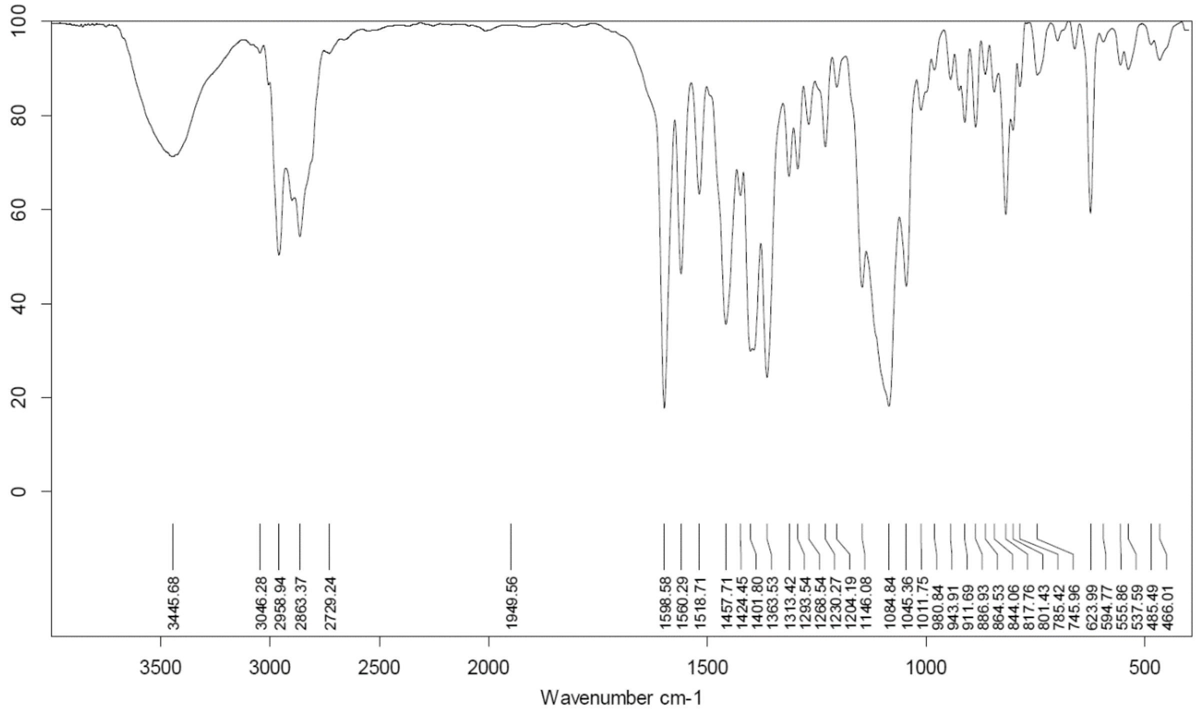

Figure S29. Infrared spectrum of **6**.

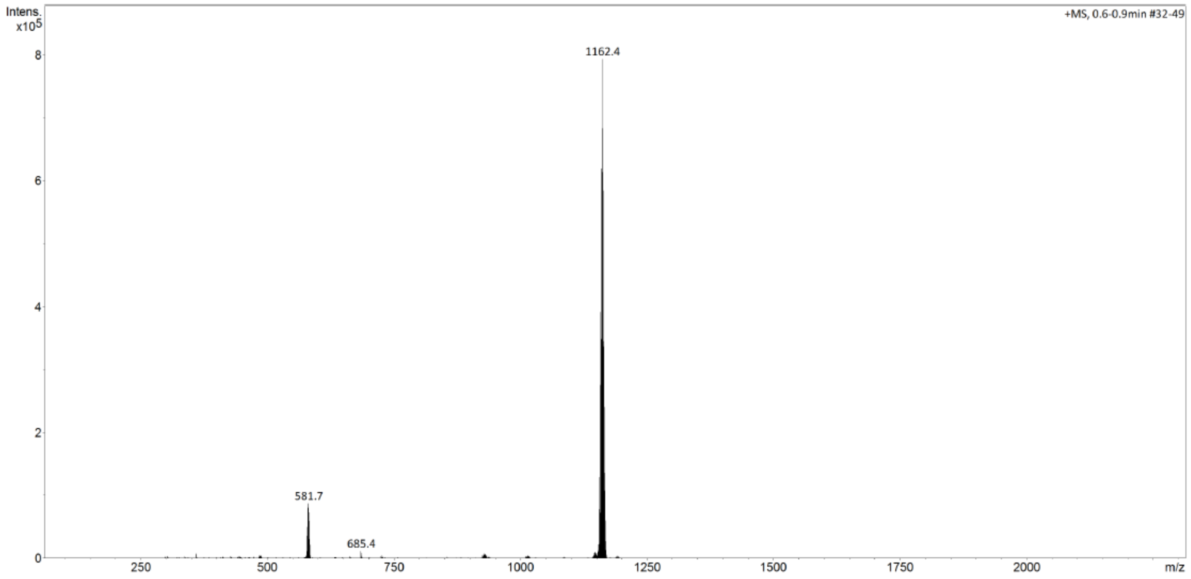

Figure S30. ESI mass spectrum of **6**.

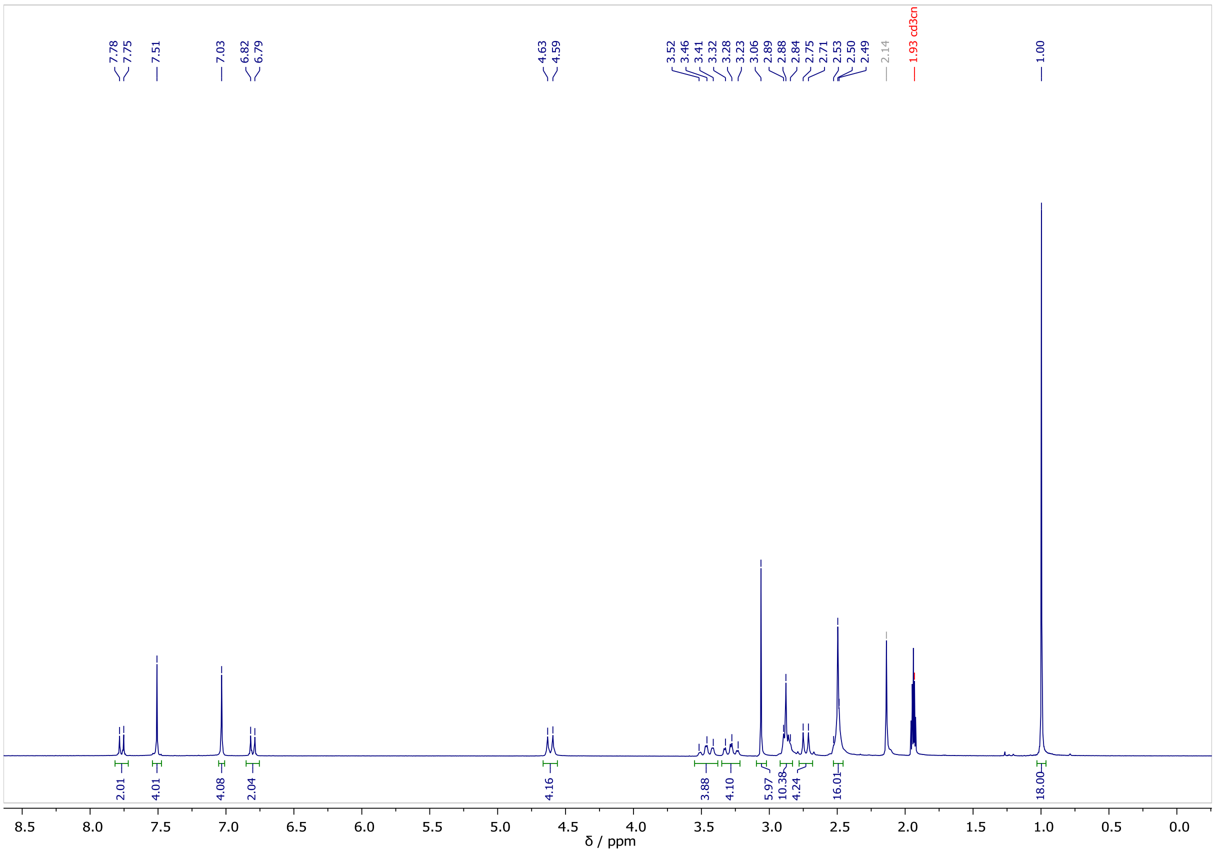

Figure S31. ^{1}H NMR spectrum of **6** in CD_3CN at ambient temperature.

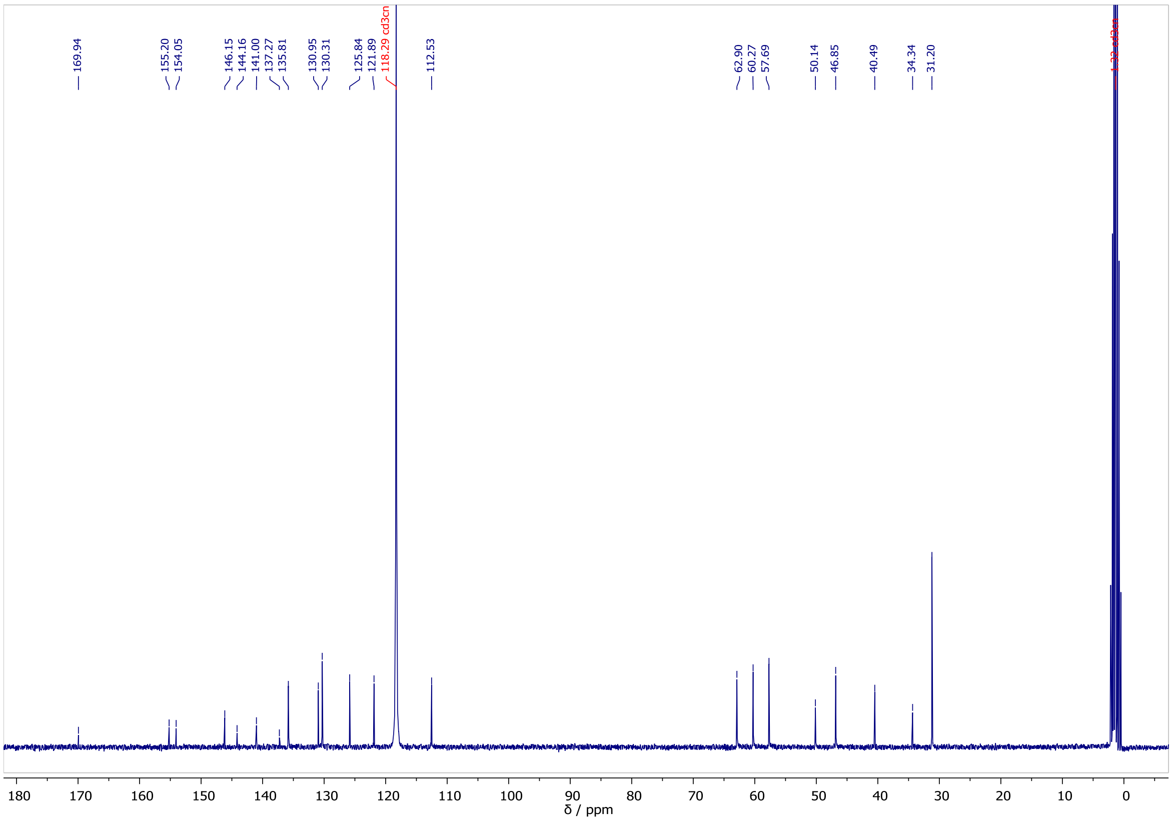

Figure S32. ^{13}C NMR spectrum of **6** in CD_3CN at ambient temperature.

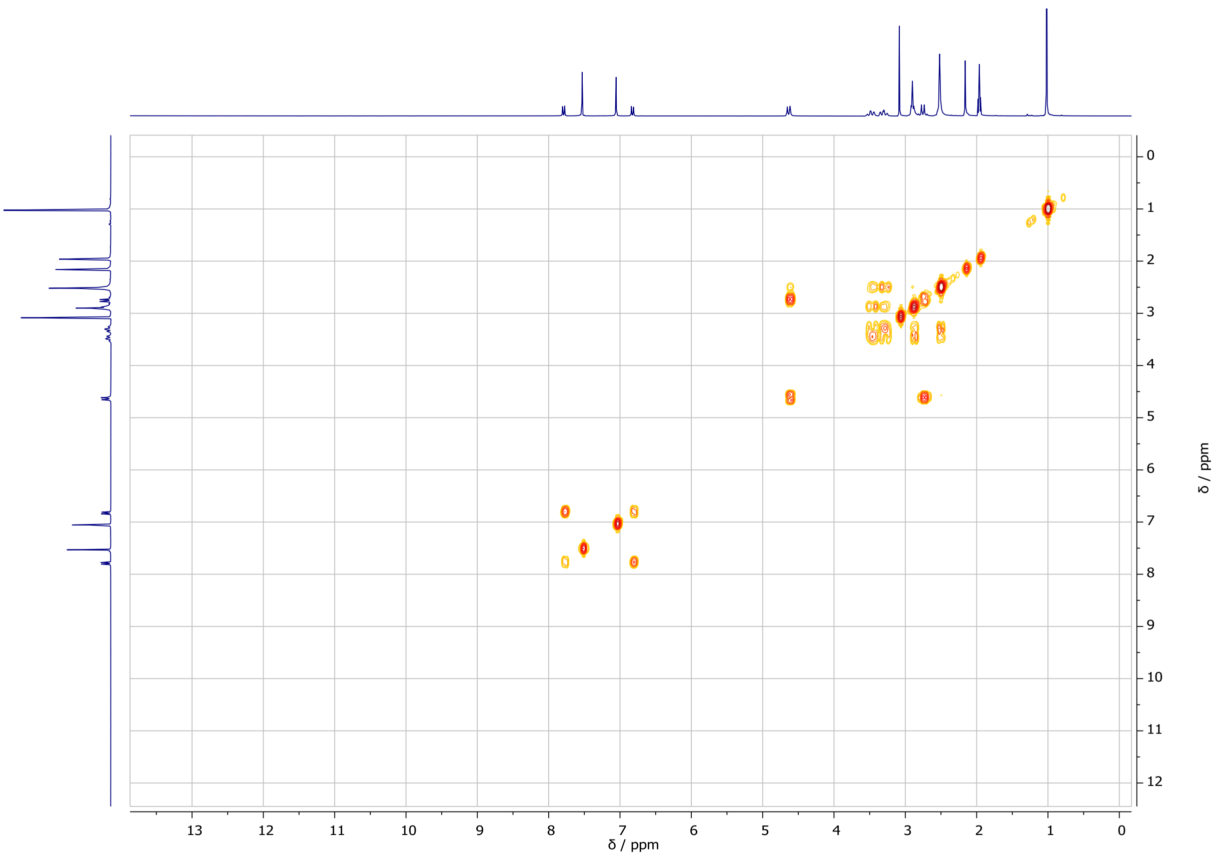

Figure S33. $^{1}H,^{1}H$ COSY spectrum of **6** in CD_3CN at ambient temperature.

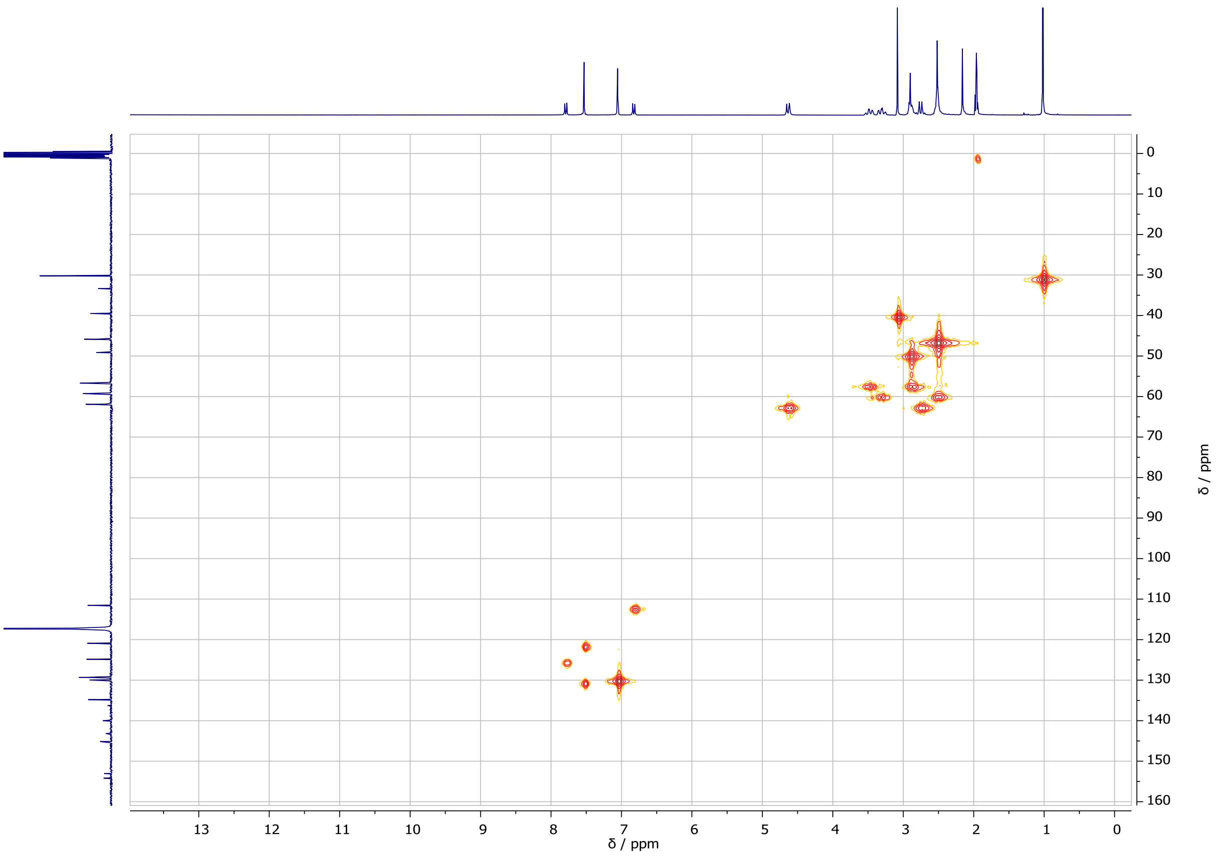

Figure 34: $^{1}H,^{13}C$ HSQC spectrum of **6** in CD_3CN at ambient temperature.

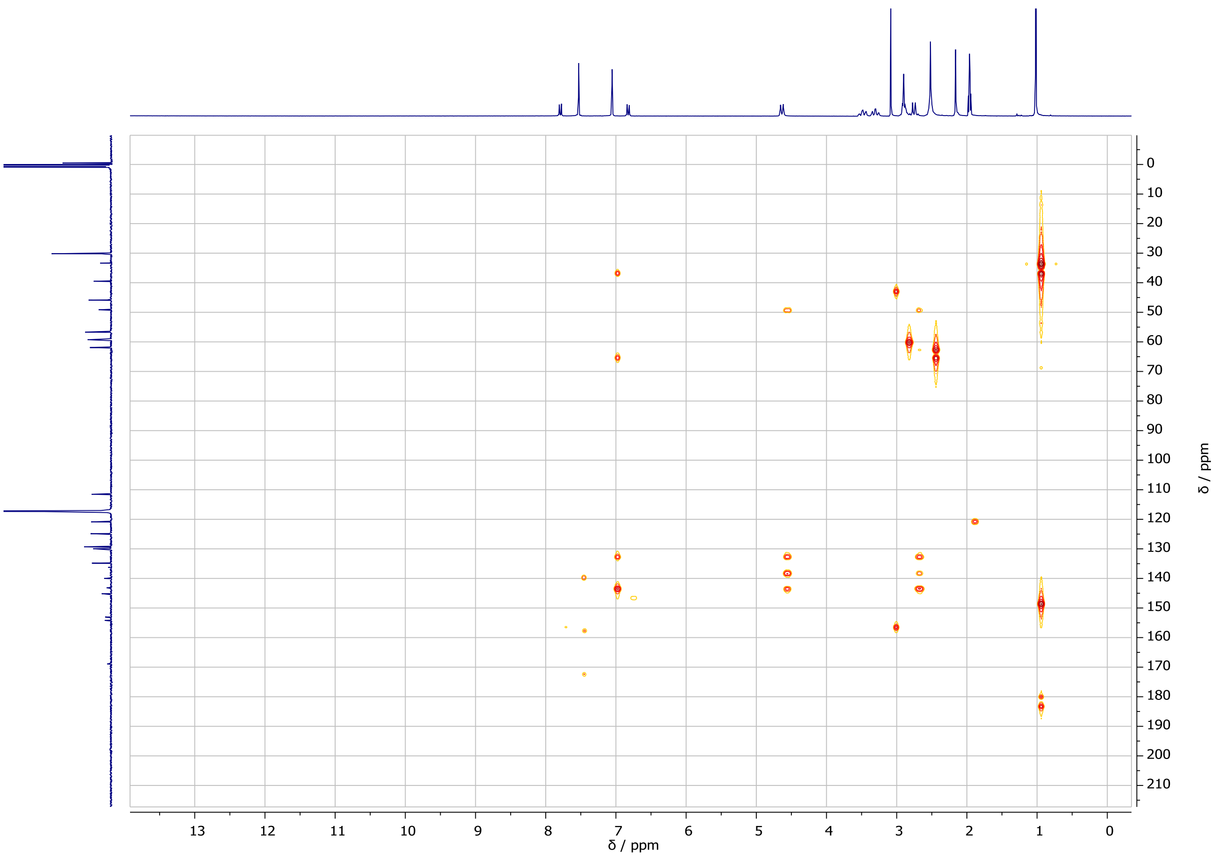

Figure S35. $^{1}H,^{13}C$ HMBC spectrum of **6** in CD_3CN at ambient temperature.

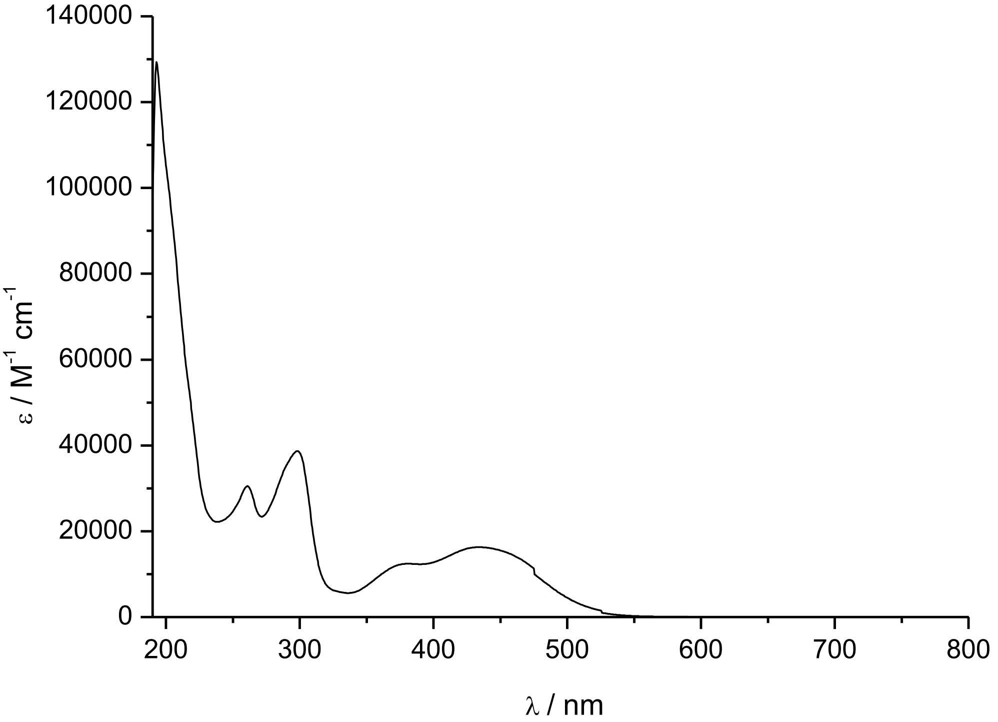

Figure S36. UV–vis spectrum of **6**, (CH_3CN, [**6**] = 10^{-4} M).

7. Analytical data for compound 7.

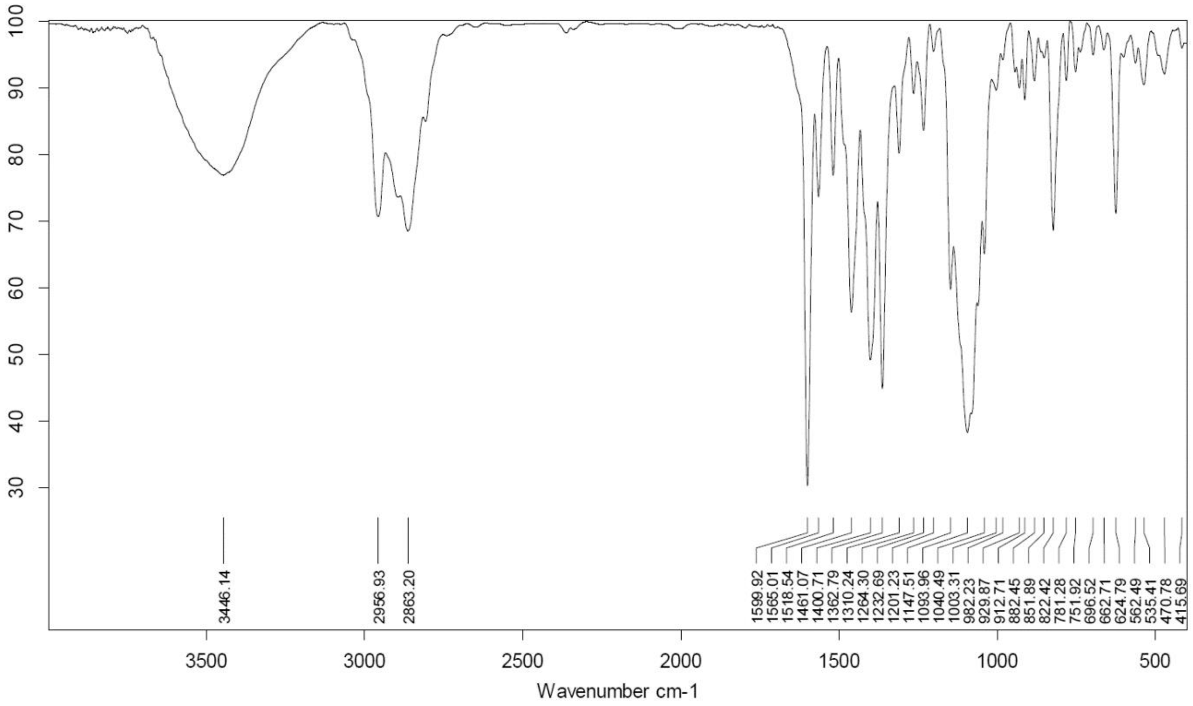

Figure S37. Infrared spectrum of **7**.

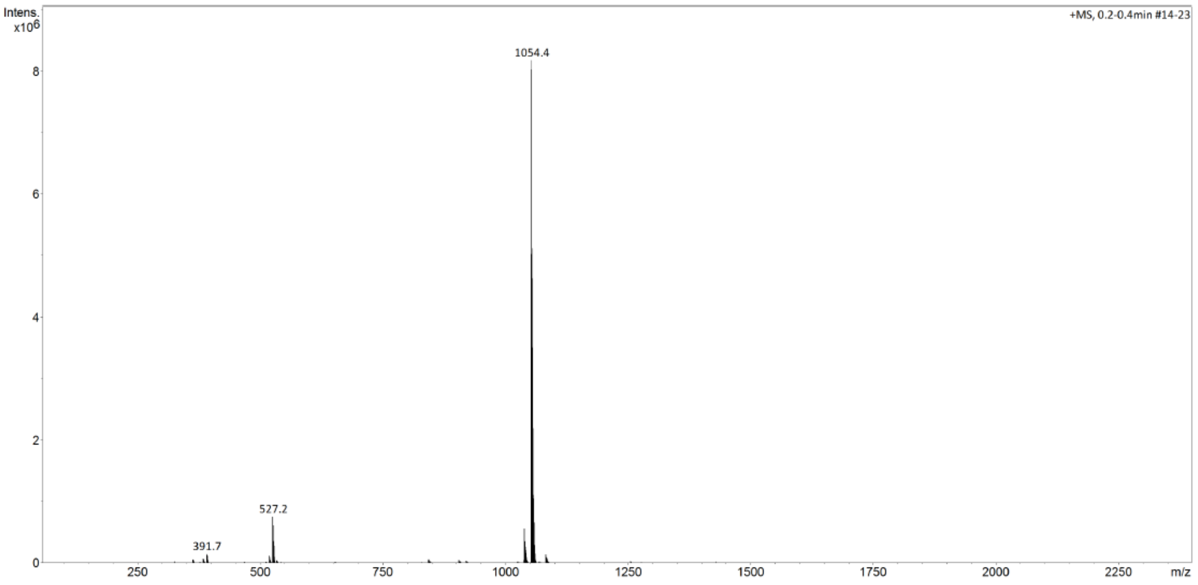

Figure S38. ESI mass spectrum of **7**.

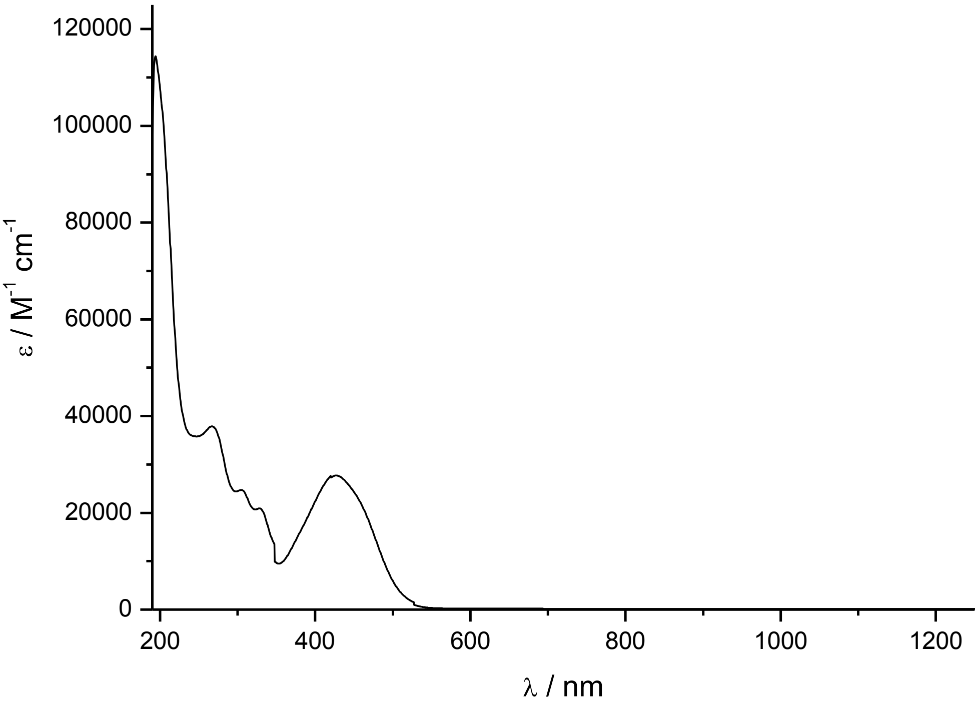

Figure S39. UV–vis spectrum of **7**, (CH_3CN, [**7**] = 10^{-4} M).

Table S3. Experimental and calculated $\chi_M T$ [cm^3 K mol^{-1}] and μ_{eff} [μ_B] for **7**.

T [K]	$\chi_M T_{exp}$	$\chi_M T_{calc}$	$\mu_{eff\ exp}$	$\mu_{eff\ calc}$
1.984	3.006	2.921	4.903	4.839
2.999	3.217	3.261	5.072	5.110
4.005	3.391	3.419	5.208	5.231
5.013	3.451	3.505	5.254	5.296
6.009	3.522	3.556	5.307	5.334
7.008	3.560	3.590	5.336	5.358
8.007	3.588	3.612	5.357	5.375
9.006	3.608	3.629	5.372	5.387
10.005	3.624	3.641	5.383	5.395
11.030	3.643	3.650	5.398	5.402
12.014	3.660	3.657	5.410	5.408
13.039	3.674	3.663	5.421	5.412
14.068	3.687	3.668	5.430	5.415
15.004	3.692	3.672	5.434	5.418
16.003	3.695	3.675	5.436	5.420
17.004	3.699	3.678	5.439	5.422

18.004	3.700	3.680	5.440	5.424
19.029	3.702	3.682	5.441	5.425
19.999	3.703	3.683	5.442	5.426
29.998	3.694	3.676	5.435	5.421
40.020	3.652	3.640	5.404	5.394
50.029	3.595	3.586	5.362	5.355
59.999	3.535	3.526	5.317	5.309
69.943	3.475	3.465	5.271	5.264
79.828	3.417	3.409	5.228	5.221
89.807	3.362	3.356	5.185	5.180
99.951	3.307	3.309	5.142	5.143
110.643	3.257	3.264	5.104	5.109
119.707	3.229	3.230	5.082	5.082
133.920	3.179	3.185	5.042	5.046
139.656	3.166	3.169	5.032	5.034
155.028	3.126	3.130	5.000	5.003
159.697	3.114	3.120	4.991	4.995
174.538	3.089	3.091	4.970	4.972
179.857	3.076	3.082	4.960	4.965
195.008	3.055	3.059	4.943	4.946
201.993	3.042	3.050	4.932	4.939
215.961	3.027	3.034	4.920	4.926
224.884	3.020	3.025	4.914	4.919
235.743	3.009	3.016	4.906	4.911
245.410	3.003	3.009	4.901	4.905
255.540	2.997	3.002	4.896	4.900
265.344	2.987	2.996	4.887	4.895
274.253	2.987	2.992	4.887	4.892
284.555	2.988	2.988	4.888	4.888
294.164	2.986	2.984	4.886	4.885
303.411	2.988	2.982	4.888	4.883

8. Analytical data for compound 8.

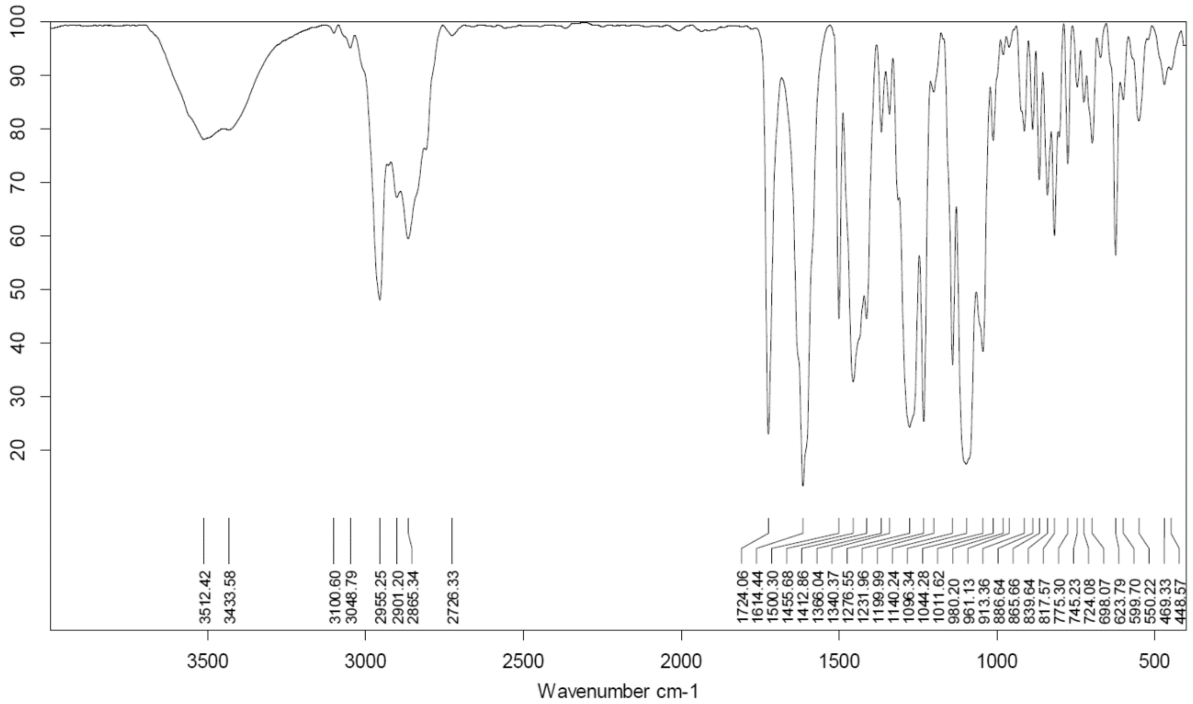

Figure S40. Infrared spectrum of **8**.

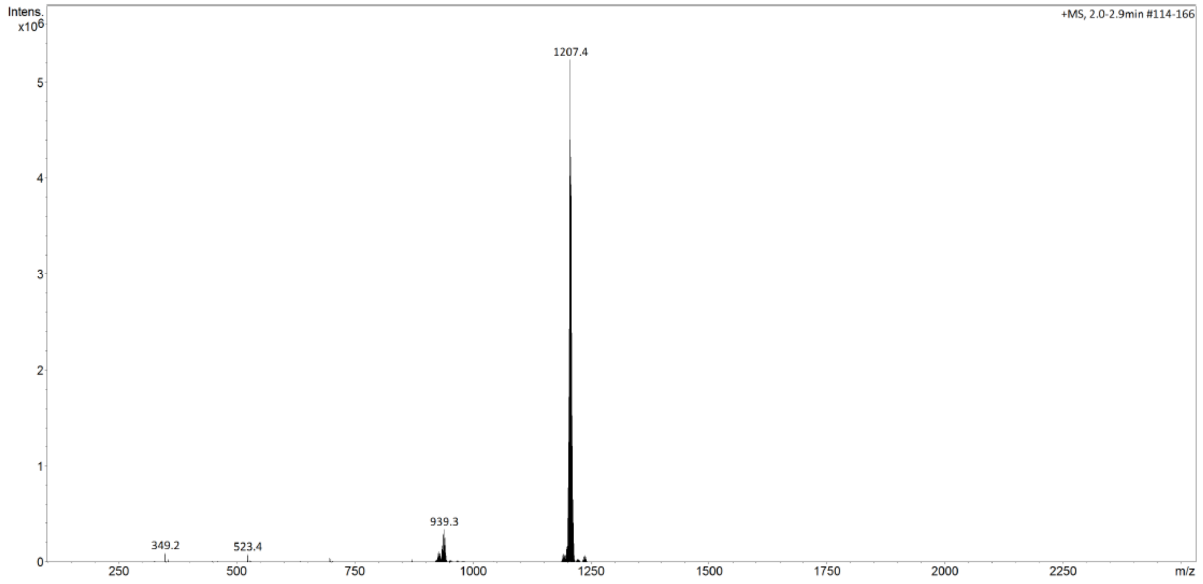

Figure S41. ESI mass spectrum of **8**.

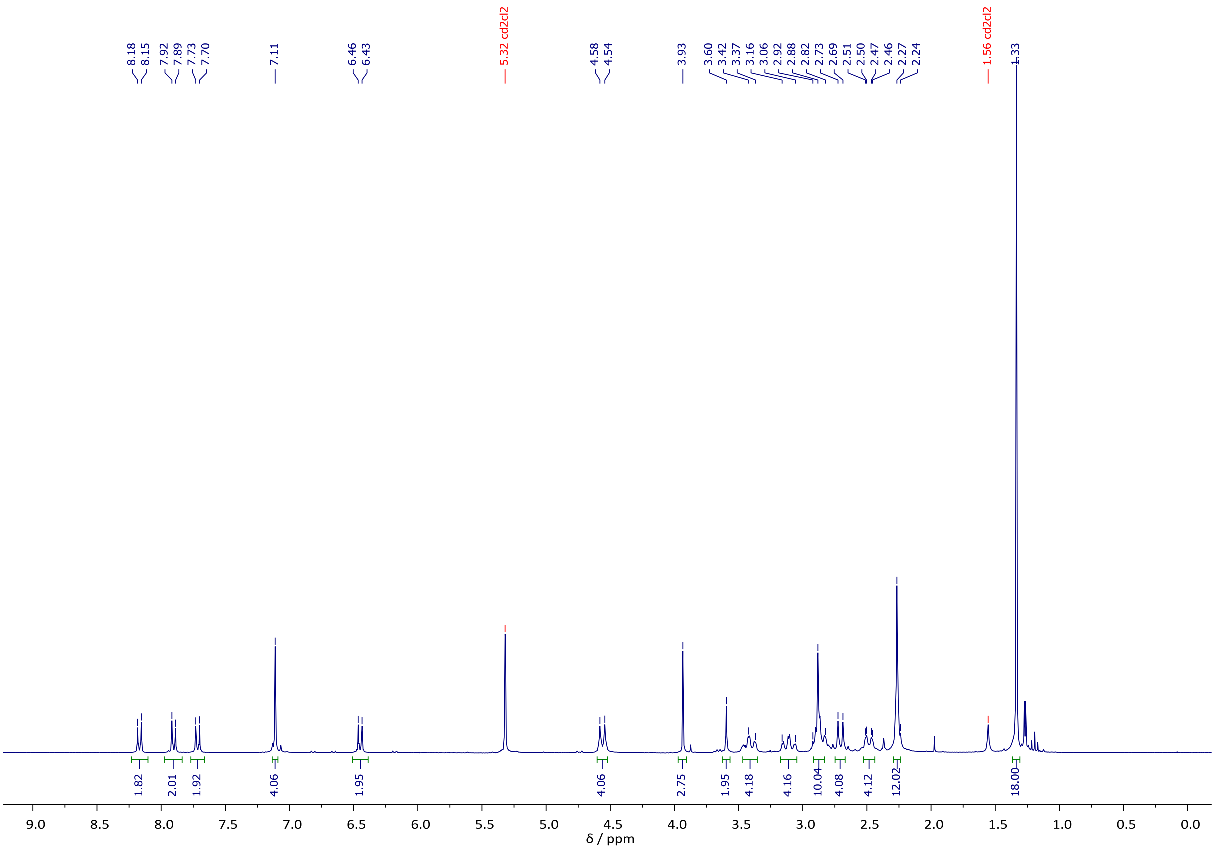

Figure S42. ^{1}H NMR spectrum of **8** in CD_2Cl_2 at ambient temperature.

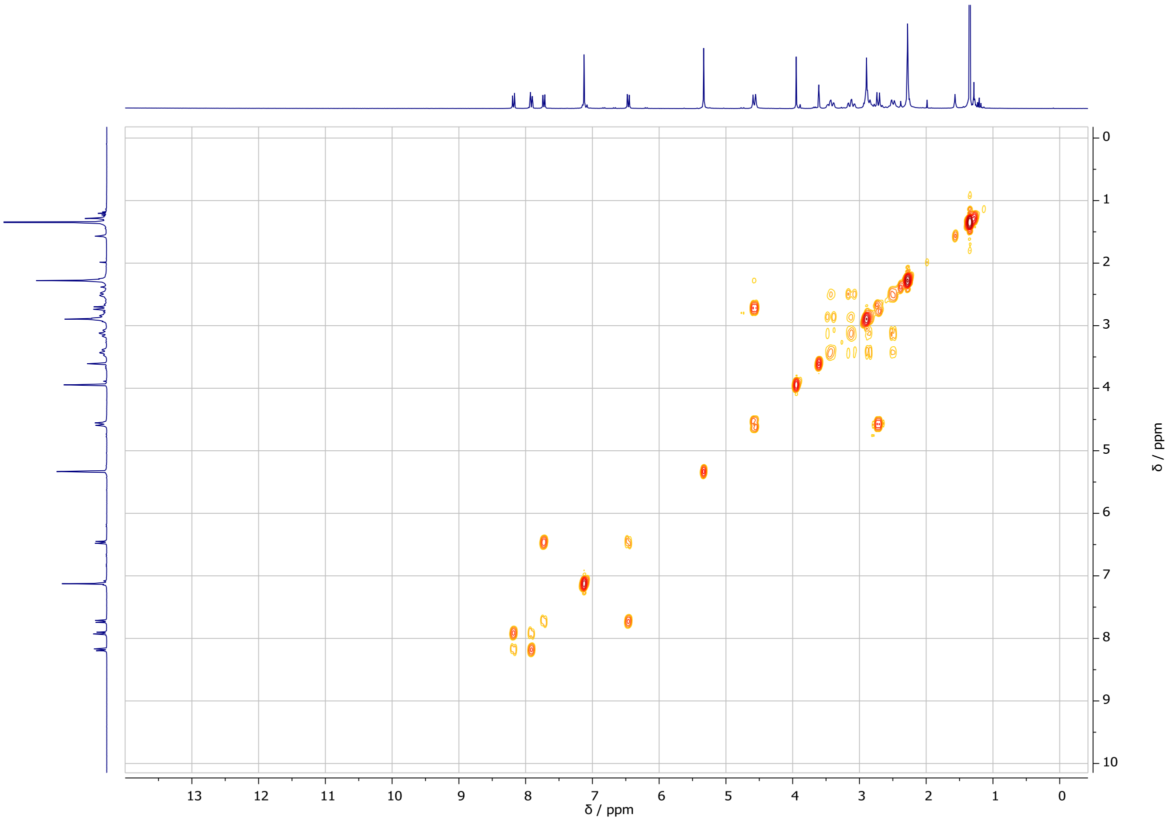

Figure S43. $^{1}H,^{1}H$ COSY spectrum of **8** in CD_2Cl_2 at ambient temperature.

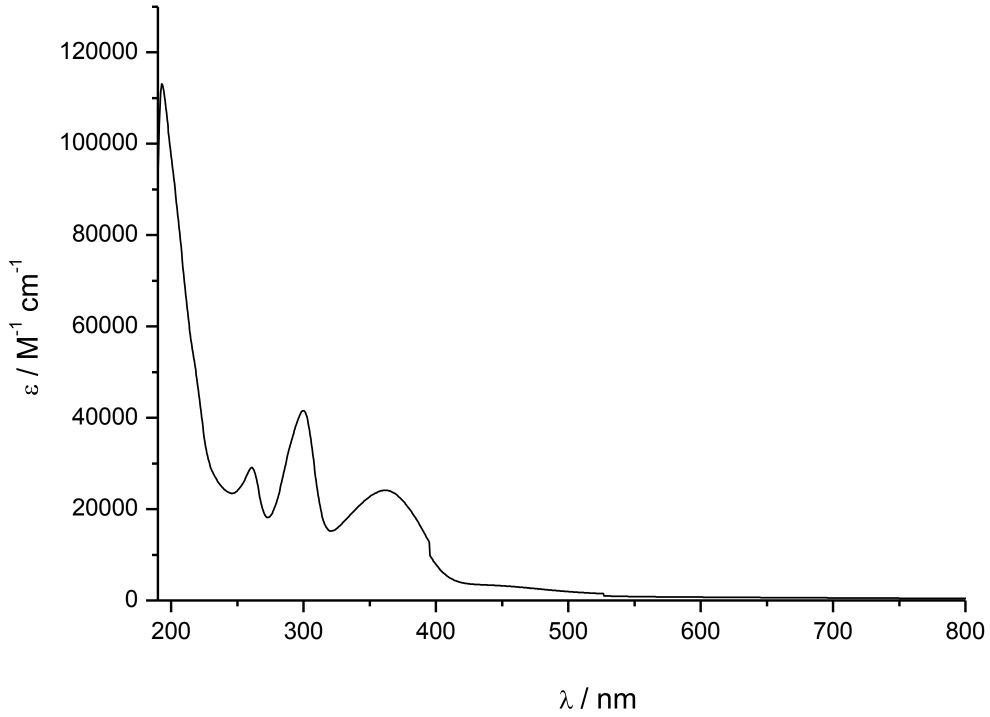

Figure S44. UV–vis spectrum of **8**, (CH_3CN, [**8**] = 10^{-4} M).

9. Analytical data for compound 9.

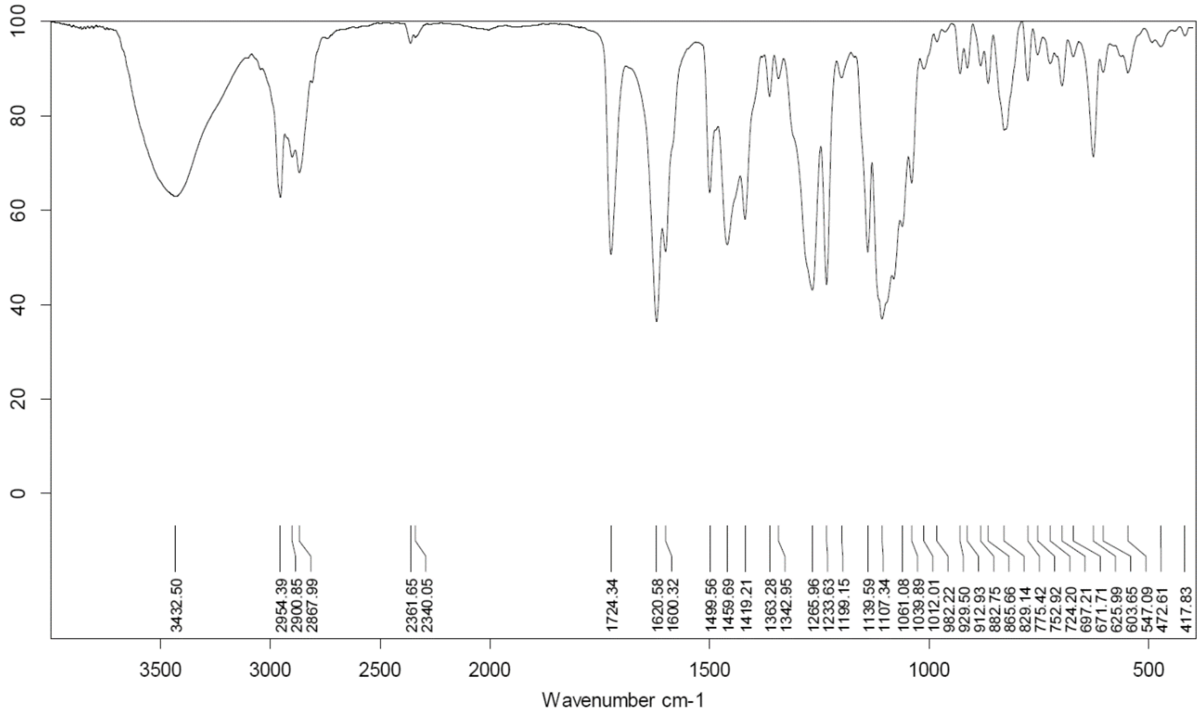

Figure S45. Infrared spectrum of **9**.

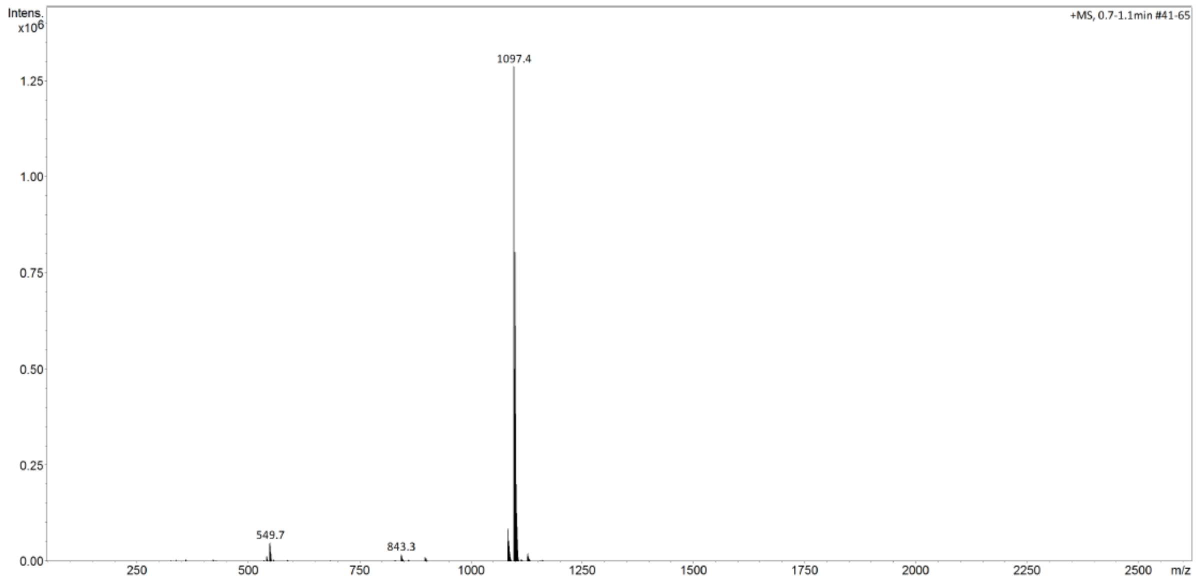

Figure S46. ESI mass spectrum of **9**.

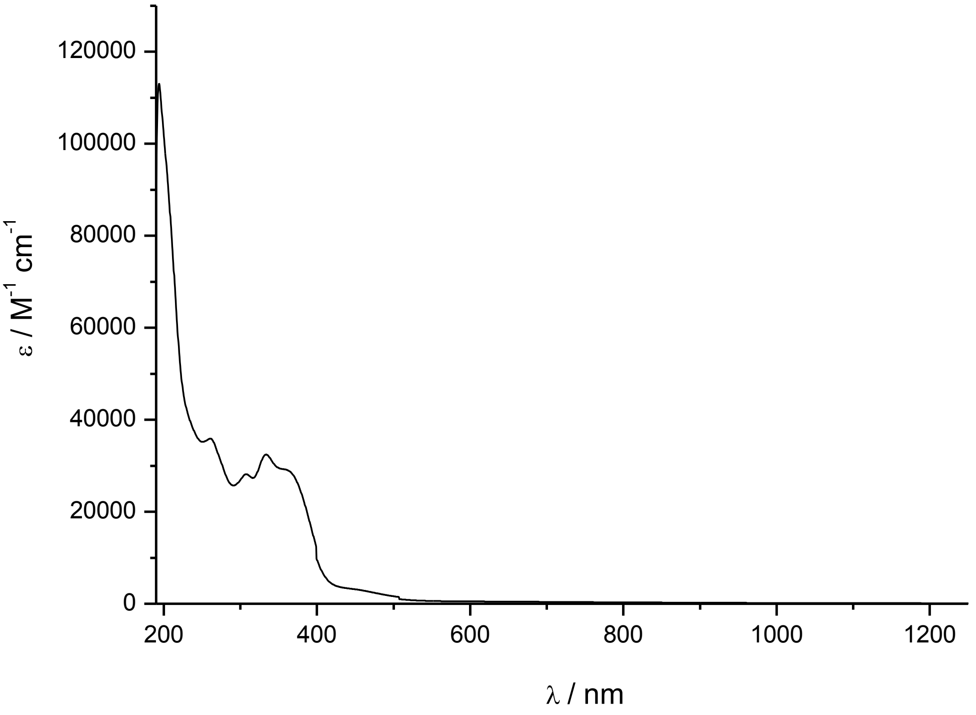

Figure S47. UV–vis spectrum of **9**, (CH_3CN, [**9**] = 10^{-4} M).

10. Selected NMR data

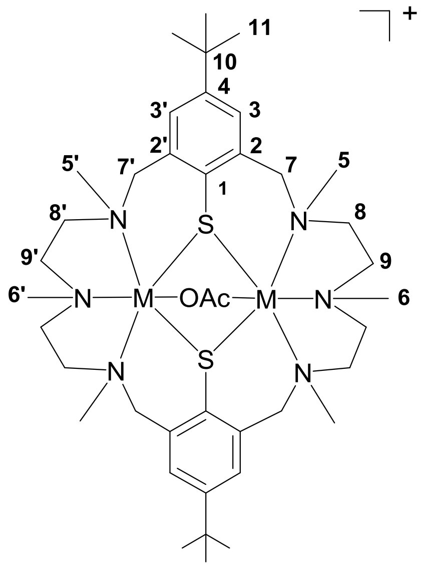

Figure S48. Assignment of carbon atoms of the $[Zn_2L]^{2+}$ or $[Cd_2L]^{2+}$ fragment.

Table S4. Selected ^{1}H NMR spectroscopic data for the zinc (**3**, **5**) and cadmium complexes (**1**, **6**, **8**).[a]

	$[ZnL(OAc)]^{+}$[b][4,10] ($[CdL(OAc)]^{+}$[b][10])	**1**[d]	**3**[c]	**5**[d] **6**[b]	**8**[d]
$C^{11}H_3$	1.28 s (1.23 s)	1.02 s	0.85 s	0.97 s (1.00 s)	1.33 s
C^5H_3	2.48 s (2.36 s)	2.53 s	2.40 s	2.62 s (2.49-2.53 m)	2.27 s
$C^{8,9}H_2$	2.40 m (2.48 m) 2.83 m (2.82 m) 3.29 dt (3.18 dt) 3.52 dt (3.40 dt)	2.56-2.60 m 2.90-2.95 m 3.27 dt 3.49 dt	2.37-2.40 m 2.83-2.85 m 3.32-3.39 m 3.42-3.49 m	2.51-2.55 m (2.49-2.53 m) 2.86-2.89 m (2.84-2.89 m) 3.31 dt (3.25 dt) 3.62 dt (3.46 dt)	2.46-2.51 m 2.82-2.92 m 3.11 dt 3.40 dt
C^7H_2	2.62 d (2.74 d) 4.40 d (4.54 d)	2.75 d 4.64 d	2.62 d 4.33 d	2.68 d (2.73 d) 4.50 d (4.61 d)	2.71 d 4.56 d
C^6H_3	2.92 s (2.92 s)	2.95 s	2.83 s	3.00 s (2.88 s)	2.89 s
C^3H_2	7.13 s (7.12 s)	6.99 s	6.86 s	6.92 s (7.03 s)	7.11 s

[a] NMR data correspond to the ClO_4^- salts. Resonances for the supporting ligands are assigned according to the structure shown in Figure S49. [b] Solvent: CD_3CN. [c] Solvent: $(CD_3)_2SO$. [d] Solvent: CD_2Cl_2.

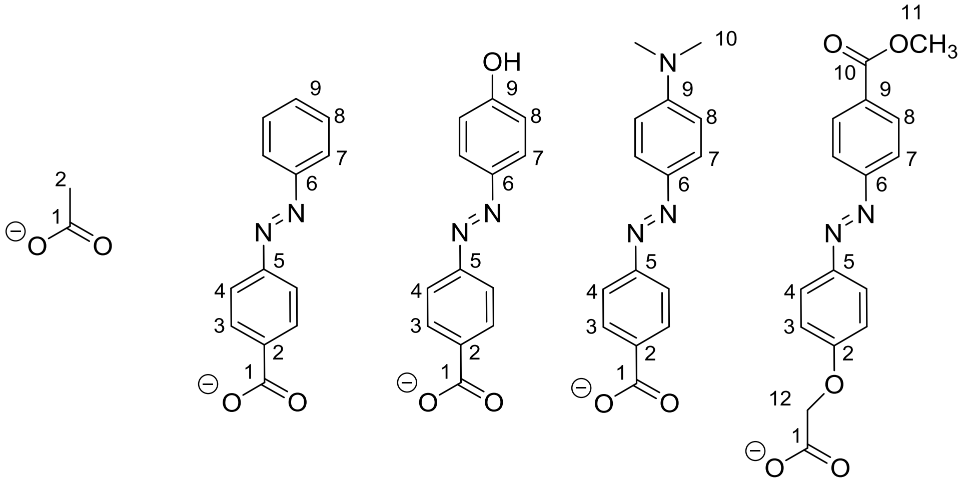

Figure S49. Assignment of carbon atoms of the co-ligands.

Table S5. Selected ^{13}C NMR spectroscopic data for the zinc (**3**, **5**) and cadmium complexes (**1**, **6**).[a]

	$[ZnL(OAc)]^{+}$[b][4,10]<br>($[CdL(OAc)]^{+}$)[c][10]	**1**[e]	**3**[d]	**5**[e]<br>**6**[c]
**$[M_2L]^{2+}$ fragment**				
C^1	143.62<br>(141.50)	140.16	142.10	142.20<br>(141.00)
C^2	135.25<br>(136.10)	135.10	133.46	134.13<br>(135.81)
C^3	128.95<br>(130.50)	130.02	129.09	128.44<br>(130.31)
C^4	145.98<br>(146.40)	146.18	145.13	145.88<br>(146.15)
C^5	46.84<br>(46.70)	46.91	46.03	47.24<br>(46.85)
C^6	50.08<br>(50.30)	50.22	48.73	49.95<br>(50.14)
C^7	64.59<br>(63.00)	62.89	63.03	64.37<br>(62.90)
C^8	59.74<br>(60.40)	60.16	58.15	59.56<br>(60.27)
C^9	58.59<br>(57.9)	57.58	56.91	58.33<br>(57.69)
C^{10}	34.64<br>(34.70)	34.13	33.22	34.07<br>(34.34)
C^{11}	31.68<br>(31.60)	31.20	30.69	31.22<br>(31.20)

Co-ligand fragment				
C1	174.97 (176.3)	169.62	166.52	168.65 (169.94)
C2	22.94 (23.2)	138.16	136.63	136.13 (137.27)
C3	–	130.28	127.12	129.86 (130.95)
C4	–	122.44	121.08	121.66 (121.89)
C5	–	154.25	153.08	154.96 (155.20)
C6	–	153.12	143.69	143.95 (144.16)
C7	–	123.36	124.97	125.56 (125.84)
C8	–	129.68	115.98	111.91 (112.53)
C9	–	131.88	161.24	153.38 (154.05)
C10	–	–	–	40.62 (40.49)

[a] NMR data correspond to the ClO_4^- salts. Resonances for the co-ligands and supporting ligands are assigned according to the structures shown in Figure S49, S50 and Table 2. [b] ^{13}C NMR spectroscopic data recorded for the BPh_4^- salt in $CDCl_3$. [c] Solvent: CD_3CN. [d] Solvent: $(CD_3)_2SO$. [e] Solvent: CD_2Cl_2.

11. Selected metrical data for structurally characterized compounds

Table S6: Selected bond lengths of the crystallographically characterized complexes.

a) $[Zn_2L(\mu\text{-azo-OH})][Zn_2L(\mu\text{-azo-O})]\cdot BPh_4\cdot 4MeCN\cdot 3H_2O$ (**3'** ·4MeCN·3H_2O) and $[Zn_2L(\mu\text{-azo-NMe}_2)]ClO_4\cdot 1.5MeCN$ (**5**·1.5MeCN)

	**3'** $[Zn_2L(\mu\text{-azo-OH})]^+$	**3'** $[Zn_2L(\mu\text{-azo-O})]$	**5** $[Zn_2L(\mu\text{-azo-NMe}_2)]^+$
Zn1-O1	2.044(3)	2.050(2)	2.058(2)
Zn1-N1	2.281(3)	2.400(3)	2.382(3)
Zn1-N2	2.226(3)	2.266(3)	2.221(3)
Zn1-N3	2.403(4)	2.296(3)	2.288(3)
Zn1-S1	2.574(1)	2.515(1)	2.517(1)
Zn1-S2	2.488(1)	2.555(1)	2.572(1)
Zn2-O2	2.049(3)	2.029(3)	2.039(2)
Zn2-N4	2.284(4)	2.398(4)	2.412(3)
Zn2-N5	2.230(4)	2.210(3)	2.222(3)
Zn2-N6	2.400(4)	2.303(3)	2.305(3)
Zn2-S1	2.497(1)	2.602(1)	2.542(1)
Zn2-S2	2.576(1)	2.513(1)	2.511(1)

b) $[Cd_2L(\mu\text{-azo-NMe}_2)]ClO_4\cdot0.5MeCN$ (**6**·0.5MeOH) and $[Cd_2L(\mu\text{–azo-}CO_2Me)]BPh_4\cdot MeCN$ (**8**·MeCN)

	**6** $[Cd_2L(\mu\text{-azo-NMe}_2)]^+$	**8** $[Cd_2L(\mu\text{-azo-}CO_2Me)]^+$
Cd1-O1	2.264(3)	2.253(4)
Cd1-N1	2.477(4)	2.426(4)
Cd1-N2	2.412(3)	2.382(5)
Cd1-N3	2.414(4)	2.479(5)
Cd1-S1	2.624(1)	2.669(1)
Cd1-S2	2.698(1)	2.650(1)
Cd2-O2	2.251(3)	2.259(4)
Cd2-N4	2.471(3)	2.440(4)
Cd2-N5	2.397(4)	2.400(4)
Cd2-N6	2.397(3)	2.447(5)
Cd2-S1	2.699(1)	2.657(1)
Cd2-S2	2.633(1)	2.703(1)

c) $[Ni_2L(\mu\text{-azo-NMe}_2)]ClO_4\cdot xEtOH$ (**7**·xEtOH)

	**7** $[Ni_2L(\mu\text{-azo-NMe}_2)]^+$ Molecule A	**7** $[Ni_2L(\mu\text{-azo-NMe}_2)]^+$ Molecule B
Ni1-O1	2.016(2)	2.010(2)
Ni1-N1	2.247(3)	2.263(3)
Ni1-N2	2.135(3)	2.146(2)
Ni1-N3	2.302(3)	2.281(3)
Ni1-S1	2.487(1)	2.461(1)
Ni1-S2	2.435(1)	2.480(1)
Ni2-O2	2.021(2)	2.017(2)
Ni2-N4	2.224(3)	2.287(3)
Ni2-N5	2.149(3)	2.139(3)
Ni2-N6	2.305(3)	2.241(3)
Ni2-S1	2.454(1)	2.462(1)
Ni2-S2	2.474(1)	2.479(1)

Table S7. Crystallographic data for the complexes.

Compound	**3'**·4MeCN·3H_2O	**5**·1.5MeCN	**6**·0.5MeOH	**7**·0.5MeOH	**8**·MeCN
Formula	$C_{134}H_{177}BN_{20}O_9S_4Zn_4$	$C_{56}H_{82.5}ClN_{10.5}O_6S_2Zn_2$	$C_{53.5}H_{80}Cd_2ClN_9O_{6.5}S_2$	$C_{106}H_{156}Cl_2N_{18}Ni_4O_{12}S_4$	$C_{80}H_{100}BCd_2N_9O_5S_2$
M_r [g/mol]	2612.49	1229.13	1277.64	2308.47	1567.42
Space group	$P\bar{1}$	$P\bar{1}$	$P\bar{1}$	$P\bar{1}$	$P\bar{1}$
a, Å	14.914(3)	13.798(1)	14.213(3)	16.058(8)	15.719(3)
b, Å	17.370(4)	15.969(2)	16.052(3)	19.302(6)	15.985(3)
c, Å	26.776(5)	15.719(1)	16.236(3)	24.059(10)	16.581(3)
α, deg	94.90(3)	70.25(1)	71.37(3)	83.79(3)	109.92(3)
β, deg	92.57(3)	85.69(1)	72.01(3)	79.06(3)	94.43(3)
γ, deg	96.25(3)	72.89(8)	87.35(3)	66.63(3)	100.45(3)
V, $Å^3$	6860(2)	3114.6(5)	3333.0(11)	6716(5)	3808.2(13)
Z	2	2	2	2	2
$d_{calcd.}$, g/cm^3	1.265	1.311	1.273	1.141	1.367
Cryst. size, mm^3	0.20 × 0.15 × 0.15	0.25 × 0.20 × 0.18	0.10 × 0.10 × 0.10	0.25 × 0.16 × 0.12	0.18 × 0.12 × 0.09
μ(Mo Kα), mm^{-1}	0.814	0.935	0.789	0.709	0.670
θ limits, deg	1.86-27.20	1.41-26.90	1.71-26.62	2.46-27.00	1.70-25.00
Measured refl.	64309	25340	27006	55172	27331
Independent refl.	30182	13006	13736	28573	13195
Observed refl.[a]	14785	8570	11288	18598	8997
No. parameters	1546	827	770	1316	907
*R*1[b] (*R*1 all data)	0.0512 (0.1171)	0.0527 (0.1261)	0.0642 (0.0713)	0.0557 (0.0764)	0.0479 (0.0822)
w*R*2[c] (w*R*2 all data)	0.1146 (0.1400)	0.0859 (0.1478)	0.1782 (0.1847)	0.1486 (0.1583)	0.1051 (0.1256)
Max, min peaks, e/$Å^3$	0.877/-0.907	0.856/-0.831	1.493/-2.987	0.833/ -0.835	0.874 / -1.400

[a] Observation criterion: $I > 2\sigma(I)$. [b] $R1 = \Sigma||F_o| - |F_c|| / \Sigma|F_o|$. [c] $wR2 = \{\Sigma[w(F_o^2 - F_c^2)^2]/\Sigma[w(F_o^2)^2]\}^{1/2}$.

12. Irradiation experiments

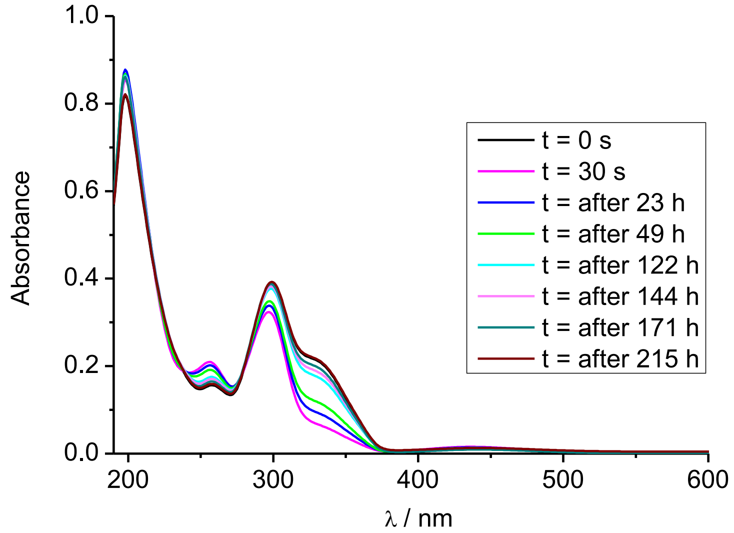

Figure S50. Thermal re-isomerization of the photostationary state of $[Cd_2L(\mu\text{-azo-H})]ClO_4$ (**1**) in MeCN comprising mainly the *cis* (*Z*) form. The spectrum at *t* = 0 s corresponds to a solution of the pure (*E*) form, which was kept for 24 h in the dark prior to measurement. Irradiation of this solution for 30 s produces a photostationary state comprising mainly the (*Z*)-isomer. Concentration of solutions: 10^{-5} M.

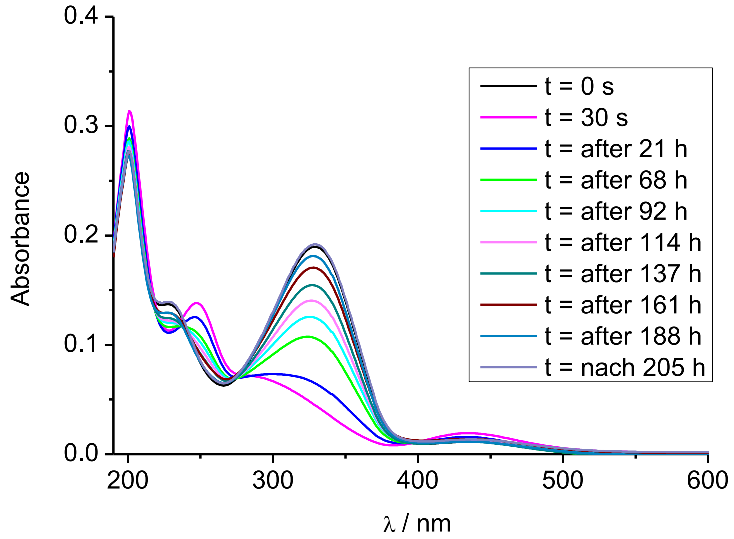

Figure S51. Thermal re-isomerization of the photostationary state of $NBu_4(\mu\text{-azo-H})$ in

MeCN comprising mainly the *cis* (*Z*) form. The spectrum at $t = 0$ s corresponds a solution of the pure (*E*) form, which was kept for 24 h in the dark prior to measurement. Irradiation of this solution for 30 s produces a photostationary state comprising mainly the (*Z*)-isomer. Concentration of solutions: 10^{-5} M.

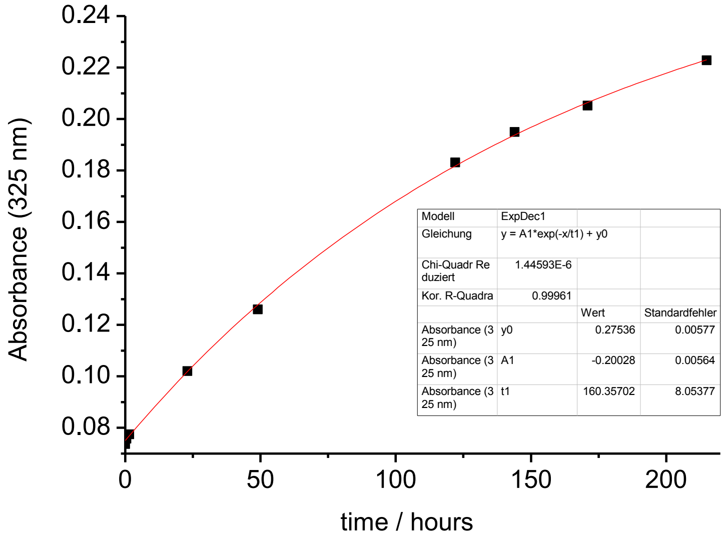

Figure S52. Absorption change at 325 nm of [$Cd_2L(\mu$-azo-H)]ClO_4 (**1**) in acetonitrile upon Z → E isomerization.

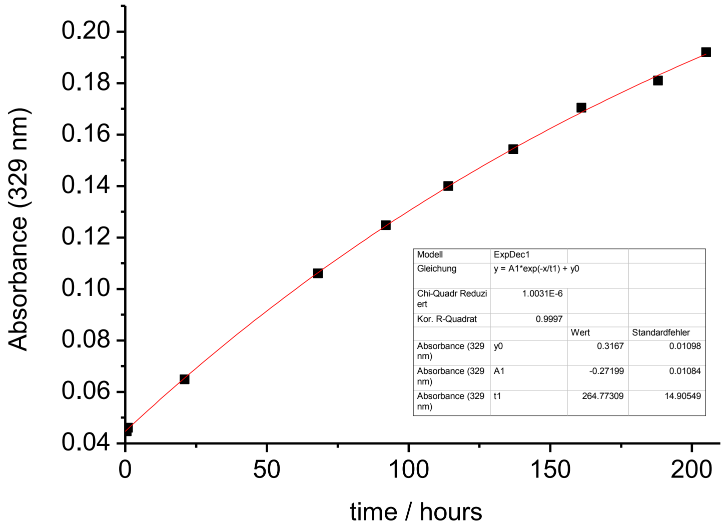

Figure S53. Absorption change at 329 nm of the deprotonated azo-H co-ligand with NBu_4OH in acetonitrile upon Z → E isomerization.

13. Experimental section

CAUTION! *Perchlorate salts are potentially explosive and should therefore be prepared only in small quantities and handled with appropriate care.*

Preparation of [Cd_2L(μ-azo-H)]ClO_4 (1). To a solution of [Cd_2L(μ-Cl)](ClO_4) (164 mg, 0.16 mmol, 1.0 equiv) and the corresponding *p*-azobenzene carboxylic acid (46.8 mg, 0.21 mmol, 1.3 equiv) in a mixture of acetonitrile/methanol (30 mL, 1/2, v/v) triethylamine (28.7 µL, 0.21 mmol, 1.3 equiv) was added. The resulting orange-colored solution was stirred at room temperature for 1 d, after this a solution of $LiClO_4 \cdot 3H_2O$ (256 mg, 1.59 mmol, 10 equiv) dissolved in 10 mL ethanol was added, and stirring was continued for 1 h. The mixture was evaporated under vacuum to a final volume of 5 mL and kept at 4 °C for 1 h. The resulting orange-colored solid was isolated by filtration, washed with cold ethanol and diethyl ether and dried under vacuum. The crude product was purified by recrystallization from a mixed acetonitrile/ethanol solution. Yield: 176 mg (0.14 mmol, 90%); mp > 304 °C (decomp.); Elemental analysis calcd. for $C_{51}H_{73}Cd_2ClN_8O_6S_2$ ([M+ClO_4]; 1218.6): C 50.27, H 6.04, N 9.20; found: C 49.80, H 5.94, N 9.09; ESI^+-MS (CH_3CN) *m/z:* 1119.3 [M^+]; IR (KBr, cm^{-1}): $\tilde{\nu}$ = 3445 (m, br), 3049 (m), 2960 (m), 2900 (m), 2865 (m), 1624 (w), 1599 (s, $\nu_{as}(RCO_2)$), 1563 (m), 1459 (s), 1395 (s, $\nu_s(RCO_2)$), 1368 (w), 1314 (w), 1293 (w), 1269 (w), 1229 (w), 1204 (w), 1154 (w), 1119 (s), 1085 (vs, $\nu(ClO_4^-)$), 1045 (m), 1012 (w), 981 (w), 912 (w), 886 (w), 847 (w), 816 (m), 802 (w), 787 (w), 745 (w), 700 (w), 624 (m), 560 (w), 557 (w), 438 (w); ^{1}H NMR (400 MHz, CH_2Cl_2, for atom labeling see Figure S49 and S50): δ([Cd_2L]$^{2+}$ fragment) = 1.02 (s, 18 H, $C^{11}H_3$), 2.53 (s, 12 H, C^5H_3), 2.56-2.60 (m, 4 H, C^8H), 2.75 (d, $^2J_{H,H}$ = 12.0 Hz, 4 H, C^7HH), 2.90-2.95 (m, 4 H, C^9H), 2.90-2.95 (m, 6 H, C^6H_3), 3.27 (dt, $J_{H,H}$ = 12.0 and 3.0 Hz, 4 H, C^8H), 3.49 (dt, $J_{H,H}$ = 12.0 and 3.0 Hz, 4 H, C^9H), 4.64 (d,

$^{2}J_{H,H}$ = 12.0 Hz, 4 H, $C^{7}HH$), 6.99 (s, 4 H, $C^{3}H$); δ(co-ligand) = 7.48-7.52 (m, 5 H, $C^{9}H$, $C^{8}H$, $C^{3}H$), 7.63 (d, $^{3}J_{H,H}$ = 8.0 Hz, 2 H, $C^{4}H$), 7.86 (dd, $^{3}J_{H,H}$ = 8.0 Hz, 2 H, $C^{7}H$). ^{13}C NMR (400 MHz, CD_3CN, 25 °C): δ($[Cd_2L]^{2+}$ fragment) = 31.20 (C^{11}), 34.13 (C^{10}), 46.91 (C^{5}), 50.22 (C^{6}), 57.58 (C^{9}), 60.16 (C^{8}), 62.89 (C^{7}), 130.01 (C^{3}), 135.04 (C^{2}), 140.15 (C^{1}), 146.17 (C^{4}); δ(co-ligand) = 122.44 (C^{4}), 123.35 (C^{7}), 129.67 (C^{8}), 130.28 (C^{3}), 131.88 (C^{9}), 138.15 (C^{2}), 153.11 (C^{6}), 154.24 (C^{5}), 169.62 (C^{1}) ppm; UV-vis (CH_3CN): λ_{max} [nm] (ε [M^{-1} cm^{-1}]) = 192 (104317), 260 (19844), 300 (42752), 325 sh (23011), 441 (882).

Preparation of [Ni_2L(μ-azo-H)]ClO_4 (2). This compound was prepared from [Ni_2L(μ-Cl)]ClO_4 (157 mg, 0.17 mmol, 1.0 equiv) and *p*-azobenzene carboxylic acid (50.1 mg, 0.22 mmol, 1.3 equiv) by the procedure detailed for **1**. The resulting green-brown solid was isolated by filtration, washed with cold ethanol and diethyl ether, purified by recrystallization from a mixed acetonitrile/ethanol solution and dried in vacuum. Yield: 172 mg (0.16 mmol, 91%); mp > 358 °C (decomp.); Elemental analysis calcd. for $C_{51}H_{73}ClN_8Ni_2O_6S_2$ ([M+ClO_4]; 1111.2): C 55.04, H 6.54, N 9.99; found: C 55.02, H 6.62, N 9.93; ESI^+-MS (CH_3CN) *m/z:* 1011.4 [M^+]; IR (KBr, cm^{-1}): $\tilde{\nu}$ = 3444 (m, br), 2960 (m), 2900 (m), 2866 (m), 2809 (w), 1602 (s, $\nu_{as}(RCO_2)$), 1567 (m), 1462 (s), 1401 (s, $\nu_s(RCO_2)$), 1364 (w), 1308 (w), 1264 (w), 1233 (w), 1202 (w), 1152 (w), 1119 (s), 1104 (vs, $\nu(ClO_4^-)$), 1093 (s), 1080 (s), 1040 (m), 1005 (w), 983 (w), 930 (w), 913 (w), 882 (w), 823 (w), 785 (m), 753 (w), 699 (w), 625 (m), 564 (w), 540 (w), 452 (w), 416 (w); UV-vis (CH_3CN): λ_{max} [nm] (ε [M^{-1} cm^{-1}]) = 195 (88412), 276 sh (24155), 312 (33230), 328 (34994), 439 (1180), 647 (39), 1117 (66).

Preparation of [Zn_2L(μ-azo-OH)]ClO_4 (**3**). To a suspension of $H_2L\cdot 6HCl$ (356 mg, 0.40 mmol, 1.0 equiv) and $ZnCl_2\cdot H_2O$ (181 mg, 0.80 mmol, 2.0 equiv) in methanol (100 mL) triethylamine (444 µL, 3.20 mmol, 8.0 equiv) was added and the resulting colorless solution

was stirred for 30 min. A solution of the corresponding azobenzene carboxylate (145 mg, 0.60 mmol, 1.5 equiv) and triethylamine (83 µL, 0.60 mmol, 1.5 equiv) in methanol (30 mL) was added and the resulting orange-colored solution was stirring for further 2 h. A solution of $LiClO_4 \cdot 3\ H_2O$ (1.07 g, 4.00 mmol, 10.0 equiv) dissolved in 75 mL ethanol was added and the mixture was evaporated under vacuum to a final volume of 5 mL and kept at 4 °C for 1h. The resulting orange-colored solid was filtered off and washed with cold ethanol and diethylether. The crude product was recrystallized from acetonitrile/ethanol and dried in vacuum. Yield: 388 mg (0.34 mmol, 85%); mp > 348 °C (decomp.); Elemental analysis calcd. for $C_{51}H_{73}ClN_8O_7S_2Zn_2$ ([M+ClO_4]; 1140.5): C 53.71, H 6.45, N 9.82; found: C 54.01, H 6.45, N 9.81; ESI$^+$-MS (DMSO/CH_3CN) *m/z:* 1041.3 [M^+]; IR (KBr, cm^{-1}): $\tilde{\nu}$ = 3426 (m), 2961 (m), 2903 (m), 2867 (m), 2361 (w), 1600 (s, $\nu_{as}(RCO_2)$), 1563 (m), 1505 (w), 1462 (s), 1404 (s, $\nu_s(RCO_2)$), 1366 (m), 1308 (w), 1272 (m), 1232 (m), 1120 (s), 1081 (s, $\nu(ClO_4^-)$), 1045 (m), 1009 (w), 928 (w), 914 (w), 885 (w), 847 (w), 823 (m), 783 (w), 751 (w), 697 (w), 673 (w), 626 (m), 597 (w), 545 (w), 488 (w), 435 (w); ^{1}H NMR (400 MHz, $(CD_3)_2SO$, for atom labeling see Figure S49 and S50): δ([Zn_2L]$^{2+}$ fragment) = 0.85 (s, 18 H, $C^{11}H_3$), 2.37-2.40 (m, 4 H, C^8H), 2.40 (s, 12 H, C^5H_3), 2.62 (d, $^2J_{H,H}$ = 12.0 Hz, 4 H, C^7HH), 2.83 (s, 6 H, C^6H_3), 2.83-2.85 (m, 4 H, C^9H), 3.32-3.39 (m, 4 H, C^8H), 3.42-3.49 (m, 4 H, C^9H), 4.33 (d, $^2J_{H,H}$ = 12.0 Hz, 4 H, C^7HH), 6.86 (s, 4 H, C^3H); δ(co-ligand) = 6.86 (d, $^3J_{H,H}$ = 9.0 Hz, 2 H, C^8H), 7.31 (d, $^3J_{H,H}$ = 9.0 Hz, 2 H, C^3H), 7.46 (d, $^3J_{H,H}$ = 9.0 Hz, 2 H, C^4H), 7.67 (d, $^3J_{H,H}$ = 9.0 Hz, 2 H, C^7H); ^{13}C NMR (100 MHz, $(CD_3)_2SO$): δ ([Zn_2L]$^{2+}$ fragment) = 30.69 (C^{11}), 33.22 (C^{10}), 46.05 (C^5), 48.73 (C^6), 56.91 (C^9), 58.15 (C^8), 63.03 (C^7), 129.09 (C^3), 133.46 (C^2), 142.10 (C^1), 145.13 (C^4); δ(co-ligand) = 115.98 (C^8), 121.08 (C^4), 124.97 (C^7), 127.12 (C^3), 136.63 (C^2), 143.69 (C^6), 153.08 (C^5), 161.24 (C^9), 166.52 (C^1); UV/vis (DMSO): λ_{max} [nm] (ε [M^{-1} cm^{-1}]) = 259 (24979), 292 (24378), 366 (28535), 450 (1970). This compound was additionally characterized by X-ray crystallography.

Preparation of [$\mathbf{Ni_2L(\mu\text{-azo-OH})]ClO_4}$ (**4**). To a solution of [$Ni_2L(\mu\text{-}Cl)](ClO_4)$ (156 mg, 0.17 mmol, 1.0 equiv) and the corresponding azobenzene carboxylic acid (53.3 mg, 0.22 mmol, 1.3 equiv) in a mixture of acetonitrile/methanol (30 mL, 1/2, v/v) triethylamine (30.5 µL, 0.22 mmol, 1.3 equiv) was added. The resulting orange/brown-colored solution was stirred at room temperature for 1 d, after this a solution of $LiClO_4 \cdot 3\ H_2O$ (272 mg, 1.69 mmol, 10 equiv) dissolved in 10 mL ethanol was added, and stirring was continued for 1 h. The mixture was evaporated under vacuum to a final volume of 5 mL and kept at 4 °C for 1 h. The resulting green/brown-colored solid was isolated by filtration, washed with cold ethanol and diethyl ether and dried under vacuum. The crude product was purified by recrystallization from a mixed acetonitrile/ethanol solution. Yield: 149 mg (0.13 mmol, 78%); mp > 335 °C (decomp.); Elemental analysis calcd. for $C_{51}H_{73}ClN_8Ni_2O_7S_2 \cdot 2H_2O$ ([$M+ClO_4$]; 1127.1+36.0): C 52.66, H 6.67, N 9.63; found: C 52.38, H 6.32, N 9.60; ESI^+-MS (CH_3CN) *m/z:* 1027.4 [M^+]; IR (KBr, cm^{-1}): $\tilde{\nu}$ = 3439 (m, br), 2960 (m), 2923 (m), 2857 (m), 2809 (m), 1737 (w), 1620 (w), 1590 (s, $\nu_{as}(RCO_2)$), 1566 (m), 1461 (s), 1407 (s, $\nu_s(RCO_2)$), 1365 (m), 1308 (w), 1266 (w), 1242 (w), 1203 (w), 1134 (m), 1120 (vs, $\nu(ClO_4^-)$), 1107 (m), 1077 (m), 1058 (m), 1040 (m), 929 (w), 913 (w), 879 (w), 847 (w), 823 (w), 780 (w), 752 (w), 697 (w), 675 (w), 630 (m), 625 (w), 563 (w), 444 (w), 469 (w); UV-vis (DMSO): λ_{max} [nm] (ε [$M^{-1}\ cm^{-1}$]) = 258 (27612), 311 (20738), 341 (29144), 364 (31733), 454 (2099), 645 (115), 1113 (76).

Preparation of [$\mathbf{Zn_2L(\mu\text{-azo-NMe_2})]ClO_4}$ (**5**). This compound was prepared from $H_2L \cdot 6HCl$ (356 mg, 0.40 mmol, 1.0 equiv), $ZnCl_2 \cdot H_2O$ (181 mg, 0.80 mmol, 2.0 equiv), and Hazo-NMe_2 (162.0 mg, 0.60 mmol, 1.5 equiv) by the procedure detailed for **3**. The crude product was purified by recrystallization from an acetonitrile/ethanol mixture and dried in vacuum. Yield:

420 mg (0.36 mmol, 90%); mp > 358 °C (decomp.); Elemental analysis calcd. for $C_{53}H_{78}ClN_9O_6S_2Zn_2\cdot H_2O$ ([M+ClO$_4$]; 1167.6+18.0): C 53.69, H 6.80, N 10.63; found: C 53.64, H 6.76, N 10.61; ESI$^+$-MS (CH_3CN) *m/z:* 1068.4 [M$^+$]; IR (KBr, cm^{-1}): $\tilde{\nu}$ = 3443 (m), 2958 (m), 2867 (m), 1599 (s, $\nu_{as}(RCO_2)$), 1563 (m), 1518 (m), 1461 (m), 1398 (s, $\nu_s(RCO_2)$), 1363 (s), 1311 (w), 1268 (w), 1232 (w), 1203 (w), 1148 (m), 1120 (m), 1092 (s, $\nu(ClO_4^-)$), 1056 (m), 1009 (w), 928 (w), 913 (w), 883 (w), 847 (w), 822 (m), 782 (w), 750 (w), 735 (w), 699 (w), 661 (w), 625 (m), 596 (w), 536 (w), 479 (w); ^{1}H NMR (400 MHz, CD_2Cl_2, for atom labeling see Figure S49 and S50): δ([Zn_2L]$^{2+}$ fragment) = 0.97 (s, 18 H, $C^{11}H_3$), 2.51-2.55 (m, 4 H, C^8H), 2.61 (s, 12 H, C^5H_3), 2.68 (d, $^2J_{H,H}$ = 12.0 Hz, 4 H, C^7HH), 2.86-2.89 (m, 4 H, C^9H), 3.00 (s, 6 H, C^6H_3), 3.31 (dt, $J_{H,H}$ = 12.0 and 4.0 Hz, 4 H, C^8H), 3.62 (dt, $J_{H,H}$ = 12.0 and 4.0 Hz, 4 H, C^9H), 4.50 (d, $^2J_{H,H}$ = 12.0 Hz, 4 H, C^7HH), 6.92 (s, 4 H, C^3H); δ(co-ligand) = 3.08 (s, 6H, N($C^{10}H3$)$_2$), 6.74 (d, $^3J_{H,H}$ = 8.0 Hz, 2 H, C^8H), 7.34 (d, $^3J_{H,H}$ = 8.0 Hz, 2 H, C^3H), 7.49 (d, $^3J_{H,H}$ = 8.0 Hz, 2 H, C^4H), 7.79 (d, $^3J_{H,H}$ = 8.0 Hz, 2 H, C^7H); ^{13}C NMR (100 MHz, CD_2Cl_2): δ([Zn_2L]$^{2+}$ fragment) = 31.22 (C^{11}), 34.07 (C^{10}), 47.24 (C^5), 49.95 (C^6), 58.33 (C^9), 59.56 (C^8), 64.37 (C^7), 128.44 (C^3), 134.13 (C^2), 142.20 (C^1), 145.88 (C^4); δ(co-ligand) = 40.62 (C^{10}), 111.91 (C^8), 121.66 (C^4), 125.56 (C^7), 129.86 (C^3), 136.13 (C^2), 143.95 (C^6), 153.38 (C^9), 154.96 (C^5), 168.65 (C^1); UV/vis (CH_3CN): λ_{max} [nm] (ε [M^{-1} cm^{-1}]) = 195 (83650), 261 (27080), 285 (31050), 382 sh (11790), 434 (16000). This compound was additionally characterized by X-ray crystallography.

Preparation of [Cd_2L(μ-Azo-NMe$_2$)]ClO$_4$ (6). To a solution of [Cd_2L(μ-Cl)](ClO_4) (184 mg, 0.18 mmol, 1.0 equiv) and the corresponding azobenzene carboxylic acid (62.6 mg, 0.23 mmol, 1.3 equiv) in a mixture of acetonitrile/methanol (30 mL, 1/2, v/v) was added triethylamine (32.2 µL, 0.23 mmol, 1.3 equiv). The resulting red solution was stirred at room temperature for 3 d, after this a solution of $LiClO_4\cdot 3\ H_2O$ (287 mg, 1.79 mmol, 10.0 equiv)

dissolved in 10 mL ethanol was added, and stirring was continued for 1 h. The mixture was evaporated under vacuum to a final volume of 5 mL and kept at 4°C for 1 h. The resulting red solid was isolated by filtration, washed with cold ethanol and diethyl ether and dried under vacuum. The crude product was purified by recrystallization from a mixed acetonitrile/ethanol solution. Yield: 200 mg (0.16 mmol, 88%); mp > 360 °C (decomp.); Elemental analysis calcd. for $C_{53}H_{78}Cd_2ClN_9O_6S_2$ ([M+ClO_4]; 1261.7): C 50.46, H 6.23, N 9.99; found: C 50.27, H 5.99, N 9.91; ESI^+-MS (CH_2Cl_2/CH_3CN) *m/z:* 1162.4 [M^+]; IR (KBr, cm^{-1}): $\tilde{\nu}$ = 3445 (m, br), 3046 (w), 2958 (m), 2863 (m), 2729 (w), 1598 (s, $\nu_{as}(RCO_2)$), 1560 (m), 1518 (m), 1457 (m), 1424 (m), 1401 (s, $\nu_s(RCO_2)$), 1363 (s), 1313 (m), 1293 (m), 1268 (w), 1230 (m), 1204 (w), 1146 (m), 1084 (s, $\nu(ClO_4^-)$), 1045 (m), 1011 (w), 980 (w), 943 (w), 886 (m), 864 (w), 844 (w), 817 (m), 801 (w), 785 (w), 745 (w), 623 (m), 594 (w), 555 (w), 537 (w), 485 (w), 466 (w); ^{1}H NMR (300 MHz, 25°C, CD_3CN, for atom labeling see Figure S49 and S50): δ([$Cd_2L]^{2+}$ fragment) = 1.00 (s, 18 H, $C^{11}H_3$), 2.49-2.53 (m, 12 H, C^5H_3), 2.49-2.53 (m, 4 H, C^8H), 2.73 (d, $^2J_{H,H}$ = 12.0 Hz, 4 H, C^7HH), 2.88 (m, 6 H, C^6H_3), 2.84-2.89 (m, 4 H, C^9H), 3.25 (dt, $J_{H,H}$ = 12.0 and 3.0 Hz, 4 H, C^8H), 3.46 (dt, $J_{H,H}$ = 12.0 and 3.0 Hz, 4 H, C^9H), 4.61 (d, $^2J_{H,H}$ = 12.0 Hz, 4 H, C^7HH), 7.03 (s, 4 H, C^3H); δ(co-ligand) = 3.06 (s, 6H, N($C^{10}H3$)$_2$), 6.81 (d, $^3J_{H,H}$ = 9.2 Hz, 2 H, C^8H), 7.51 (s, 4 H, C^3H, C^4H), 7.77 (d, $^3J_{H,H}$ = 9.2 Hz, 2 H, C^7H). ^{13}C NMR (100 MHz, CD_3CN, 25 °C): δ([$Cd_2L]^{2+}$ fragment) = 31.20 (C^{11}), 34.34 (C^{10}), 46.85 (C^5), 50.14 (C^6), 57.69 (C^9), 60.27 (C^8), 62.90 (C^7), 130.31 (C^3), 135.81 (C^2), 141.00 (C^1), 146.15 (C^4); δ(co-ligand) = 40.49 (C^{10}), 112.53 (C^8), 121.89 (C^4), 125.84 (C^7), 130.95 (C^3), 137.27 (C^2), 144.16 (C^6), 154.05 (C^9), 155.20 (C^5), 169.94 (C^1) ppm. UV/vis (CH_3CN): λ_{max} [nm] (ε [M^{-1} cm^{-1}]) = 193 (129340), 261 (30525), 298 (38705), 381 (12432), 434 (16292). This compound was additionally characterized by X-ray crystallography.

Preparation of [Ni_2L(μ-azo-NMe_2)]ClO_4 (7). This compound was prepared from [Ni_2L(μ-

Cl)]ClO_4 (181 mg, 0.19 mmol, 1.0 equiv) and Hazo-NMe_2 (68.8 mg, 0.26 mmol, 1.3 equiv) by the procedure detailed for **6**. The resulting red-brown solid was isolated by filtration, washed with cold ethanol and diethyl ether, purified by recrystallization from a mixed acetonitrile/ethanol solution and dried in vacuum. Yield: 189 mg (0.16 mmol, 83%); mp > 345 °C (decomp.); Elemental analysis calcd. for $C_{53}H_{78}ClN_9Ni_2O_6S_2 \cdot 3\ H_2O$ ([M+ClO_4]; 1154.2 + 54.1): C 52.69, H 7.01, N 10.43; found: C 52.32, H 6.43, N 10.24; ESI^+-MS (CH_2Cl_2/CH_3CN) *m/z:* 1054.4 [M^+]; IR (KBr, cm^{-1}): $\tilde{\nu}$ = 3446 (m, br), 2956 (m), 2863 (m), 1599 (vs, $\nu_{as}(RCO_2)$), 1565 (m), 1518 (m), 1461 (m), 1400 (s, $\nu_s(RCO_2)$), 1362 (s), 1310 (m), 1264 (w), 1232 (w), 1201 (w), 1147 (m), 1093 (s, $\nu(ClO_4^-)$), 1040 (m), 1003 (w), 982 (w), 929 (w), 882 (w), 851 (w), 822 (m), 781 (w), 751 (w), 696 (w), 662 (m), 562 (w), 535 (w), 470 (w), 415 (w); UV/vis (CH_3CN): λ_{max} [nm] (ε [M^{-1} cm^{-1}]) = 194 (114417), 267 (37868), 301 sh (24544), 328 sh (20921), 380 (16567), 431 (19801), 650 (221), 1112 (186). This compound was additionally characterized by X-ray crystallography.

Preparation of [Cd_2L(μ-azo-CO_2Me)]ClO_4 (8). To a suspension of [Cd_2L(μ-Cl)]ClO_4 (96.5 mg, 0.09 mmol, 1.0 equiv) and the corresponding azobenzene carboxylic acid (38.1 mg, 0.12 mmol, 1.3 equiv) in a mixture of acetonitrile/methanol (30 mL, 1/2, v/v) was added triethylamine (16.8 μL, 0.12 mmol, 1.3 equiv), and the resulting solution was stirred for 12 h. A solution of $LiClO_4 \cdot 3\ H_2O$ (149.7 mg, 0.93 mmol, 10.0 equiv) in 10 mL ethanol was added and stirring was continued for 1 h. The mixture was evaporated under vacuum to a final volume of 5 mL and kept at 4 °C for 1 h. The resulting orange-colored solid was isolated by filtration, washed with cold ethanol and diethyl ether, purified by recrystallization from a mixed acetonitrile/ethanol solution and dried in vacuum. Yield: 93 mg (0.07 mmol, 76%); mp > 304 °C (decomp.); Elemental analysis calcd. for $C_{54}H_{77}Cd_2ClN_8O_9S_2$ ([M+ClO_4]; 1306.7): C 49.64, H 5.94, N 8.58; found: C 49.36, H 5.55, N 8.19; ESI^+-MS (CH_2Cl_2/CH_3OH) *m/z:*

1207.4 [M$^+$]; IR (KBr, cm^{-1}): $\tilde{\nu}$ = 3512 (m, br), 3433 (m, br), 3048 (w), 2955 (m), 2901 (m), 2865 (m), 2726 (w), 1724 (s), 1614 (vs, $\nu_{as}(RCO_2)$), 1500 (s), 1455 (s), 1412 (s, $\nu_s(RCO_2)$), 1366 (m), 1340 (m), 1276 (s), 1231 (s), 1199 (w), 1140 (s), 1096 (vs, $\nu(ClO_4^-)$), 1044 (s), 1011 (m), 980 (w), 961 (w), 913 (m), 886 (m), 865 (m), 839 (m), 817 (m), 775 (m), 745 (w), 724 (w), 698 (m), 623 (m), 599 (w), 550 (m), 469 (w), 448 (w); ^{1}H NMR (400 MHz, CD_2Cl_2, for atom labeling see Figure S49 and S50): δ($[Cd_2L]^{2+}$ fragment) = 1.33 (s, 18 H, $C^{11}H_3$), 2.27 (s, 12 H, C^5H_3), 2.46-2.51 (m, 4 H, C^8H), 2.71 (d, $^2J_{H,H}$ = 15.6 Hz, 4 H, C^7HH), 2.89 (s, 6 H, C^6H_3), 2.82-2.92 (m, 4 H, C^9H), 3.11 (dt, 4 H, C^8H), 3.40 (dt, 4 H, C^9H), 4.56 (d, $^2J_{H,H}$ = 15.3 Hz, 4 H, C^7HH), 7.11 (s, 4 H, C^3H); δ(co-ligand) = 3.60 (s, 2 H, $OC^{12}H_2$), 3.93 (s, 3 H, $OC^{11}H_3$), 6.44 (d, $^3J_{H,H}$ = 11.6 Hz, 2 H, C^3H), 7.72 (d, $^3J_{H,H}$ = 11.6 Hz, 2 H, C^4H), 7.91 (d, $^3J_{H,H}$ = 12 Hz, 2 H, C^8H), 8.17 (d, $^3J_{H,H}$ = 9.2 Hz, 2 H, C^7H); UV/vis (CH_3CN): λ_{max} [nm] (ε [M^{-1} cm^{-1}]) = 193 (113128), 261 (29102), 300 (33143), 370 (6018), 447 sh (2949). This compound was additionally characterized by X-ray crystallography.

Preparation of $[Ni_2L(\mu\text{-Azo-}CO_2Me)]ClO_4$ (9). This compound was prepared from $[Ni_2L(\mu\text{-}Cl)]ClO_4$ (184 mg, 0.20 mmol, 1.0 equiv) and Hazo-CO_2Me (81.6 mg, 0.26 mmol, 1.3 equiv) by the procedure detailed for **8**. The resulting green-brown solid was isolated by filtration, washed with cold ethanol and diethyl ether, purified by recrystallization from a mixed acetonitrile/ethanol solution and dried in vacuum. Yield: 211 mg (0.18 mmol, 88 %); mp > 341° C (decomp.); Elemental analysis calcd. for $C_{54}H_{77}ClN_8Ni_2O_9S_2 \cdot 3\ H_2O$ ([M+ClO_4]; 1196.4+54.1): C 51.75, H 6.68, N 8.94; found: C 52.02, H 6.04, N 8.56; ESI$^+$-MS (CH_2Cl_2/CH_3OH) *m/z:* 1097.4 [M$^+$]; IR (KBr, cm^{-1}): $\tilde{\nu}$ = 3432 (m, br), 2954 (m), 2900 (m), 2867 (m), 2361 (w), 2340 (w), 1724 (m), 1620 (s, $\nu_{as}(RCO_2)$), 1600 (m), 1499 (m), 1459 (m), 1419 (s, $\nu_s(RCO_2)$), 1363 (w), 1342 (w), 1265 (s), 1233 (s), 1199 (w), 1139 (m), 1107 (s, $\nu(ClO_4^-)$), 1061 (m), 1039 (m), 1012 (w), 982 (w), 929 (w), 912 (w), 882 (w), 865 (w), 829

(m), 775 (w), 752 (w), 724 (w), 697 (w), 671 (w), 625 (m), 603 (w), 547 (w), 472 (w), 417 (w); UV/vis (CH_3CN): λ_{max} [nm] (ε [M^{-1} cm^{-1}]) = 194 (113002), 261 (35911), 308 (28168), 334 (32443), 360 sh (29137), 448 (3186), 645 sh (470), 1113 (153).
